# Phylogeny and evolution of chloroplast tRNAs in Adoxaceae

**DOI:** 10.1002/ece3.7133

**Published:** 2021-01-06

**Authors:** Qiu‐Yi Zhong, Xiao‐Gang Fu, Ting‐Ting Zhang, Tong Zhou, Ming Yue, Jian‐Ni Liu, Zhong‐Hu Li

**Affiliations:** ^1^ Shaanxi Key Laboratory for Animal Conservation Key Laboratory of Resource Biology and Biotechnology in Western China Ministry of Education College of Life Sciences Northwest University Xi'an China; ^2^ Key Laboratory for Plant Diversity and Biogeography of East Asia Kunming Institute of Botany Chinese Academy of Sciences Kunming China; ^3^ Department of Geology State Key Laboratory of Continental Dynamics Early Life Institute Northwest University Xi'an China

**Keywords:** anticodon, chloroplast tRNA, intron, phylogeny, transition/transversion

## Abstract

Chloroplasts are semiautonomous organelles found in photosynthetic plants. The major functions of chloroplasts include photosynthesis and carbon fixation, which are mainly regulated by its circular genomes. In the highly conserved chloroplast genome, the chloroplast transfer RNA genes (cp tRNA) play important roles in protein translation within chloroplasts. However, the evolution of cp tRNAs remains unclear. Thus, in the present study, we investigated the evolutionary characteristics of chloroplast tRNAs in five Adoxaceae species using 185 tRNA gene sequences. In total, 37 tRNAs encoding 28 anticodons are found in the chloroplast genome in Adoxaceae species. Some consensus sequences are found within the Ψ‐stem and anticodon loop of the tRNAs. Some putative novel structures were also identified, including a new stem located in the variable region of tRNA^Tyr^ in a similar manner to the anticodon stem. Furthermore, phylogenetic and evolutionary analyses indicated that synonymous tRNAs may have evolved from multiple ancestors and frequent tRNA duplications during the evolutionary process may have been primarily caused by positive selection and adaptive evolution. The transition and transversion rates are uneven among different tRNA isotypes. For all tRNAs, the transition rate is greater with a transition/transversion bias of 3.13. Phylogenetic analysis of cp tRNA suggested that the type I introns in different taxa (including eukaryote organisms and cyanobacteria) share the conserved sequences “U‐U‐x2‐C” and “U‐x‐G‐x2‐T,” thereby indicating the diverse cyanobacterial origins of organelles. This detailed study of cp tRNAs in Adoxaceae may facilitate further investigations of the evolution, phylogeny, structure, and related functions of chloroplast tRNAs.

## Graphical abstract

In this study, we determined the evolution of chloroplast tRNA genes of five selected Adoxaceae species. We found that synonymous tRNAs may evolved from multiple ancestors. During the evolution process, the dominated tRNA duplications were mainly caused by positive selection and adaptive evolution, while tRNA transfers and losses by various complex mechanisms.

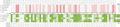

## 1 BACKGROUND

Chloroplasts are multifunctional and semiautonomous organelles found in photosynthetic land plants, and they play an essential role in photosynthesis and carbon fixation (Wicke et al., 2011; Wise & Hoober, 2007). Chloroplasts are metabolic synthesis centers in the cytoplasm, responsible for the synthesis of nucleotides, amino acids, fatty acids, vitamins, and phytohormones (Blee & Joyard, 1996; Noctor et al., 1998; Spetea et al., 2004). These metabolic processes are mainly organized by several circular and independent genomes in the chloroplast, called “chloroplast genomes” (cp DNAs or plastid genomes) (Daniell et al., 2016; Salinas‐Giegé et al., 2015; Wicke et al., 2011). Due to free recombination, uniparental inheritance, and the low mutation rate of nucleotide substitutions, the chloroplast genomes in most photosynthetic angiosperm plants are remarkably conserved in size, gene content, and gene structure. cp DNA is double‐stranded circular DNA measuring 120–160 kb in size, which contains protein‐coding genes (CDSs), tRNAs, rRNAs, and several open reading frames. cp DNA can be divided into four typical regions: a large single copy (LSC), a small single copy (SSC), and two inverted repeats (IRs) (Ravi et al., 2008; Wicke et al., 2011; Zhang et al., 2014). These features have been used widely in phylogenetic and evolutionary studies in recent years (Korpelainen, 2004; Ravi et al., 2008).

Similar to the nuclear genome, the chloroplast genome encodes transfer RNA genes (tRNA genes), which are critical for protein translation in the chloroplast (Daniell et al., 2016; Salinas‐Giegé et al., 2015). tRNAs are short noncoding RNAs, usually comprising 75–95 nucleotides (nt). They are present in all organisms ranging from prokaryotes to eukaryotes, and they evolved in the age of cyanobacteria (Mohanta et al., 2017). tRNA polynucleotide sequence can self‐fold into an L‐shaped tertiary structure to form a hydrogen‐bonded clover leaf‐like structure, which subsequently organizes into a double‐stranded helix (Holley et al., 1965; Wilusz, 2015). Typically, the tertiary structure of tRNA comprises an acceptor arm, dihydrouridine arm (D‐arm), dihydrouridine loop (D‐loop), anticodon arm, anticodon loop, variable loop, pseudouridine arm (Ψ‐arm), and pseudouridine loop (Ψ‐loop). Except for the irregular variable loop, which measures 4–23 nt in length, the nucleotide sequence length of each part is usually conserved among different species (Kirchner & Ignatova, 2015). Moreover, the functions of tRNA are associated with its clover leaf‐like tertiary structure. During the protein translation process, messenger RNAs (mRNAs) serve as templates to direct the synthesis of peptides from amino acids carried by tRNAs. Four different types of tRNA genes have been reported, that is, nonintronic tRNA, intron‐containing tRNA, permuted tRNA, and split tRNA genes (Chan et al., 2011; Randau et al., 2005). The intron‐containing, permuted, and split tRNA genes are also called “disrupted tRNA,” and they evolved from the preceding nonintronic genes (Kanai, 2015; Sugahara et al., 2009). In addition, both the genomic tag hypothesis and phylogenetic analysis suggest that the 3′ half of tRNA was the first to evolve. The tRNA mini helix forms the 3′ half of the traditional tRNA (the acceptor arm and Ψ‐arm) and serves as the substrate for aminoacyl‐tRNA synthetases and CCA‐adding enzymes; therefore, it is considered to be the most ancient form of tRNA (Sun & Caetano‐Anollés, 2008; Weiner & Maizels, 1987, 1999). Though highly conserved, a recent study of cp tRNAs in monocot species found some novel features, including a type I intron and a CAU anticodon for tRNA^Ile^ (Mohanta et al., 2019). In addition, studies of the modification of tRNA wobble nucleotides have provided some important insights recently, including various aspects of modifying substances, processes, and evolution (Delannoy et al., 2009; Huang et al., 2005; Wang et al., 2018). Recently, several studies have investigated tRNA conservation and evolution (Kirchner & Ignatova, 2015; Mohanta et al., 2019). However, the phylogenetic relationships and evolutionary characteristics of chloroplast tRNAs among species are still largely unknown, especially at the family level.

Adoxaceae L. is an ancient family of Dipsacales, initially recorded by Linnaeus in 1753 (Liang & Wu, 1995). Traditionally, Adoxaceae comprises three genera: *Adoxa* L., *Tetradoxa* C. Y. Wu, and *Sinadoxa* C. Y. Wu & Z. L. Wu (Liang & Wu, 1995; Mao et al., 2005). Based on recent studies in embryology, ontogeny, and phylogeny, and the APG III classification system, the traditional Adoxaceae was found to have a close relationship with *Sambucus* L. and *Viburnum* L., which were originally placed in Caprifoliaceae P. Mill. (Liang & Wu, 1995). Therefore, the latter two genera should be added to Adoxaceae to form the modern Adoxaceae, which contains about 200 species (Angiosperm Phylogeny Group, 2009; Zhang et al., 2003). Adoxaceae species are widely used in medicine and horticulture. In recent studies, phylogenetic trees constructed using*ITS*, *trnL*‐F, *ndhF*, and complete chloroplast genome sequences all suggested that Adoxaceae is a monophyletic group. Within Adoxaceae, *Viburnum* and *Sambucus* are the most closely related and the earliest diverged lineages (Donoghue et al., 2001; Eriksson & Donoghue, 1997; Fan et al., 2018; Winkworth et al., 2008; Zhang et al., 2002, 2003). Among the divergences of *Sinadoxa*, *Tetradoxa*, and *Adoxa*, *Sinadoxa* was most probably the first to segregate (Fan et al., 2018; Mao et al., 2005). In addition, the whole chloroplast genomes of several Adoxaceae species are now available, thereby providing a large amount of data for evolutionary analysis (Wang et al., 2016). According to a previous study, the chloroplast genomes of Adoxaceae species range in size from 157,074 bp (*Sinadoxa corydalifolia*) to 158,305 bp (*Sambucus williamsii*). They share a typical quadripartite structure and encode 129 functional genes comprising 37 tRNA genes, 84 CDSs, and eight ribosomal RNA genes (rRNA genes) (Fan et al., 2018). Adoxaceae is a small family with five genera and a total of 200 species, with high economic value. Adoxaceae is suitable for prospective studies of the evolution of chloroplast tRNA at the family level.

In the present study, we conducted a detailed investigation of the chloroplast tRNA genes in Adoxaceae species. In particular, we analyzed 185 tRNA genes in the chloroplast genomes of five Adoxaceae species. The structure, phylogeny, and evolutionary characteristics of the cp tRNAs were analyzed using bioinformatics software. The aims of this study were (a) to determine the conservation and variation of cp tRNAs, and (b) to resolve the phylogenetic and evolutionary patterns of cp tRNAs in Adoxaceae.

## 2 MATERIALS AND METHODS

### 2.1 Identification of tRNA genes

Five whole chloroplast genomes were downloaded from the National Center for Biotechnology Information (NCBI, https://www.ncbi.nlm.nih.gov/), and their original annotations were retained. The five species selected belonged to five different Adoxaceae genera: *Adoxa moschatellina* L. (KX258652), *Sinadoxa corydalifolia* C. Y. Wu et al. (KX258651), *Tetradoxa omeiensis* C. Y. Wu (KX258653), *Sambucus williamsii* Hance (KX510276), and *Viburnum utile* Hemsl. (KX792264). In addition, five whole chloroplast genomes of *Viburnum* species were downloaded from NCBI for analyses at the genus level, including *Viburnum betulifolium* Batal. (MG738665), *V. japonicum* (Thunb.) Sprengel (MH036493), *V. erosum* Thunb. (MN641480), *V. carlesii* Hemsl. × *V. macrocephalum* Fort. (MN985820), and *V. rhytidophyllum* Hemsl. (MT374829). cp tRNA gene sequences were extracted without intergenic regions using the GENEIOUS 8.0.2. program. Subsequently, the segments of the sequences were subjected to further analyses. The online tRNAscan‐SE server was used to predict the lengths and types of the tRNA genes, and to estimate how these predictions are similar to the consensus profile represented by the covariance model (Lowe & Chan, 2016). In tRNAscan‐SE, the following parameters were set according to the bacterial origin of chloroplast (Ravi et al., 2008; Wicke et al., 2011) and based on a previous study (Mohanta et al., 2019): sequence source = bacterial; search mode = default; query sequences = formatted (FASTA); genetic code for tRNA isotype prediction = universal. All of the tRNA genes were analyzed using the same parameters described above. The nucleotide sequences of each tRNA structure (arm or loop) are listed in Table S1. tRNAs with special structures that differ from the traditional clover leaf‐like tRNA are considered as putative novel tRNAs.

### 2.2 Multiple sequence alignment

In nature, the 20 amino acids comprising proteins are called standard amino acids. Unlike other amino acids, they each have a genetic code that can bind to the 20 tRNA isotypes. In this study, the 185 tRNA genes were divided into various groups according to their isotypes for further comparative analyses of the conservation and variations in the sequence or structure. Subsequently, the tRNA gene sequences were subjected to multiple sequence alignment using the program MAFFT v 7.017 in GENEIOUS 8.0.2 (Katoh et al., 2002). The following parameters were used in GENEIOUS 8.0.2: align sequences using = MAFFT; algorithm = auto (select an appropriate strategy from L‐INS‐I, FFT‐NS‐I, and FFT‐NS‐2 according to the data size); scoring matrix = 200PAM/*k* = 2; gap open penalty = 1.53; offset value = 0.123.

### 2.3 Phylogenetic analysis of chloroplast tRNAs

A gene tree was constructed comprising the 185 cp tRNAs from the five Adoxaceae species using maximum‐likelihood (ML) analysis. We also performed phylogenetic analysis of the chloroplast genomes (from which the tRNA sequences were removed) of the five Adoxaceae species using ML analysis to construct a species tree. Both of the ML analyses were performed with RaxML‐HPC2 on CIPRES Science Gateway (https://www.phylo.org/portal2/home.action) using the General Time Reversible model of nucleotide substitution and the gamma model of rate heterogeneity (GTRGAMMA) with 1,000 bootstrap replicates. The two trees were compared visually using FigTree v1.4.1 (Rambaut, 2012) and Illustrator CS6 (Adobe Systems Incorporated).

### 2.4 Analyses of tRNA gene evolution events

A gene tree comprising the 185 cp tRNA genes and a species tree were reconciled using NOTUNG 2.9 (http://www.cs.cmu.edu/~durand/Notung/) and GeneRax v1.2.2 (https://github.com/BenoitMorel/GeneRax), respectively, to analyze duplication, loss, and transfer events (DTL) during the evolution of the cp tRNA genes in Adoxaceae (Benoit et al., 2020; Chen et al., 2000). NOTUNG is based on parsimony method, while GeneRax is based on ML method. Both employ the species‐tree‐aware (STA) approach based on a putative species tree (Stamatakis, 2006). In NOTUNG, the bootstrap‐support threshold was set as the default value (90%). In GeneRax, the parsimony weights are not required and the strategy, probabilistic model, and the maximum radius used for SPR moves in tree search were set to the default: strategy = SPR; probabilistic model = undatedDTL; max‐spr‐radius = 5. We also constructed a species tree based on the chloroplast genomes (from which the tRNA sequences were removed) of seven Dipsacales species (including the five Adoxaceae species mentioned above and two Caprifoliaceae species: *Triosteum pinnatifidum* [MG738666] and *Weigela florida* [MG738664]) using the RAxML v7.2.8 program with 1,000 bootstrap replicates (Stamatakis, 2006). The topology of the species tree was consistent with those reported previously (Fan et al., 2018; Mao et al., 2005). Images of the two reconciled phylogenetic trees were generated by NOTUNG 2.9 (Chen et al., 2000).

### 2.5 Codon usage

CDSs were extracted from the chloroplast genomes of the five Adoxaceae species to measure the codon usage bias. The codon usage bias was evaluated by measuring the codon adaptation index (CAI) and the value of the relative adaptiveness of each codon (CAI‐w). The CAI is a measure of the relative adaptiveness of the codon usage for a gene with respect to the codon usage of highly expressed genes (Peden, 2000). The CAI‐w is the ratio of the usage of each codon relative to that of the most abundant codon for the same amino acid, where the values range between 0 and 1. The CAI‐w value for the most abundant codon is 1.0. The average CAI‐w values were calculated for each species as the weighted mean of all the CAI‐w values associated with the chloroplast tRNAs in a species. This analysis was performed with CODONW 1.4.2 (Peden, 2000).

### 2.6 Nucleotide substitutions

The tRNA genes were subjected to nucleotide transition/transversion analyses in groups according to their isotypes using MEGA 7.0. The following parameters were used in MEGA 7.0 (Kumar et al., 2016; Tamura, 1992): analysis = substitution pattern estimation (ML); tree to use = automatic (neighbor‐joining tree); statistical method = maximum likelihood; substitution type = nucleotide; model/method = Tamura 3‐parameter model; rates among sites = Gamma distributed (G); no. of discrete Gamma categories = 5; gaps/missing data treatment = partial deletion; site coverage cutoff = 95%; branch swap filter = very strong. The tRNA gene sequences were then subjected to a disparity index test of evolutionary pattern heterogeneity to determine whether the nucleotide substitutions were homogeneous or not (Kumar & Gadagkar, 2001). The disparity index analysis employed the following parameters: analysis = disparity index test of substitution pattern homogeneity; scope = in sequence pairs; no. of Monte Carlo replications = 1,000; substitution type = nucleotide; gaps/missing data treatment = partial deletion; site coverage cutoff = 95%.

### 2.7 Intron analysis

We downloaded the chloroplast genomes of *Nicotiana tabacum* L. (Z00044) and *Bryopsis plumosa* Huds. Ag. (LN810504), and the cyanelle genome of *Cyanophora paradoxa* Korshikov (NC_001675) in order to analyze the evolution of tRNA introns. In previous studies, the introns of tRNAs were found in cyanobacteria genomes, which are less well known (Bonen & Vogel, 2001; Kuhsel et al., 1990; Paquin et al., 1997). Therefore, in this study, the tRNA sequences from *Nostoc* sp. PCC 7524 (gene id: 2509813156), *Gloeocapsa* sp. PCC 73106 (gene id: 2508643885), and *Nostoc* sp. PCC 7107 (gene id: 2503742551) were obtained from a previous study (Mohanta et al., 2017) for further analysis. These sequences were used to extract the introns of tRNA^Leu^ UAA. Finally, the type I intron of the cp tRNA^Leu^ of *V. utile* (*V. utile*_48877), as well as the type I introns from cyanobacteria and the other species mentioned above were subjected to phylogenetic analysis using MAFFT v 7.017 and MEGA 7.0 (Katoh et al., 2002; Kumar et al., 2016). We performed ML analysis in MEGA 7.0 after first conducting model testing. The results indicated that the Kimura 2‐parameter model was suitable for ML analysis. The following parameters were used in MEGA 7.0: analysis = phylogeny reconstruction; statistical model = maximum likelihood; test of phylogeny = bootstrap method; no. of bootstrap replicates = 1,000; substitution type = nucleotides; model/method = Kimura 2‐parameter model; rates among sites = Gamma distributed with invariant sites (G + I); no. of discrete Gamma categories = 5; gaps/missing data treatment = partial deletion; site coverage cutoff = 95%; branch swap filter = very strong. *V. utile* was selected because it is at the base of the phylogenic tree for Adoxaceae species.

## 3 RESULTS

### 3.1 Characteristics of chloroplast tRNAs in Adoxaceae

Chloroplast genomes for the ten Adoxaceae species including *A. moschatellina* (KX258652), *S. corydalifolia* (KX258651), *T. omeiensis* (KX258653), *S. williamsii* (KX510276), *V. utile* (KX792264), *V. betulifolium* (MG738665), *V. japonicum* (MH036493), *V. erosum* (MN641480), *V. carlesii* × *V. macrocephalum* (MN985820), and *V. rhytidophyllum* (MT374829) were downloaded from the NCBI database and subjected to tRNA analysis. The chloroplast genomes of these species each contain 37 tRNA genes. The anticodon compositions and relative positions of these 37 tRNA genes are consistent in each chloroplast genome (Figure S1). Furthermore, the tRNA gene sequences from different species of *Viburnum* are highly consistent, where only several nucleotide changes were found that did not affect the tRNA structure (Table S1). Therefore, *V. utile* was selected as representative of *Viburnum* to compare with species from the other four genera in Adoxaceae. Among the 37 cp tRNA genes, 19 are in the LSC, 14 in the IRs, and one in the SSC. The lengths of the tRNA genes range from 70 nt (tRNA^Gly^ UCC) to 93 nt (tRNA^Ser^ UGA). In general, tRNA genes with the same anticodon composition share the same length, but there are some exceptions. For instance, the nucleotide sequence of chloroplast tRNA^Val^ UAC is typically 75 nt long, but it is 76 nt long in *V. utile* (*V. utile*_53289). Similarly, the nucleotide sequence of chloroplast tRNA^Thr^ GGU is usually 72 nt long, but it is 70 nt long in *T. omeiensis* (*T. omeiensis*_32640). These two exceptions are due to single nucleotide substitutions (Table S1). In general, the average length of the chloroplast tRNA sequences in Adoxaceae is 76 nt, and most of them measure 72 nt (44) or 74 nt (45). However, the nucleotide sequences of tRNA^Gly^ (70–71 nt) and tRNA^Cys^ (71 nt) are relatively short, whereas those of tRNA^Leu^ (80 or 87 nt), tRNA^Tyr^ (84 nt), and tRNA^Ser^ (87–93 nt) are relatively long. In addition, no tRNA encoding UGA (translating selenocysteine) or termination codon was found in the cpDNAs in Adoxaceae. tRNA^Leu^ and tRNA^Ile^ are the most frequent (four), followed by tRNA^Arg^ and tRNA^Ser^ (three). It should be noted that tRNA^Leu^, tRNA^Ile^, and tRNA^Arg^ are found in IRs.

**FIGURE 1:**
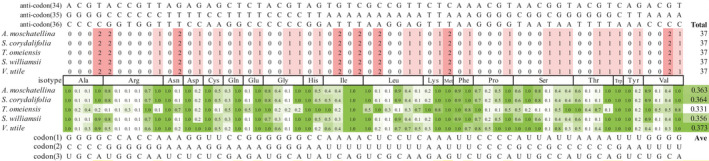
Distributions of tRNA isotypes, anticodons, and CAI‐w values in the chloroplast genomes of Adoxaceae species. The CAI‐w value and the number of anticodons in individual species were converted into different green and red depths. The codon usage bias was measured based on the CAI. It should be noted tRNA^Ile^ is also encoded by CAU in the species considered in this study

The chloroplast tRNAs of Adoxaceae were found to encode 28 anticodons and 61 sense codons, including tRNA^Phe^ GAA, tRNA^Ser^ GGA, tRNA^Tyr^ GUA, tRNA^Cys^ GCA, tRNA^Leu^ UAA, tRNA^Ser^ UGA, tRNA^Leu^ CAA, tRNA^Trp^ CCA, tRNA^Arg^ ACG, tRNA^His^ GUG, tRNA^Leu^ UAG, tRNA^Pro^ UGG, tRNA^Gln^ UUG, tRNA^Ile^ GAU, tRNA^Thr^ GGU, tRNA^Asn^ GUU, tRNA^Ser^ GCU, tRNA^Thr^ UGU, tRNA^Lys^ UUU, tRNA^Arg^ UCU, tRNA^Met^ CAU, tRNA^Ile^ CAU, tRNA^Val^ GAC, tRNA^Asp^ GUC, tRNA^Gly^ GCC, tRNA^Ala^ UGC, tRNA^Val^ UAC, tRNA^Glu^ UUC, and tRNA^Gly^ UCC (Figure 1). In particular, CAU is encoded by both tRNA^Met^ and tRNA^Ile^.

**FIGURE 2:**
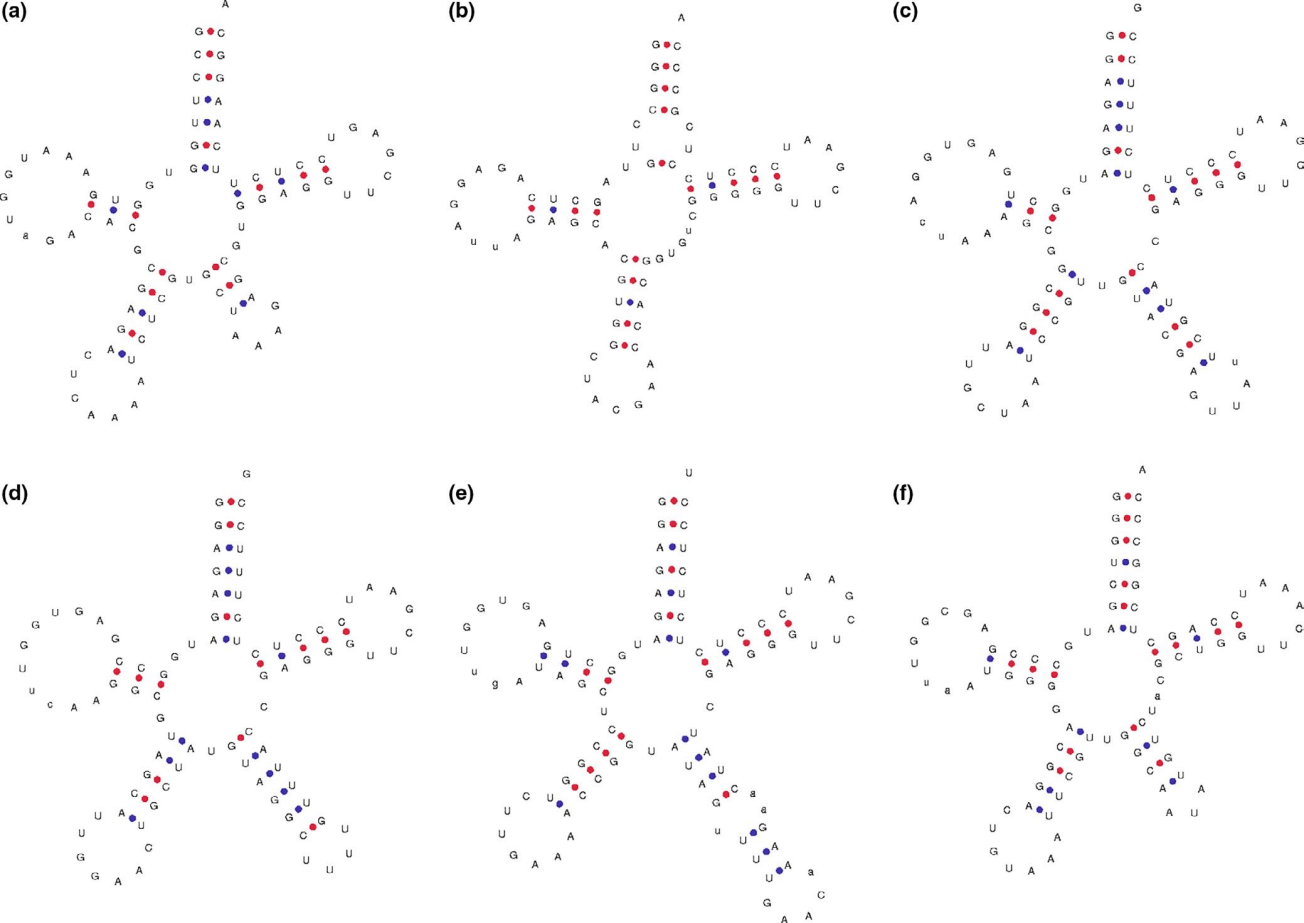
Putative structures of novel chloroplast tRNAs with new stems in the variable region: (a) tRNA^Leu^ CAA, (b) tRNA^Arg^ ACG, (c) tRNA^Ser^ GCU, (d) tRNA^Ser^ GGA, (e) tRNA^Ser^ UGA, and (f) tRNA^Tyr^ GUA

**FIGURE 3:**
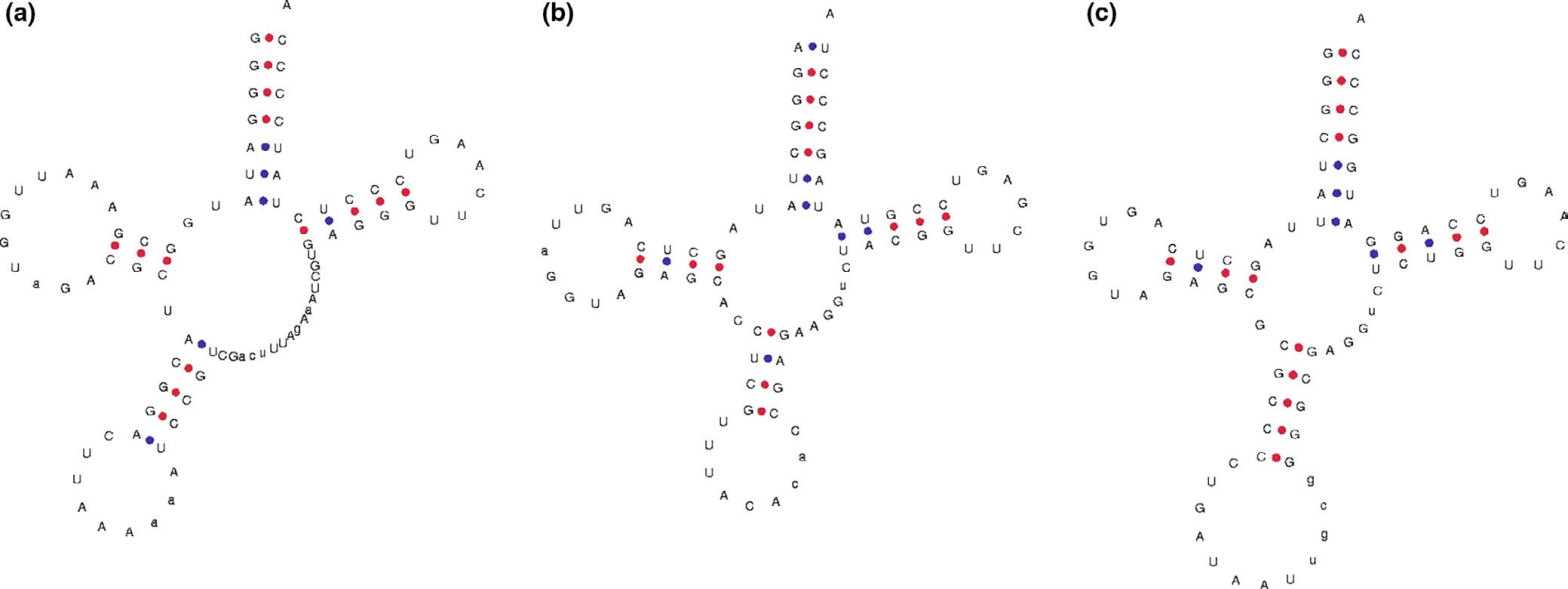
Putative structures of novel chloroplast tRNAs with long anticodon loops: (a) tRNA^Leu^ UAA, (b) tRNA^Val^ UAC, and (c) tRNA^Ile^ GAU

In the present study, 11 putative novel tRNAs were found that differ from the canonical clover leaf‐like structure of tRNA. For instance, a new loop measuring 7 nt long is found at the base of the acceptor arm in tRNA^Arg^ ACG (Figure 2b); one arm and one loop are found in the variable regions in tRNA^Leu^ CAA (Figure 2a), tRNA^Tyr^ GUA (Figure 2f), tRNA^Ser^ GCU, and tRNA^Ser^ GGA, which have not been observed previously (Figure 2c,d); and one new arm and two new loops are found in the variable region of tRNA^Ser^ UGA (Figure 2e). In particular, the loops found in the variable regions of tRNA^Leu^ CAA and tRNA^Tyr^ GUA have similar nucleotide sequences to their anticodon loops. Furthermore, we found that the anticodon loops are particularly long, ranging from 9 to 12 nt, in tRNA^Leu^ UAA, tRNA^Val^ UAC, and tRNA^Ile^ GAU (Figure 3a–c).

### 3.2 Conservation of tRNA sequences

The length of each tRNA part is relatively conserved. In particular, the lengths of the Ψ‐arm and Ψ‐loop are identical in all of the tRNAs that we investigated. Among the 185 chloroplast tRNAs, most (175, 94.59%) have a 7 bp long acceptor arm, 126 (68.11%) have a 4 bp long D‐arm, and 166 (89.73%) have a 5 bp long anticodon arm and a 7 nt long anticodon loop. The length of the D‐loop is relatively variable, where 46 (24.86%) of the tRNAs have a 7 nt long D‐loop, 34 (18.38%) have an 8 nt long D‐loop, 56 (30.27%) have a 9 nt long D‐loop, 20 (10.81%) have a 10 nt long D‐loop, and 31 (16.76%) have an 11 nt long D‐loop. The variable stem is the most variable part of the tRNAs, where 110 of the 185 tRNAs that we investigated have variable stems (59.46%) measure 5 nt. The variable stems over 10 nt in length usually have additional novel arms and loops within them, as discussed in the following. In conclusion, the acceptor arm comprises 5–7 base pairs (bp), the D‐arm is 2–4 bp, the D‐loop is 7–11 nt, the anticodon arm is 4–5 bp, the anticodon loop is 7–12 nt, the Ψ‐arm is 5 bp, the Ψ‐loop is 7 nt, and the variable stem is 4–24 nt. More detailed data are presented in Table S1.

A highly conserved consensus sequence comprising U‐U‐C‐A/G‐A‐x‐U was found in the Ψ‐loop after multiple sequence alignment using all 185 tRNA genes (Table 1). Most of the tRNAs possess a G‐C nucleotide base pair at the basal first position on the D‐arm, but the same position in tRNA^Met^ CAU or tRNA^Tyr^ GUA is occupied by an A‐U base pair instead. Similar to the D‐loop, most of the tRNAs possess an A nucleotide in the first positions at the 5′ end and 3′ end of the D‐loop, but tRNA^Cys^ GCA, tRNA^Ile^ CAU, tRNA^Ser^ GGA, and tRNA^Ser^ GCU possess a G at the 5′ end of the D‐loop, tRNA^Gly^ GCC possesses a C at the 5′ end, and tRNA^Met^ CAU possesses a G at the 3′ end (Table 1). Moreover, most of the tRNAs possess a C or a U nucleotide in the 1st position at the 5′ end of the anticodon loop, but tRNA^Thr^ GGU has an A instead. The 2nd position at the 5′ end of the anticodon loop is a U nucleotide in every tRNA without exception (Table 1). Similarly, most of the tRNAs possess an A or U nucleotide in the 1st position at the 3′ end of the anticodon loop, except tRNA^Met^ CAU and tRNA^Ile^ GAU have a G instead, and tRNA^Val^ UAC and tRNA^Ser^ GGA have a C. However, an A nucleotide is always present in the 2nd position at the 3′ end of the anticodon loop of each tRNA (Table 1). Furthermore, most of the tRNAs possess a G‐C nucleotide base pair at the basal 1st position in the Ψ‐arm, whereas tRNA^Glu^ UUC possesses a U‐A nucleotide base pair at this position. However, no significant consensus sequence is conserved within the acceptor arm and variable stem (Table 1). Thus, tRNA^Lys^ UUU, tRNA^Tyr^ GUA, tRNA^Leu^ UAA, tRNA^Ile^ GAU, tRNA^Ala^ UGC, and tRNA^Arg^ ACG possess a CCA nucleotide sequence at the 3′ end, whereas other tRNAs do not share this characteristic (Table S1).

**TABLE 1:**
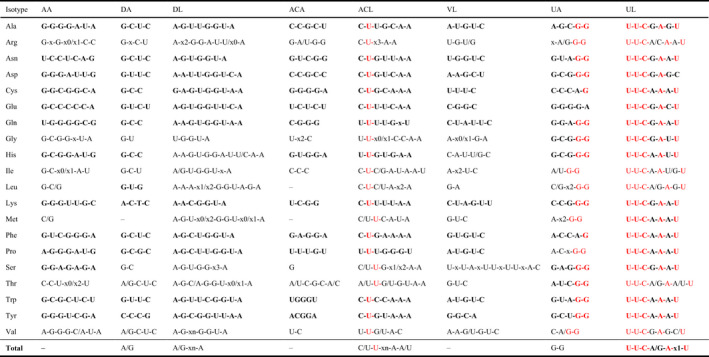
Conserved consensus sequences in chloroplast tRNA genes grouped according to 20 isotypes

Note

The characters in red are the high consensus single nucleotides. The characters in bold are the high consensus polynucleotide sequences in a single structure or isotype. “High” denotes appearance in more than 80% individuals.

### 3.3 Introns in tRNAs

As mentioned above, eight chloroplast tRNA genes are interrupted by introns in each Adoxaceae species. These chloroplast tRNAs comprise tRNA^Leu^ UAA, tRNA^Gly^ UCC, tRNA^Lys^ UUU, tRNA^Val^ UAC, tRNA^Ala^ UGC (two), and tRNA^Ile^ GAU (two), with introns measured 502, 692, 2,521, 575, 815, 815, 940, and 940 nt in length, respectively. In particular, tRNA^Ala^ and tRNA^Ile^ are located in IRs, and the others are in the LSC. The intron in tRNA^Lys^ contains a *matK* gene. The intron in tRNA^Gly^ interrupts the D‐arm of the gene, whereas those in other tRNAs interrupt the anticodon loop. The introns in tRNA^Leu^ are all type I introns, and the introns in the other tRNA isotypes are type II introns. In addition, the consensus sequence “G‐A‐A‐C‐T‐A‐C‐G‐A‐G‐A‐T‐C‐A‐C‐C‐C‐C” is found in the introns in tRNA^Ala^ and tRNA^Ile^.

**FIGURE 4:**
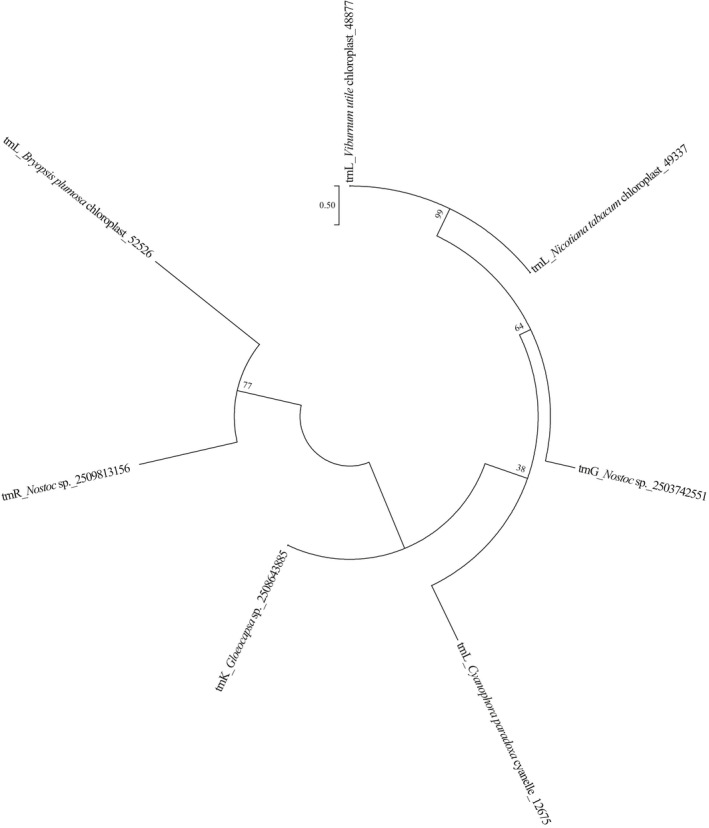
Phylogenetic tree constructed based on type I introns in tRNA genes from various organisms. ML bootstrap values are given adjacent to nodes

A phylogenetic tree was constructed using the seven type I introns in tRNAs from the *V. utile* chloroplast, *N. tabacum* chloroplast, *B. plumosa* chloroplast, *C. paradoxa* cyanelle, as well as the sequences in *Nostoc* sp. PCC 7524, *Gloeocapsa* sp. PCC 73106, and *Nostoc* sp. PCC 7107. The tree indicated that the type I introns in the plastids of *V. utile*, *B. plumosa*, and *C. paradoxa* segregated from different cyanobacterial ancestors (Figure 4). The type I introns in the cp tRNA^Leu^ from *V. utile* and *N. tabacum* have a close relationship with that in the cp tRNA^Gly^ from *Nostoc* sp. PCC 7524 (Figure 4).

**FIGURE 5:**
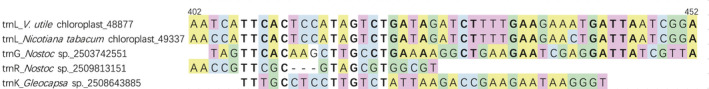
Multiple sequence alignment of type I introns in tRNA genes

Multiple sequence alignment found two consensus sequences “U‐U‐x2‐C” and “U‐x‐G‐x2‐T” in the type I introns in the three selected cyanobacterial species and the two angiosperm species. In addition, the consensus sequence “U‐U‐C‐A‐C‐x4‐U‐x‐G‐x‐C‐T‐G‐A‐x‐A‐x3‐C‐T‐x3‐G‐A‐A‐x5‐G‐A‐T‐T‐A‐x5‐A” was found in the type I introns in *V. utile*, *N. tabacum*, and *Nostoc* sp. PCC 7524 (Figure 5).

### 3.4 Phylogeny and evolution

**FIGURE 6:**
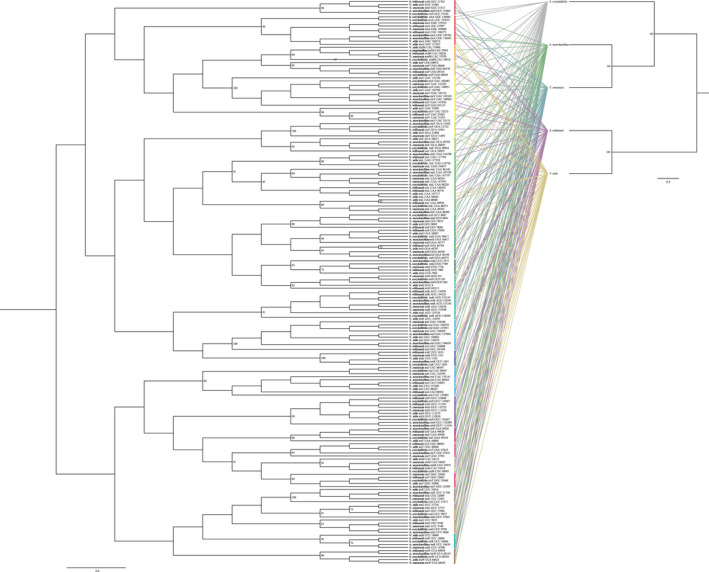
Gene tree based on 185 chloroplast tRNA genes and species tree based on chloroplast genomes (being removed of tRNA sequences). ML bootstrap values ≥65% are given adjacent to nodes. Bars next to the tRNA gene tree are colored based on different tRNA isotypes. Grayish lines connecting two trees show the positions of tRNA on species tree

A phylogenetic tree was constructed based on the 185 chloroplast tRNA genes in the five Adoxaceae species. As shown in Figure 6, the gene tree contains two major clusters and 24 groups. Cluster I consists of 14 groups: tRNA^Val^, tRNA^Ala^, tRNA^Asp^, tRNA^Leu^, tRNA^Ser^, tRNA^fMet^, tRNA^Pro^, tRNA^Ile^, tRNA^Lys^, tRNA^Arg^, tRNA^His^, tRNA^Gln^, tRNA^Cys^, and tRNA^Tyr^. Cluster II consists of 10 groups: tRNA^Asn^, tRNA^Ile^, tRNA^Trp^, tRNA^Arg^, tRNA^Glu^, tRNA^Thr^, tRNA^Met^, tRNA^Gly^, and tRNA^Phe^. In the phylogenetic tree, tRNAs belonging to the same isotype but with different anticodons usually group separately, such as tRNA^Val^ GAC and tRNA^Val^ UAC, tRNA^Thr^ UGU and tRNA^Thr^ GGU, and tRNA^Ser^ UGA, and tRNA^Ser^ GCU and tRNA^Ser^ GGA. In addition, tRNA^Ile^ and tRNA^Arg^ are present in both Cluster I and Cluster II. Intriguingly, tRNA^Ile^, tRNA^Met^, and tRNA^fMet^ share the same anticodon CAU, but their phylogenetic relationships are distant such that tRNA^Ile^ and tRNA^Met^ are in Cluster II, whereas tRNA^fMet^ is in Cluster I next to tRNA^Pro^.

Another two reconciled phylogenetic trees were produced based on a rooted species tree containing seven species and a gene tree containing the 185 cp tRNAs using NOTUNG and GeneRax, respectively. They were constructed to elucidate the evolutionary characteristics of the tRNA genes.

**FIGURE 7:**
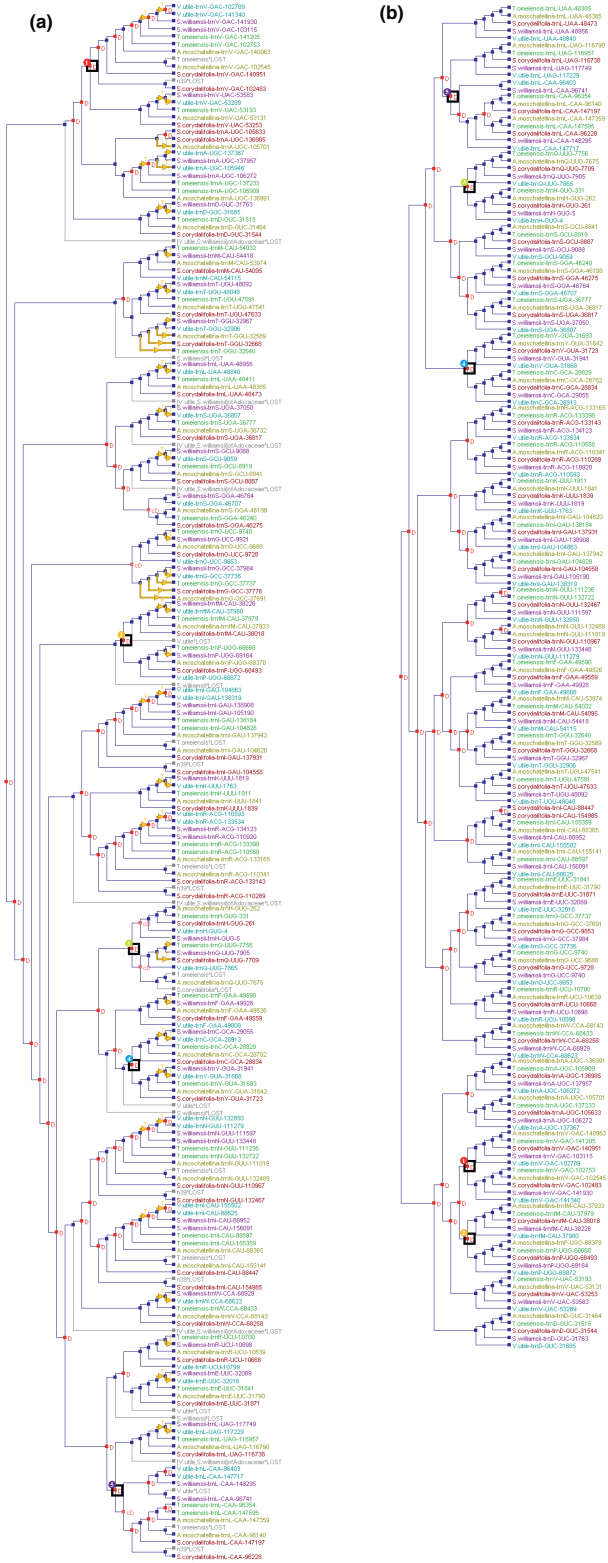
Duplication, loss, and transfer events in the phylogenetic trees based on 185 chloroplast tRNA genes. Colored tip labels represent five various species, the red letter “D” represents duplication events, gray labels represent loss events, yellow arrows represent transfer events, and black squares highlight events shared between the two trees. (a) Inferences from NOTUNG 2.9. (b) Inferences from GeneRax v1.2.2

Our phylogenetic analysis with NOTUNG indicated that during the long evolution of Adoxaceae, the chloroplast tRNA genes underwent events including 60 duplications, five codivergences, 29 losses, and 55 inferred transfers (Figure 7a). Among the gene duplication events, six occurred in *V. utile*, *S. williamsii*, and a putative ancestor designated as “n39,” as well as one in *S. corydalifolia*, seven in *T. omeiensis*, 14 in another putative ancestor designated as “n41,” and nine in more than one Adoxaceae species, whereas no tRNA duplications occurred in *A. moschatellina*. Among the gene loss events, except for tRNA^Gly^ GCC, tRNA^Gly^ UCC, tRNA^Ser^ GGA, and tRNA^His^ GUG, most of the chloroplast tRNA genes underwent loss events during their evolution. Four occurred in *S. williamsii* and *V. utile*, six in “n39,” seven in *T. omeiensis*, and seven in more than one Adoxaceae species, but only one in *S. corydalifolia*. Similar to the duplication events, no tRNA loss events occurred in *A. moschatellina* (Table S2). In addition, during the evolutionary process, the existing tRNA^Ser^ GGA, tRNA^Gly^ GCC, tRNA^Gly^ UCC, tRNA^His^ GUG, tRNA^Gln^ UUG, and tRNA^Leu^ CAA genes underwent codivergence events, and only tRNA^Leu^ CAA and tRNA^His^ GUG underwent no inferred transfer events. However, tRNA^Gly^ GCC and tRNA^Thr^ GGU underwent frequent transfer events during their evolution (four times) (Figure 7). Among the inferred transfer events in species, it is notable that 24 chloroplast tRNA genes were transferred from *T. omeiensis* to *S. williamsii*, and 21 from *S. williamsii* to *V. utile*. Among the loss events, tRNA^Val^ GAC, tRNA^Ile^ GAU, tRNA^Arg^ ACG, tRNA^Aln^ GUU, tRNA^Ile^ UAC, and tRNA^Leu^ CAA underwent loss events in *T. omeiensis* or “n39”; tRNA^Gln^ UUG in *T. omeiensis* or *S. corydalifolia*; tRNA^Val^ GAC, tRNA^Ala^ UGC, tRNA^Val^ UAC, tRNA^Leu^ UAA, tRNA^Ser^ GCU, tRNA^Ser^ UGA, tRNA^Ile^ GAU, tRNA^Lys^ UUU, tRNA^Arg^ ACG, tRNA^Ile^ CAU, tRNA^Asn^ GUU, tRNA^Trp^ CCA, tRNA^Leu^ UAG, tRNA^Cys^ GCA, tRNA^Tyr^ GUA, tRNA^Glu^ UUC, and tRNA^Asp^ GUC in *S. williamsii* or *V. utile*; tRNA^Thr^ UGU, tRNA^Thr^ GGU, tRNA^Met^ CAU, tRNA^Pro^ UGG, tRNA^Phe^ GAA, and tRNA^Arg^ UCU in *S. williamsii*; and tRNA^fMet^ CAU and tRNA^Leu^ CAA in *V. utile* (see Table S2).

Another reconciled phylogenetic tree built using GeneRax indicated that during the long evolution of Adoxaceae, the chloroplast tRNA genes underwent events comprising 144 speciation event, four speciation + loss events, and 40 duplications, but no transfer or loss events (Figure 7b). GeneRax found an average of 0.124 duplications and 0.015 losses for each gene. The chloroplast tRNA genes of the five Adoxaceae species underwent 37 speciation events. Among the duplication events, one each occurred in *A. moschatellina*, *S. corydalifolia*, and *T. omeiensis*. In addition, 36 duplication events occurred in a putative ancestor designated as “n3.” Moreover, the four loss events all occurred in putative ancestors (see Table S2).

### 3.5 Differences in codon usage

The CAI and CAI‐w values were calculated using CODONW 1.4.2 in order to measure the codon usage bias for the chloroplast genomes of the five selected species. The CAI‐w values were similar in different species, except for *T. omeiensis*. The relative adaptiveness values for anticodons GCA, CGU, AGA, AAC, GGA, UUG, CUC, UCU, UCG, UCA, ACC, and ACA in *T. omeiensis* were significantly different from those of tRNAs in other species (difference value ≥ 0.5) (Figure 1). The average CAI‐w values for the other four species were consistent, ranging from 0.356 (*S. williamsii*) to 0.373 (*V. utile*), but the average CAI‐w value (0.331) was relatively low for *T. omeiensis* (Figure 1).

### 3.6 Nucleotide substitutions

A nucleotide substitution from a purine to another purine or from a pyrimidine to another pyrimidine is called a “transition,” whereas a substitution from a purine to a pyrimidine or vice versa is called a “transversion.” In the present study, we found that the average transition rate (8.33%) and average transversion rate (8.34%) were almost equal for tRNA^Ala^, tRNA^Asn^, tRNA^Asp^, tRNA^Cys^, tRNA^Glu^, tRNA^Lys^, tRNA^Trp^, and tRNA^Tyr^ (Table 2). However, the average transition rate (25.00%) and average transversion rate (0%) differed greatly for tRNA^Gln^, tRNA^Phe^, and tRNA^Pro^ (Table 2). In addition, except for tRNA^Ser^ with an average transition rate of 2.45% and average transversion rate of 11.28%, the average transition rates were higher than the average transversion rates for tRNA^Thr^ (8.75%/8.13%), tRNA^Ile^ (12.60%/6.21%), tRNA^Leu^ (13.61%/5.70%), tRNA^Met^ (23.81%/0.60%), tRNA^Arg^ (16.16%/4.42%), tRNA^Val^ (16.35%/4.33%), tRNA^His^ (16.82%/4.10%), and tRNA^Gly^ (17.39%/3.81%) (Table 2). Furthermore, our analysis of the nucleotide substitution rates of the 185 chloroplast tRNAs indicated that the average transition rate (15.25%) was significantly higher than the average transversion rate (4.88%).

**TABLE 2:** Average transition rate, average transversion rate, and transition/transversion ratio (TI/TV) for chloroplast tRNA genes in groups or overall

Isotype	Transition rate	Transversion rate	TI/TV
	Average (%)		Ratio
Ala	8.33	8.34	1.00
Arg	16.16	4.42	3.66
Asn	8.33	8.34	1.00
Asp	8.33	8.34	1.00
Cys	8.33	8.34	1.00
Glu	8.33	8.34	1.00
Gln	25.00	0.00	∞
Gly	17.39	3.81	4.57
His	16.82	4.10	4.11
Ile	12.60	6.21	2.03
Leu	13.61	5.70	2.39
Lys	8.33	8.34	1.00
Met	23.81	0.60	40.02
Phe	25.00	0.00	∞
Pro	25.00	0.00	∞
Ser	2.45	11.28	0.22
Thr	8.75	8.13	1.08
Trp	8.33	8.34	1.00
Tyr	8.33	8.34	1.00
Val	16.35	4.33	3.77
Total	15.25	4.88	3.13

The disparity index test for the homogeneity of the substitution pattern showed that the null hypothesis was rejected for tRNA^Arg^ ACG, tRNA^Arg^ UCU, tRNA^Gln^ UUG, tRNA^Glu^ UUC, tRNA^Ile^ GAU, and tRNA^Tyr^ GUA, but the other tRNA genes evolved with the same nucleotide substitution pattern (homogeneity of the evolutionary process).

## 4 DISCUSSION

### 4.1 Adoxaceae chloroplast tRNAs encode 28 anticodons

Some consensus characteristics of the chloroplast tRNAs in different Adoxaceae species suggest the high conservation of tRNA genes (Kirchner & Ignatova, 2015). In general, 61 sense codons specify various amino acids among 64 genetic codons. However, the chloroplast tRNAs in Adoxaceae only encode 28 anticodons (Figure 1) because some wobble is allowed between the first base of the anticodon (N34) and the third base of the codon (N3) for a tRNA in order to decode the synonymous codons (Crick, 1966). In the genome, synonymous codons are employed unevenly for different genes and highly expressed genes prefer to use codons that are assumed to be translated more efficiently and accurately (Gouy & Gautier, 1982; Grantham et al., 1980). This codon selection strategy is related to the tRNA isotype content of the genome, which is species‐specific (Bennetzen & Hall, 1982). However, this is often not the case in chloroplast genomes (Ikemura, 1985), possibly because some rare codons present in genes regulate circadian cycles, thereby slowing the rate of translation to ensure that the extending polypeptides fold accurately (Xu et al., 2013; Zhou et al., 2013). Moreover, it can be attributed to frequent gene transfers from the plastid to the nucleus (Martin et al., 1998). In the chloroplast genomes of Adoxaceae, except for tRNA^Met^ and tRNA^Trp^, which encode only one anticodon, we found that the consistency between the codon bias and tRNA content is relatively high for tRNA^Ala^, tRNA^Gln^, tRNA^Glu^, tRNA^Ile^, tRNA^Lys^, tRNA^Phe^, and tRNA^Val^, but low for the other tRNA isotypes. Moreover, among the five Adoxaceae species, the average CAI‐w value was relatively low for *T. omeiensis* (0.331). This may indicate a different evolutionary pattern with respect to translation in the *T. omeiensis* chloroplast, which requires further investigation (Figure 1).

Interestingly, a CAU anticodon was found to be encoded by tRNA^Met^, tRNA^fMet^, and tRNA^Ile^ in the chloroplast genomes of all five Adoxaceae species, whereas CAU is typically encoded by tRNA^Met^. In addition, tRNA^Ile^ CAU was found in the chloroplast genomes of Caprifoliaceae and monocot species in previous studies (Fan et al., 2018; Mohanta et al., 2019). The tRNA^Ile^ CAU present in the bacterial species *Bacillus subtilis* Cohn was studied in detail. In *B. subtilis*, a tRNA^Ile^ CAU‐lysidine synthetase mutant provides a U34 wobble for tRNA^Ile^ CAU to decode the AUA codon, but the tRNA^Ile^ LAU‐lysidine *TilS* mutant fails to decode the AUA codon, which results in the translation of the former (Köhrer et al., 2014). The translation mechanism in *B. subtilis* may be similar to that in the chloroplasts of Adoxaceae species. Furthermore, it should be noted that tRNA^Ile^ was found to have a considerably high CAI‐w value in the present study (Figure 1). The tRNA^Ile^ encoding CAU is probably an adaptive evolutionary characteristic for increasing the translation efficiency and accuracy. In addition, the function of tRNA^Met^ is to add a methionine to the polypeptide chain, whereas the function of tRNA^fMet^ is to mediate codon initiation (Kozak, 1999; Varshney et al., 1991). The translation product of tRNA^fMet^ is N‐formylmethionine, where the free amino group is modified by a formyl group. N‐formylmethionine is important for the selection of the initiator tRNA, and it improves the efficiency of initiation. In addition, tRNA^Met^ and tRNA^fMet^ are considered to be features of prokaryotic and organellar genomes (Salinas‐Giegé et al., 2015). Therefore, in the present study, the detection of these two tRNAs supports the prokaryotic origin of the chloroplast genome in Adoxaceae species. However, it is not clear how these tRNA are translated into different products (isoleucine, methionine, and N‐formylmethionine) in the chloroplast.

According to our phylogenetic analysis of the 28 groups of tRNA genes, some synonymous tRNA genes are located in different main clusters. For instance, tRNA^Ile^, tRNA^Arg^, and tRNA^Leu^ are present in both Cluster I and Cluster II, thereby demonstrating that synonymous tRNAs probably evolved from multiple ancestors. These findings improve our comprehensive understanding of the origin of the tRNA family.

### 4.2 tRNAs with novel putative structures

Some tRNAs were found that differ from the canonical clover leaf‐like tRNA with novel putative structures, including tRNA^Leu^ CAA, tRNA^Arg^ ACG, tRNA^Ser^ GCU, tRNA^Ser^ GGA, tRNA^Ser^ UGA, tRNA^Tyr^ GUA, tRNA^Leu^ UAA, tRNA^Val^ UAC, and tRNA^Ile^ GAU (Figures 2 and 3). Among these tRNAs, tRNA^Leu^ CAA, tRNA^Ser^ GCU, tRNA^Ser^ GGA, tRNA^Ser^ UGA, and tRNA^Tyr^ GUA have an arm and a loop in the variable region; tRNA^Arg^ ACG has a loop in the acceptor arm; and tRNA^Leu^ UAA, tRNA^Val^ UAC, and tRNA^Ile^ GAU have an anticodon loop comprising 7 nt (Kirchner & Ignatova, 2015). The functions of these novel putative structures require further study.

Some special characteristics were observed in the present study. For instance, some of the novel loops present in the variable regions are similar to the anticodon loop. Intriguingly, in the tRNA^Tyr^ GUA, the novel loop in the variable region has the nucleotide sequence “AUA,” which is identical to the anticodon “AUA” that is typically encoded by tRNA^Tyr^ (Figure 2f), but this characteristic was not found in other tRNAs. We also found that the tRNAs with novel loops in the variable regions, that is, tRNA^Leu^, tRNA^Ser^, and tRNA^Tyr^, have low consistency in terms of their codon bias and tRNA content, which may be related to inefficient translation (Figure 1). However, the “AUA” anticodon “encoded” by the novel loop in tRNA^Tyr^ was found to have a high CAI‐w value (1.0) in this study. Therefore, we hypothesize that this special tRNA structure may be a supplement to the anticodon stem and it could be an adaptive characteristic to promote the efficiency of translation. Functional study of these novel putative structures of cp tRNAs will contribute to our understanding of the translation process in chloroplasts.

Moreover, due to the high conservation of tRNA structures at the genus level in Adoxaceae, for example, in *Viburnum*, as mentioned above, these novel tRNA structures may serve as a framework for detecting biochemical and genomic structural synapomorphies in clades within the family Adoxaceae.

### 4.3 Conserved sequences in tRNAs

Our analysis of the conserved sequences in tRNAs detected a consensus sequence “U‐U‐C‐x‐A‐x‐U” in the Ψ‐loop (Table 1). Most of the tRNAs possess a G nucleotide at the 1st position at the 5′ end of the acceptor arm, whereas a U nucleotide was observed instead in tRNA^Gln^ and tRNA^Asn^ (Table 1). In a previous study, a consensus sequence “U‐U‐C‐x‐A” was found in the Ψ‐loop. In addition, a conserved “U” nucleotide was reported to be present at the 1st position on the acceptor arm in tRNA^Gln^ and tRNA^Asn^ in the nuclear genome (Provan et al., 1999). Furthermore, in most tRNAs, a conserved “C‐U” or “U‐U” nucleotide sequence is found at the 1st and 2nd positions at the 5′ end of the anticodon loop, as shown in a previous study (Sharp et al., 1985).

In a previous study, two conserved sequences comprising ^7^GTGGCNNAGT‐‐‐GGT‐AGNGC‐ (A box) and ^52^GGTTCGANTCC (B box) were found in traditional tRNA genes (“‐” denotes a gap filled with any base, or none at all, and “N” indicates one random nucleotide) (Laslett & Canback, 2004). The A box starts from the back part of the acceptor arm and ends at the fore part of the D‐loop, while the B box contains 2 bp of the Ψ‐arm and the whole Ψ‐loop. A previous study suggested that these two conserved sequences are promoter signal sequences for RNA polymerase III intragenic transcription (Sharp et al., 1985). The sequences of the A and B box found in this study are slightly different from those reported previously. For the A box, a consensus sequence ^7^GUGGCNNAGU‐‐‐GGU‐AGGC was found in tRNA^Pro^ UGG, whereas a consensus sequence ^7^GUGGCNNAGU‐ was found in tRNA^Gln^ UUG (Table 1). For the B box, a consensus sequence ^52^GGUUCNANUCC was found in most of the tRNAs. However, a consensus “G” nucleotide was found at the 4th position in the Ψ‐loop of only 11 tRNA isotypes, whereas the other tRNAs possess an A nucleotide at this position instead (Table 1). However, the nucleotide sequence segment is “A‐G” instead of “G‐G” at the 1st and 2nd positions at the 3′ end of the Ψ‐arm in tRNA^Cys^ and tRNA^Phe^, and the nucleotide at the 3′ end of the Ψ‐loop is “C” instead of “U” in tRNA^Asp^ (Table 1). The high coherence of the B box sequences indicates the essential biological function of the Ψ‐stem, but the variation is difficult to understand. In the present study, we predicted the structures of tRNAs based on the chloroplast DNA sequences. Therefore, it is possible that an unknown enzyme catalyzes a post‐transcriptional change in the sequence from “A‐G” or “G‐A” to “G‐G” during the biochemical process to ensure the conservation of the tRNA structure. This process may be similar to the nucleotide substitution from “G” to “Q” catalyzed by the guanine insertion enzyme (Farkas & Singh, 1973).

In living cells, a CCA sequence at the 3′ end is required for tRNAs to accept amino acids. tRNA nucleotidyltransferase, which is also called CCA‐adding enzyme, can attach an additional CCA tail to the 3′ end of a tRNA. However, according to a previous report, chloroplast genomes cannot express this enzyme, but they can encode a CCA nucleotide sequence at the 3′ end of the original tRNA gene instead (Mohanta et al., 2019). In the present study, a C‐C‐A tail was found in tRNA^Lys^, tRNA^Tyr^, tRNA^Leu^, tRNA^Ile^, tRNA^Ala^, and tRNA^Arg^. However, it was absent from the other tRNAs we investigated in this study (Table S1).

The conserved segments that we found might be related to some vital functions in the biochemical processes in chloroplast, which require further study.

### 4.4 Type I introns reflect the cyanobacterial origin of chloroplasts

The tRNAs with introns investigated in the present study include tRNA^Leu^, tRNA^Gly^, tRNA^Lys^, tRNA^Val^, tRNA^Ala^, and tRNA^Ile^. The introns in tRNA^Leu^ are type I introns, whereas the others are type II introns. The type I intron is usually found in tRNA^Leu^ UAA in organelle and cyanobacterial genomes, but it is rarely found in other chloroplast tRNAs. However, the type I intron is common in tRNAs in cyanobacterial genomes (Bonen & Vogel, 2001; Kuhsel et al., 1990; Paquin et al., 1997). A recent study reported the presence of type I introns in cyanobacterial tRNA^Arg^, tRNA^Gly^, and tRNA^Lys^ (Mohanta et al., 2017). In the present study, we conducted phylogenetic analyses of the type I introns within the cyanobacterial and organelle genomes of angiosperms, green algae, and *C. paradoxa*. We found that the type I introns in chloroplast tRNA^Leu^ in angiosperms (*V. utile* and *N. tabacum*) have a close relationship with the type I introns in tRNA^Gly^ in *Nostoc* sp. PCC 7524 (Figure 4). This result suggests that angiosperm chloroplasts evolved from a common cyanobacterial lineage, as suggested in a recent study of monocot plants (Mohanta et al., 2019). Furthermore, the type I introns in angiosperms, green algae, and *C. paradoxa* do not form a monophyletic group in the phylogenetic tree (Figure 4). This result indicates that the organelles of Spermatophyta (represented by *Viburnum utile* and *Nicotiana tabacum*), Glaucophyta (represented by *Cyanophora*), and Chlorophyta (represented by Bryopsis) evolved from different cyanobacterial lineages, in agreement with previous studies of ancient tRNA^Leu^ introns (Kuhsel et al., 1990; Paquin et al., 1997). Moreover, although the nucleotide sequences of type I introns are not highly conserved (Bonen & Vogel, 2001), two consensus sequences comprising “U‐U‐x2‐C” and “U‐x‐G‐x2‐T” were identified in the type I introns investigated in the present study, thereby demonstrating the close evolutionary relationships between organelles and cyanobacteria (Figure 5). This study of type I introns provides insights into the phylogenetic relationships among cyanobacteria and various eukaryote organelles.

### 4.5 Duplication events dominated during the evolution of cp tRNAs in Adoxaceae

During the evolutionary process, species gain new genes mainly by gene duplication and retention, which contribute greatly to genetic diversity and lead to the emergence of new gene functions (Magadum et al., 2013; Panchy et al., 2016). According to previous studies, gene duplication may be caused by genome duplication, retrotransposons, and unequal crossing over (Ohta, 2000).

The chloroplast genomes of land plants are relatively highly conserved (Kim et al., 2009; Millen et al., 2001; Palmer, 1983), but in this study, gene duplication events were found to have dominated the evolution of chloroplast tRNAs in Adoxaceae, where the agreement was strong using the NOTUNG and GeneRax methods. Forty duplications were detected during the evolution of the chloroplast tRNAs according to the GeneRax analysis results (Figure 7). The translation efficiency mediated by cp tRNAs is important for the survival of photosynthetic land plants, including Adoxaceae species. Thus, in order to improve the translation efficiency, chloroplast tRNAs might have undergone positive selection to multiply their numbers, especially in the early stage of evolution (Hughes, 1994).

According to a recent study, simulations indicated that GeneRax recovered an unbiased estimate of the DTL events (Benoit et al., 2020). In this study, GeneRax reduced the number of DTLs compared with NOTUNG. The results obtained by GeneRax analysis showed that there is no evidence of transfer events in the cp tRNA of Adoxaceae. This result agrees with previous studies of a flowering host plant, *Amborella*, which experienced massive horizontal gene transfers (HGT) in the mitochondrial genome but no HGT in the chloroplast genome (Bergthorsson et al., 2004; Rice et al., 2013). Studies have also shown that the plastid genome is highly resistant to the uptake of intracellular DNA (Lemieux et al., 2000; Palmer, 1990). However, HGT to plastid genomes was detected in flowering plants and dinoflagellates in some recent studies (Moszczynski et al., 2012; Rice & Palmer, 2006; Wang et al., 2019). Considering the rejection of HGT due to long‐branch attraction and other potential errors (Rice & Palmer, 2006), the HGT detected by NOTUNG in the present study cannot be completely rejected, but stronger evidence is required.

Some previous studies have suggested possible causes of DTL events. For instance, gene duplications or losses might have been caused by the contraction and expansion of IRs (Wang & Messing, 2011), or by HGT from chloroplasts to the nucleus (Bennetzen & Hall, 1982; Manen et al., 2010). In addition, gene transfers might have been caused by plastid capture (capture of the chloroplast genome from a recipient species by a donor species) (Rieseberg, 1995). However, both programs cannot be set to ignore duplications and losses in the cpDNA genome, and thus, the biological background of these events is not clear. We consider that additional analyses of various other gene families on chloroplast genomes may provide a comprehensive view of the dynamics of Adoxaceae chloroplast genome evolution to further understand the specific evolutionary process for cp tRNAs.

In conclusion, the modern chloroplast tRNA pattern can be attributed mainly to tRNA duplication, as well as several loss events. Our results demonstrate that even at the family level, cp tRNA genes experienced multiple events during their evolution, and comparisons between Adoxaceae and other families will provide us with more information regarding cp tRNA evolution.

### 4.6 Transitions are the most frequent nucleotide substitutions

The molecular clock hypothesis assumes that the rate of molecular evolution has been constant over time and that molecules can act as indicators of the evolutionary pattern (Zuckerkandl, 1962). Therefore, using phylogenetic analysis to compare the changes in molecular sequences can determine the time of evolutionary divergence. The DNA substitution model hypothesis also assumes that the nucleotide substitution rate is equal (Holmquist et al., 1972). However, due to purifying selection, recent studies have indicated that nonsynonymous nucleotide substitutions have occurred less frequently than synonymous substitutions and that some nucleotide substitutions in the chloroplast genome might have been eliminated by the DNA repair mechanism (Ivanova et al., 2017). In the present study, we investigated the ratio of transitions and transversions, and found an uneven pattern of nucleotide substitutions for the cp tRNAs in Adoxaceae species. Our results showed that transitions (15.25%) are more frequent than transversions (4.88%), as shown in similar studies (Mohanta et al., 2019; Purvis & Bromham, 1997). Our results showed that the transition/transversion bias for cp tRNA in Adoxaceae is 3.13, which is slightly higher than that for the cp tRNA in monocot species (2.86) but significantly higher than the expected random value (2) (Holmquist, 1983; Mohanta et al., 2019). Thus, despite the conservation of chloroplast tRNA genes, the rate of single nucleotide substitutions is not identical for cp tRNAs from different angiosperm taxa. In addition, the nucleotide substitution pattern is tRNA specific. The average rates of transitions and transversions are almost equal for tRNA^Ala^, tRNA^Asn^, tRNA^Asp^, tRNA^Cys^, tRNA^Glu^, tRNA^Lys^, tRNA^Thr^, tRNA^Trp^, and tRNA^Tyr^. However, for tRNA^Gln^, tRNA^Phe^ and tRNA^Pro^, the frequency of transitions is considerably higher and the transversion rate is close to zero. Moreover, for tRNA^Ile^, tRNA^Leu^, tRNA^Met^, tRNA^Arg^, tRNA^Val^, tRNA^His^, and tRNA^Gly^, the transition rate exceeds the transversion rate, but the opposite is found for tRNA^Ser^ (Table 2).

## CONFLICT OF INTEREST

The authors have no conflicts of interest to declare.

## AUTHOR CONTRIBUTIONS

**Qiu‐Yi Zhong:** Resources (equal); Software (equal); Writing‐original draft (equal). **Xiao‐Gang Fu:** Methodology (equal); Resources (equal). **Ting‐Ting Zhang:** Resources (equal); Software (equal); Validation (equal). **Tong Zhou:** Investigation (equal); Resources (equal). **Ming Yue:** Resources (equal); Software (equal). **Jian‐Ni Liu:** Supervision (equal); Writing‐review & editing (equal). **Zhonghu Li:** Supervision (equal); Visualization (equal); Writing‐original draft (equal); Writing‐review & editing (equal).

## Supporting information

Fig S1

Table S1

Table S2

## Data Availability

DNA sequences: GenBank accession numbers NCBI: KX258651–KX258653, KX510276 and KX792264.

## References

[ece37133-bib-0001] Angiosperm Phylogeny Group (2009). An update of the Angiosperm Phylogeny Group classification for the orders and families of flowering plants: APG III. Botanical Journal of the Linnean Society, 161(2), 105–121.

[ece37133-bib-0002] Bennetzen, J. L. , & Hall, B. D. (1982). Codon selection in yeast. Journal of Biological Chemistry, 257(6), 3026–3031. 7037777

[ece37133-bib-0003] Benoit, M. , Kozlov, A. M. , Alexandros, S. , & GeneRax, S. G. J. (2020). A tool for species tree‐aware maximum likelihood based gene family tree inference under gene duplication, transfer, and loss. Molecular Biology and Evolution, 37(9), 2763–2774. 10.1093/molbev/msaa141 32502238 PMC8312565

[ece37133-bib-0004] Bergthorsson, U. , Richardson, A. O. , Young, G. J. , Goertzen, L. R. , & Palmer, J. D. (2004). Massive horizontal transfer of mitochondrial genes from diverse land plant donors to the basal angiosperm *Amborella* . Proceedings of the National Academy of Sciences of the United States of America, 101(51), 17747–17752. 10.1073/pnas.0408336102 15598737 PMC539785

[ece37133-bib-0005] Blee, E. , & Joyard, J. (1996). Envelope membranes from spinach chloroplasts are a site of metabolism of fatty acid hydroperoxides. Plant Physiology, 110(2), 445–454. 10.1104/pp.110.2.445 12226196 PMC157739

[ece37133-bib-0006] Bonen, L. , & Vogel, J. (2001). The ins and outs of group II introns. Trends in Genetics, 17(6), 322–331. 10.1016/S0168-9525(01)02324-1 11377794

[ece37133-bib-0007] Chan, P. P. , Cozen, A. E. , & Lowe, T. M. (2011). Discovery of permuted and recently split transfer RNAs in Archaea. Genome Biology, 12(4), R38 10.1186/gb-2011-12-4-r38 21489296 PMC3218864

[ece37133-bib-0008] Chen, K. , Durand, D. , & Farach‐Colton, M. (2000). Notung: A program for dating gene duplications and optimizing gene family trees. Journal of Computational Biology, 7(3/4), 429–447. 10.1089/106652700750050871 11108472

[ece37133-bib-0009] Crick, F. H. (1966). Codon‐anticodon pairing: The wobble hypothesis. Journal of Molecular Biology, 19(2), 548–555. 10.1016/S0022-2836(66)80022-0 5969078

[ece37133-bib-0010] Daniell, H. , Lin, C. S. , Yu, M. , & Chang, W. J. (2016). Chloroplast genomes: Diversity, evolution, and applications in genetic engineering. Genome Biology, 17(1), 134 10.1186/s13059-016-1004-2 27339192 PMC4918201

[ece37133-bib-0011] Delannoy, E. , Le Ret, M. , Faivre‐Nitschke, E. , Estavillo, G. M. , Bergdoll, M. , Taylor, N. L. , Pogson, B. J. , Small, I. , Imbault, P. , & Gualberto, J. M. Arabidopsis tRNA adenosine deaminase arginine edits the wobble nucleotide of chloroplast tRNAArg (ACG) and is essential for efficient chloroplast translation. The Plant Cell, 21(7), 2058–2071. 10.1105/tpc.109.066654 PMC2729595 19602623

[ece37133-bib-0012] Donoghue, M. J. , Eriksson, T. , Reeves, P. A. , & Olmstead, R. (2001). Phylogeny and phylogenetic taxonomy of Dipsacales, with special reference to *Sinadoxa* and *Tetradoxa* (Adoxaceae). Harvard Papers in Botany, 6(2), 459–479.

[ece37133-bib-0013] Eriksson, T. , & Donoghue, M. J. (1997). Phylogenetic relationships of *Sambucus* and *Adoxa* (Adoxoideae, Adoxaceae) based on nuclear ribosomal *ITS* sequences and preliminary morphological data. Systematic Botany, 22(3), 555 10.2307/2419828

[ece37133-bib-0014] Fan, W. B. , Wu, Y. , Yang, J. , Shahzad, K. , & Li, Z. H. (2018). Comparative chloroplast genomics of Dipsacales species: Insights into sequence variation, adaptive evolution, and phylogenetic relationships. Frontiers in Plant Science, 9, 689 10.3389/fpls.2018.00689 29875791 PMC5974163

[ece37133-bib-0015] Farkas, W. R. , & Singh, R. D. (1973). Guanylation of transfer ribonucleic acid by a cell‐free lysate of rabbit reticulocytes. Journal of Biological Chemistry, 248(22), 7780–7785. 4750426

[ece37133-bib-0016] Gouy, M. , & Gautier, C. (1982). Codon usage in bacteria: Correlation with gene expressivity. Nucleic Acids Research, 10(22), 7055–7074. 10.1093/nar/10.22.7055 6760125 PMC326988

[ece37133-bib-0017] Grantham, R. , Gautier, C. , Gouy, M. , Mercier, R. , & Pave, A. (1980). Codon catalog usage and the genome hypothesis. Nucleic Acids Research, 8(1), r49–r62. 10.1093/nar/8.1.197-c 6986610 PMC327256

[ece37133-bib-0018] Holley, R. W. , Apgar, J. , Everett, G. A. , Madison, J. T. , Marquisee, M. , Merrill, S. H. , Penswick, J. R. , & Zamir, A. (1965). Structure of a ribonucleic acid. Science, 147(3664), 1462–1465. 10.1126/science.147.3664.1462 14263761

[ece37133-bib-0019] Holmquist, R. (1983). Transitions and transversions in evolutionary descent: An approach to understanding. Journal of Molecular Evolution, 19(5), 391 10.1007/BF02101644 6571218

[ece37133-bib-0020] Holmquist, R. , Cantor, C. , & Jukes, T. (1972). Improved procedures for comparing homologous sequences in molecules of proteins and nucleic acids. Journal of Molecular Biology, 64(1), 145–161. 10.1016/0022-2836(72)90326-9 5015396

[ece37133-bib-0021] Huang, B. , Johansson, M. J. O. , & Byström, A. S. (2005). An early step in wobble uridine tRNA modification requires the elongator complex. RNA, 11(4), 424–436. 10.1261/rna.7247705 15769872 PMC1370732

[ece37133-bib-0022] Hughes, A. L. (1994). The evolution of functionally novel proteins after gene duplication. Proceedings of the Royal Society of London Series B: Biological Sciences, 256(1346), 119–124. 8029240 10.1098/rspb.1994.0058

[ece37133-bib-0023] Ikemura, T. (1985). Codon usage and tRNA content in unicellular and multicellular organisms. Molecular Biology and Evolution, 2(1), 13–34. 3916708 10.1093/oxfordjournals.molbev.a040335

[ece37133-bib-0024] Ivanova, Z. , Sablok, G. , Daskalova, E. , Zahmanova, G. , Apostolova, E. , Yahubyan, G. , & Baev, V. (2017). Chloroplast genome analysis of resurrection tertiary relict *Haberlea rhodopensis* highlights genes important for desiccation stress response. Frontiers in Plant Science, 8, 204 10.3389/fpls.2017.00204 28265281 PMC5316520

[ece37133-bib-0025] Kanai, A. (2015). Disrupted tRNA genes and tRNA fragments: A perspective on tRNA gene evolution. Life, 5(1), 321–331. 10.3390/life5010321 25629271 PMC4390854

[ece37133-bib-0026] Katoh, K. , Misawa, K. , Kuma, K. , & Miyata, T. (2002). MAFFT: A novel method for rapid multiple sequence alignment based on fast Fourier transform. Nucleic Acids Research, 30(14), 3059–3066. 10.1093/nar/gkf436 12136088 PMC135756

[ece37133-bib-0027] Kim, Y. K. , Park, C. W. , & Kim, K. J. (2009). Complete chloroplast DNA sequence from a Korean endemic genus, *Megaleranthis saniculifolia*, and its evolutionary implications. Molecules and Cells, 27(3), 365–381. 10.1007/s10059-009-0047-6 19326085

[ece37133-bib-0028] Kirchner, S. , & Ignatova, Z. (2015). Emerging roles of tRNA in adaptive translation, signalling dynamics and disease. Nature Reviews Genetics, 16(2), 98–112. 10.1038/nrg3861 25534324

[ece37133-bib-0029] Köhrer, C. , Mandal, D. , Gaston, K. W. , Grosjean, H. , Limbach, P. A. , & RajBhandary, U. L. (2014). Life without tRNA^Ile^‐lysidine synthetase: Translation of the isoleucine codon AUA in *Bacillus subtilis* lacking the canonical tRNA2Ile. Nucleic Acids Research, 42(3), 1904–1915. 10.1093/nar/gkt1009 24194599 PMC3919564

[ece37133-bib-0030] Korpelainen, H. (2004). The evolutionary processes of mitochondrial and chloroplast genomes differ from those of nuclear genomes. Die Naturwissenschaften, 91(11), 505–518. 10.1007/s00114-004-0571-3 15452701

[ece37133-bib-0031] Kozak, M. (1999). Initiation of translation in prokaryotes and eukaryotes. Gene, 234(2), 187–208. 10.1016/S0378-1119(99)00210-3 10395892

[ece37133-bib-0032] Kuhsel, M. G. , Strickland, R. , & Palmer, J. D. (1990). An ancient group I intron shared by eubacteria and chloroplasts. Science, 250(4987), 1570–1573. 10.1126/science.2125748 2125748

[ece37133-bib-0033] Kumar, S. , & Gadagkar, S. R. (2001). Disparity index: A simple statistic to measure and test the homogeneity of substitution patterns between molecular sequences. Genetics, 158(3), 1321–1327. 11454778 10.1093/genetics/158.3.1321 PMC1461708

[ece37133-bib-0034] Kumar, S. , Stecher, G. , & Tamura, K. (2016). MEGA7: Molecular evolutionary genetics analysis version 7.0 for bigger datasets. Molecular Biology and Evolution, 33(7), 1870–1874. 10.1093/molbev/msw054 27004904 PMC8210823

[ece37133-bib-0035] Laslett, D. , & Canback, B. (2004). ARAGORN, a program to detect tRNA genes and tmRNA genes in nucleotide sequences. Nucleic Acids Research, 32(1), 11–16. 10.1093/nar/gkh152 14704338 PMC373265

[ece37133-bib-0036] Lemieux, C. , Otis, C. , & Turmel, M. (2000). Ancestral chloroplast genome in *Mesostigma* viride reveals an early branch of green plant evolution. Nature, 403, 649–652. 10688199 10.1038/35001059

[ece37133-bib-0037] Liang, H. X. , & Wu, Z. Y. (1995). On the taxanomic system, phylogeny and distribution in Adoxaceae. Acta Botanica Yunnanica, 17(4), 380–390.

[ece37133-bib-0038] Lowe, T. M. , & Chan, P. P. (2016). tRNAscan‐SE On‐line: Integrating search and context for analysis of transfer RNA genes. Nucleic Acids Research, 44(W1), W54–W57. 10.1093/nar/gkw413 27174935 PMC4987944

[ece37133-bib-0039] Magadum, S. , Banerjee, U. , Murugan, P. , Gangapur, D. , & Ravikesavan, R. (2013). Gene duplication as a major force in evolution. Journal of Genetics, 92(1), 155–161. 10.1007/s12041-013-0212-8 23640422

[ece37133-bib-0040] Manen, J. F. , Barriera, G. , Loizeau, P. A. , & Naciri, Y. (2010). The history of extant *Ilex* species (Aquifoliaceae): Evidence of hybridization within a Miocene radiation. Molecular Phylogenetics and Evolution, 57(3), 961–977. 10.1016/j.ympev.2010.09.006 20870023

[ece37133-bib-0041] Mao, K. S. , Yao, X. L. , & Huang, Z. H. (2005). Molecular phylogeny and species speciation of Adoxaceae.s.s. Acta Botanica Yunnanica, 27(6), 620–628.

[ece37133-bib-0042] Martin, W. , Stoebe, B. , Goremykin, V. , Hapsmann, S. , Hasegawa, M. , & Kowallik, K. V. (1998). Gene transfer to the nucleus and the evolution of chloroplasts. Nature, 393(6681), 162–165. 10.1038/30234 11560168

[ece37133-bib-0043] Millen, R. S. , Olmstead, R. G. , Adams, K. L. , Palmer, J. D. , Lao, N. T. , Heggie, L. , Kavanagh, T. A. , Hibberd, J. M. , Giray, J. C. , Morden, C. W. , Calie, P. J. , Jermiin, L. S. , & Wolfe, K. H. (2001). Many parallel losses of *inf*A from chloroplast DNA during angiosperm evolution with multiple independent transfers to the nucleus. The Plant Cell, 13(3), 645–658. 11251102 10.1105/tpc.13.3.645 PMC135507

[ece37133-bib-0044] Mohanta, T. K. , Khan, A. L. , Hashem, A. , Avd Allah, E. F. , Yadav, D. , & Al‐Harrasi, A. (2019). Genomic and evolutionary aspects of chloroplast tRNA in monocot plants. BMC Plant Biology, 19, 39 10.1186/s12870-018-1625-6 30669974 PMC6341768

[ece37133-bib-0045] Mohanta, T. K. , Syed, A. S. , Ameen, F. , & Bae, H. (2017). Novel genomic and evolutionary perspective of cyanobacterial tRNAs. Frontiers in Genetics, 8, 200 10.3389/fgene.2017.00200 29321793 PMC5733544

[ece37133-bib-0046] Moszczynski, K. , Mackiewicz, P. , & Bodyl, A. (2012). Evidence for horizontal gene transfer from bacteroidetes bacteria to dinoflagellate minicircles. Molecular Biology and Evolution, 29(3), 887–892. 10.1093/molbev/msr276 22075114

[ece37133-bib-0047] Noctor, G. , Arisi, A. M. , Jouanin, L. , & Foyer, C. H. (1998). Manipulation of glutathione and amino acid biosynthesis in the chloroplast. Plant Physiology, 118(2), 471–482. 10.1104/pp.118.2.471 9765532 PMC34822

[ece37133-bib-0048] Ohta, T. (2000). Evolution of gene families. Gene, 259(1), 45–52. 10.1016/S0378-1119(00)00428-5 11163960

[ece37133-bib-0049] Palmer, J. D. (1983). Chloroplast DNA exists in two orientations. Nature, 301(5895), 92–93. 10.1038/301092a0

[ece37133-bib-0050] Palmer, J. D. (1990). Contrasting modes and tempos of genome evolution in land plant organelles. Trends in Genetics, 6, 115–120. 10.1016/0168-9525(90)90125-P 2132730

[ece37133-bib-0051] Panchy, N. , Lehti‐Shiu, M. , & Shiu, S. H. (2016). Evolution of gene duplication in plants. Plant Physiology, 171(4), 2294–2316. 10.1104/pp.16.00523 27288366 PMC4972278

[ece37133-bib-0052] Paquin, B. , Kathe, S. D. , Nierzwicki‐Bauer, S. A. , & Shub, D. A. (1997). Origin and evolution of group I introns in cyanobacterial tRNA genes. Journal of Bacteriology, 179(21), 6798–6806. 10.1128/JB.179.21.6798-6806.1997 9352932 PMC179611

[ece37133-bib-0053] Peden, J. F. (2000). Analysis of codon usage (90(1), pp. 73–74). University of Nottingham.

[ece37133-bib-0054] Provan, J. , Soranzo, N. , Wilson, N. J. , Goldstein, D. B. , & Powell, W. (1999). A low mutation rate for chloroplast microsatellites. Genetics, 153(2), 943–947. 10511569 10.1093/genetics/153.2.943 PMC1460781

[ece37133-bib-0055] Purvis, A. , & Bromham, L. (1997). Estimating the transition/transversion ratio from independent pairwise comparisons with an assumed phylogeny. Journal of Molecular Evolution, 44(1), 112–119. 10.1007/PL00006117 9010143

[ece37133-bib-0056] Rambaut, A. (2012). FigTree v. 1.4.0. Institute of Evolutionary Biology, University of Edinburgh Retrieved from http://tree.bio.ed.ac.uk/software/figtree/

[ece37133-bib-0057] Randau, L. , Münch, R. , Hohn, M. J. , Jahn, D. , & Soll, D. (2005). Nanoarchaeum equitans creates functional tRNAs from separate genes for their 5'‐ and 3'‐halves. Nature, 433(7025), 537–541. 10.1038/nature03233 15690044

[ece37133-bib-0058] Ravi, V. , Khurana, J. P. , Tyagi, A. K. , & Khurana, P. (2008). An update on chloroplast genomes. Plant Systematics and Evolution, 271(1–2), 101–122. 10.1007/s00606-007-0608-0

[ece37133-bib-0059] Rice, D. W. , Alverson, A. J. , Richardson, A. O. , Young, G. J. , Sanchez‐Puerta, M. V. , Munzinger, J. , Barry, K. , Boore, J. L. , Zhang, Y. , dePamphilis, C. W. , Knox, E. B. , & Palmer, J. D. (2013). Horizontal transfer of entire genomes via mitochondrial fusion in the angiosperm *Amborella* . Science, 342(6165), 1468–1473. 10.1126/science.1246275 24357311

[ece37133-bib-0060] Rice, D. W. , & Palmer, J. D. (2006). An exceptional horizontal gene transfer in plastids: Gene replacement by a distant bacterial paralog and evidence that haptophyte and cryptophyte plastids are sisters. BMC Biology, 4, 31 10.1186/1741-7007-4-31 16956407 PMC1570145

[ece37133-bib-0061] Rieseberg, L. H. (1995). The role of hybridization in evolution: Old wine in new skins. American Journal of Botany, 82(7), 944–953. 10.1002/j.1537-2197.1995.tb15711.x

[ece37133-bib-0062] Salinas‐Giegé, T. , Giegé, R. , & Giegé, P. (2015). tRNA biology in mitochondria. International Journal of Molecular Sciences, 16(3), 4518–4559. 10.3390/ijms16034518 25734984 PMC4394434

[ece37133-bib-0063] Sharp, S. J. , Schaack, J. , Cooley, L. , Burke, D. , & Soll, D. (1985). Structure and transcription of eukaryotic tRNA genes. Critical Reviews in Biochemistry, 19(2), 107–144. 10.3109/10409238509082541 3905254

[ece37133-bib-0064] Spetea, C. , Hundal, T. , Lundin, B. , Heddad, M. , Adamska, I. , & Andersson, B. (2004). Multiple evidence for nucleotide metabolism in the chloroplast thylakoid lumen. Proceedings of the National Academy of Sciences of the United States of America, 101(5), 1409–1414. 10.1073/pnas.0308164100 14736920 PMC337066

[ece37133-bib-0065] Stamatakis, A. (2006). RAxML‐VI‐HPC: Maximum likelihood‐based phylogenetic analyses with thousands of taxa and mixed models. Bioinformatics, 22(21), 2688–2690. 10.1093/bioinformatics/btl446 16928733

[ece37133-bib-0066] Sugahara, J. , Fujishima, K. , Morita, K. , Tomita, M. , & Kanai, A. (2009). Disrupted tRNA gene diversity and possible evolutionary scenarios. Journal of Molecular Evolution, 69(5), 497–504. 10.1007/s00239-009-9294-6 19826747

[ece37133-bib-0067] Sun, F. J. , & Caetano‐Anollés, G. (2008). The origin and evolution of tRNA inferred from phylogenetic analysis of structure. Journal of Molecular Evolution, 66(1), 21–35. 10.1007/s00239-007-9050-8 18058157

[ece37133-bib-0068] Tamura, K. (1992). Estimation of the number of nucleotide substitutions when there are strong transition‐transversion and G+C‐content biases. Molecular Biology and Evolution, 9(4), 678–687. 1630306 10.1093/oxfordjournals.molbev.a040752

[ece37133-bib-0069] Varshney, U. , Lee, C. P. , Seong, B. L. , & RajBhandary, U. L. (1991). Mutants of initiator tRNA that function both as initiators and elongators. Journal of Biological Chemistry, 266(27), 18018–18024. 1917940

[ece37133-bib-0070] Wang, H. , Abassi, S. , & Ki, J. S. (2019). Origin and roles of a novel copper‐zinc superoxide dismutase (CuZnSOD) gene from the harmful dinoflagellate *Prorocentrum minimum* . Gene, 683, 113–122. 10.1016/j.gene.2018.10.013 30304703

[ece37133-bib-0071] Wang, W. , & Messing, J. (2011). High‐throughput sequencing of three Lemnoideae (duckweeds) chloroplast genomes from total DNA. PLoS One, 6(9), e24670 10.1371/journal.pone.0024670 21931804 PMC3170387

[ece37133-bib-0072] Wang, Y. L. , Guo, X. Y. , Hao, G. Q. , Wang, T. J. , & Wang, K. (2016). The complete chloroplast genome of *Sinadoxa corydalifolia* (Adoxaceae). Conservation Genetics Resources, 8(3), 303–305. 10.1007/s12686-016-0559-2

[ece37133-bib-0073] Wang, Y. , Zhu, F.‐C. , He, L.‐S. , & Danchin, A. (2018). Unique tRNA gene profile suggests paucity of nucleotide modifications in anticodons of a deep‐sea symbiotic Spiroplasma. Nucleic Acids Research, 46(5), 2197–2203. 10.1093/nar/gky045 29390076 PMC5861454

[ece37133-bib-0074] Weiner, A. M. , & Maizels, N. (1987). tRNA‐like structures tag the 3' ends of genomic RNA molecules for replication: Implications for the origin of protein synthesis. Proceedings of the National Academy of Sciences of the United States of America, 84(21), 7383–7387. 10.1073/pnas.84.21.7383 3478699 PMC299300

[ece37133-bib-0075] Weiner, A. M. , & Maizels, N. (1999). The genomic tag hypothesis: Modern viruses as molecular fossils of ancient strategies for genomic replication, and clues regarding the origin of protein synthesis. Biological Bulletin, 196(3), 327–330. 10.2307/1542962 10390830

[ece37133-bib-0076] Wicke, S. , Schneeweiss, G. M. , dePamphilis, C. W. , Muller, K. F. , & Quandt, D. (2011). The evolution of the plastid chromosome in land plants: Gene content, gene order, gene function. Plant Molecular Biology, 76(3–5), 273–297. 10.1007/s11103-011-9762-4 21424877 PMC3104136

[ece37133-bib-0077] Wilusz, J. E. (2015). Controlling translation via modulation of tRNA levels. Wiley Interdisciplinary Reviews: RNA, 6(4), 453–470. 10.1002/wrna.1287 25919480 PMC4478206

[ece37133-bib-0078] Winkworth, R. C. , Bell, C. D. , & Donoghue, M. J. (2008). Mitochondrial sequence data and Dipsacales phylogeny: Mixed models, partitioned Bayesian analyses, and model selection. Molecular Phylogenetics and Evolution, 46(3), 830–843. 10.1016/j.ympev.2007.11.021 18255318

[ece37133-bib-0079] Wise, R. , & Hoober, J. K. (2007). Advances in photosynthesis and respiration, Volume 23. The Structure and Function of Plastids (92(1), pp. 133–135). Photosynthesis Research.

[ece37133-bib-0080] Xu, Y. , Ma, P. , Shah, P. , Rokas, A. , Liu, Y. , & Johnson, C. H. (2013). Non‐optimal codon usage is a mechanism to achieve circadian clock conditionality. Nature, 495(7439), 116–120. 10.1038/nature11942 23417065 PMC3593822

[ece37133-bib-0081] Zhang, W. H. , Chen, Z. D. , Li, J. H. , Chen, H. B. , & Tang, Y. C. (2003). Phylogeny of the Dipsacales s.l. based on chloroplast *trnL*‐F and *ndhF* sequences. Molecular Phylogenetics and Evolution, 26(2), 176–189. 10.1016/S1055-7903(02)00303-2 12565029

[ece37133-bib-0082] Zhang, Y. , Li, L. , Yan, T. L. , & Liu, Q. (2014). Complete chloroplast genome sequences of *Praxelis* (*Eupatorium catarium* Veldkamp), an important invasive species. Gene, 549(1), 58–69. 10.1016/j.gene.2014.07.041 25042453

[ece37133-bib-0083] Zhang, Z. Y. , Zhou, Z. K. , & Gu, Z. J. (2002). Karyomorphology of *Heptacodium* (Caprifoliaceae s.str.) and its phylogenetic implications. Taxon, 51(3), 499–505.

[ece37133-bib-0084] Zhou, M. , Guo, J. H. , Cha, J. , Chae, M. , Chen, S. , Barral, J. M. , Sachs, M. S. , & Liu, Y. (2013). Non‐optimal codon usage affects expression, structure and function of clock protein FRQ. Nature, 495(7439), 111–115. 10.1038/nature11833 23417067 PMC3629845

[ece37133-bib-0085] Zuckerkandl, E. (1962). Molecular disease, evolution, and genic heterogeneity. Horizons in biochemistry (pp. 189–225). Academic.

