# Supplementary material for: Phylogeny and evolution of chloroplast tRNAs in Adoxaceae
Source: Ecol Evol. 2021 Jan 6;11(3):1294–309. doi: 10.1002/ece3.7133 (PMC7863635; doi:10.1002/ece3.7133)
Supplement: Supplementary file 1 — Fig S1 [file ECE3-11-1294-s001.pdf]

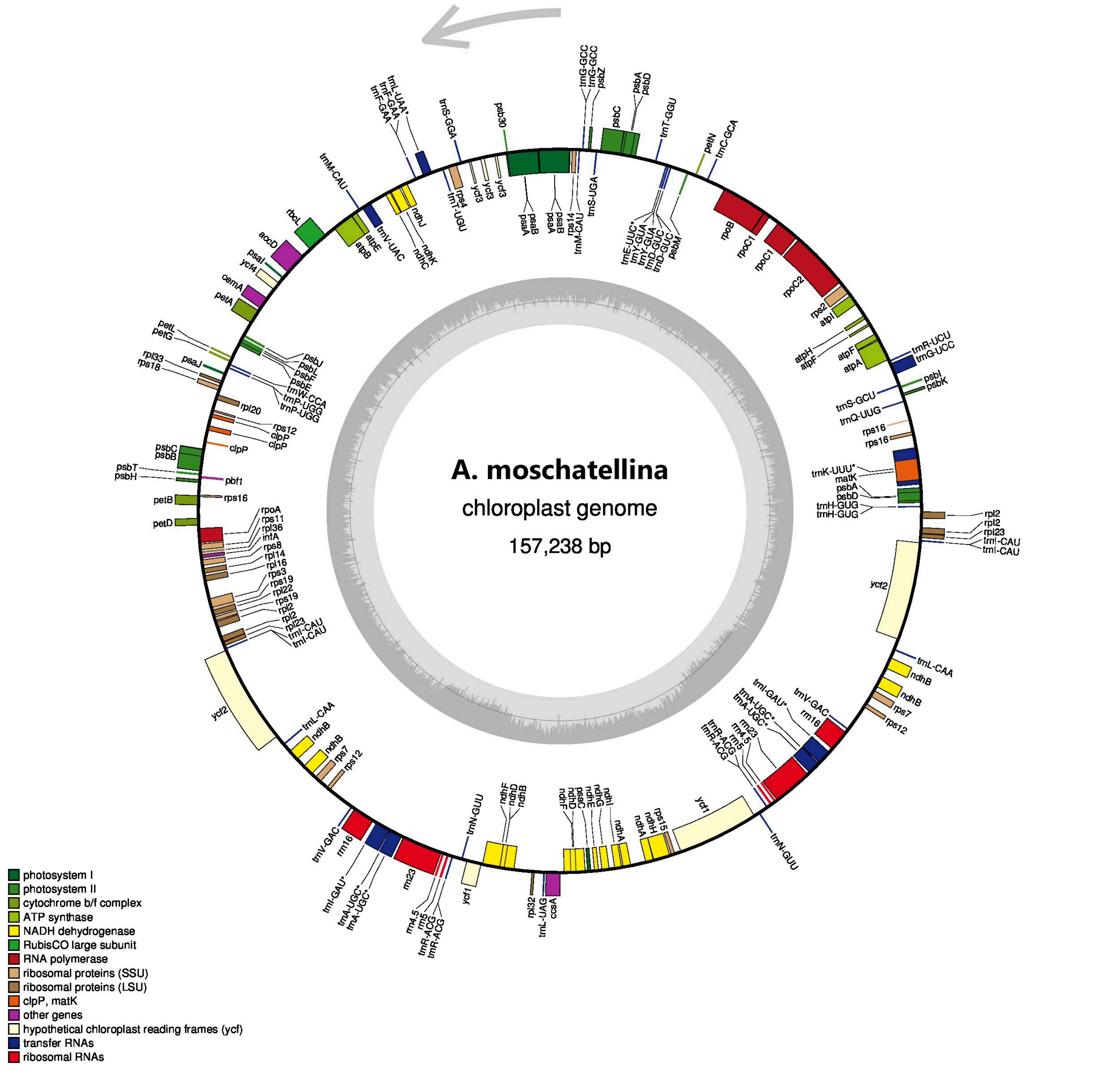
A. moschatellina
chloroplast genome
157,238 bp
photosystem I
photosystem II
cytochrome b/f complex
ATP synthase
NADH dehydrogenase
RubisCO large subunit
RNA polymerase
ribosomal proteins (SSU)
ribosomal proteins (LSU)
clpP, matK
other genes
hypothetical chloroplast reading frames (ycf)
transfer RNAs
ribosomal RNAs
trnL-UAA*
trnF-GAA
trnF-GAA
trnS-GGA
psb30
trnG-GCC
trnG-GCC
psbZ
psbC
psbA
psbD
trnT-GGU
petN
trnC-GCA
trnM-CAU
ndhJ
ndhK
ndhC
trnV-UAC
atpE
atpB
rbcL
accD
psaI
ycf4
cemA
petA
rps4
trnT-UGU
ycf3
psaB
psaA
rps14
trnS-UGA
trnE-UUC*
trnY-GUA
trnD-GUC
psbM
rpoB
rpoC1
rpoC2
rps2
atpI
atpH
atpF
atpA
trnR-UCU
trnG-UCC
psbI
psbK
trnS-GCU
trnQ-UUG
rps16
trnK-UUU*
matK
trnH-GUG
rpl2
rpl23
trnI-CAU
ycf2
trnL-CAA
ndhB
rps7
rps12
trnV-GAC
rrn16
trnI-GAU*
trnA-UGC*
rrn23
rrn4.5
rrn5
trnR-ACG
trnN-GUU
ycf1
rps15
ndhH
ndhA
ndhI
ndhG
ndhE
psaC
ndhD
ndhF
ccsA
trnL-UAG
rpl32
petL
petG
psbJ
psbL
psbF
psbE
trnW-CCA
trnP-UGG
rpl33
rps18
psaJ
rpl20
clpP
psbB
psbT
psbH
pbf1
petB
petD
rpoA
rps11
rpl36
infA
rps8
rpl14
rpl16
rps3
rps19
rpl22
