# Supplementary material for: Phylogeny and evolution of chloroplast tRNAs in Adoxaceae
Source: Ecol Evol. 2021 Jan 6;11(3):1294–309. doi: 10.1002/ece3.7133 (PMC7863635; doi:10.1002/ece3.7133)
Supplement: Supplementary file 2 — Table S1 [file ECE3-11-1294-s002.pdf]

Supplementary Table 1

Abrreviations: nucleotide composition of acceptor arm (AA), D-arm (DA), D-loop (DL), anti-codon arm (ACA), anti-codon loop (ACL), variable loop (VL), pseudouridine arm (ΨA) and pseudouridine loop (ΨL) of chloroplast tRNA.

Gene	Sequence(forward)	Begin	End	intron	Direction	Type	Anticodon	Length	AA	DA	DL	ACA	ACL	VL	ΨA	ΨL
S. corydalifolia _261	GCGGATGTAGCCAAGTGGATTAAGGCAGTGGATTGTGAATCCACCATTCGCGGGTTCAATTCCCGTCGTTCGCC	261	334	0	reverse	His	GUG	74	7	3	11	5	7	5	5	7
S. corydalifolia _1839	GGGTTGCTAACTCAACGGTAGAGTACTCGGCTTTTAACCGACTAGTTCCGGGTTCGAATCCCGGGCAACCCA	1,839	4,431	1	reverse	Lys	UUU	72	7	4	7	4	7	6	5	7
S. corydalifolia _7709	TGGGGCGTGGCCAAGTGGTAAGGCAACGGGTTTTGGTCCCGCTATTCGGAGGTTCGAATCCTTCCGTCCCAG	7,709	7,780	0	reverse	Gln	UUG	72	7	3	9	4	7	6	5	7
S. corydalifolia _8887	GGAGAGATGGCTGAGTGGACTAAAGCGGCGGATTGCTAATCCGTTGTACGAGTTATTCGTACCGAGGGTTCGAATCCCTCTCTTTCCG	8,887	8,974	0	reverse	Ser	GCU	88	7	3	11	5	7	19	5	7
S. corydalifolia _9728	GCGGGTATAGTTTAGTGGTAAAACCCTAGCCTTCCAAGCTAACGATGCGGGTTCGATTCCCGCTACCCGC	9,728	10,489	1	forward	Gly	UCC	70	7	4	7	4	7	5	5	7
S. corydalifolia _10668	GCGTCCATTGTCTAATGGATAGGACAGAGGTCTTCTAAACCTTTGGTATAGGTTCAAATCCTATTGGACGCA	10,668	10,739	0	forward	Arg	UCU	72	7	4	8	5	7	4	5	7
S. corydalifolia _28834	GGCGGCATGGCCGAGTGGTAAGGCGGGGGACTGCAAATCCTTTTTCCCCAGTTCAAATCCGGGTGTCGCCT	28,834	28,904	0	forward	Cys	GCA	71	7	3	9	5	7	4	5	7
S. corydalifolia _31544	GGGATTGTAGTTCAATTGGTCAGAGCACCGCCCTGTCAAGGCGGAAGCTGCGGGTTCGAGCCCCGTCAGTCCCG	31,544	31,617	0	reverse	Asp	GUC	74	7	4	9	5	7	5	5	7
S. corydalifolia _31723	GGGTCGATGCCCGAGCGGTTAATGGGGACGGACTGTAAATTCGTTGGCAATATGTCTACGCTGGTTCAAATCCAGCTCGGCCCA	31,723	31,806	0	reverse	Tyr	GUA	84	7	4	9	5	7	4	5	7
S. corydalifolia _31871	GCCCCCATCGTCTAGTGGTTCAGGACATCTCTCTTTCAAGGAGGCGGCGGGGATTCGACTTCCCCTGGGGGTA	31,871	31,943	0	reverse	Glu	UUC	73	7	4	9	5	7	4	5	7
S. corydalifolia _32668	GCCCCTTTAACTCAGCGGTAGAGTAACGCCATGGTAAGGCGTAAGTCATCGGTTCAAATCCGATAAGGGGCT	32,668	32,739	0	forward	Thr	GGU	72	7	4	7	5	7	5	5	7
S. corydalifolia _36817	GGAGAGATGGCTGAGTGGTTGATAGCTCCGGTCTTGAAAACCGGTATAGTTTTGAACAAAGAACTATCGAGGGTTCGAATCCCTCTCTCTCCT	36,817	36,909	0	reverse	Ser	UGA	93	7	4	9	5	7	24	5	7
S. corydalifolia _37776	GCGGATATGGTCGAATGGTAAAATTTCTCTTTGCCAAGGAGAAGACGCGGGTTCGATTCCCGCTATCCGCC	37,776	37,846	0	forward	Gly	GCC	71	7	2	11	5	7	4	5	7
S. corydalifolia _38018	CGCGGGGTAGAGCAGTTTGGTAGCTCGCAAGGCTCATAACCTTGAGGTCACGGGTTCAAATCCTGTCTCCGCAA	38,018	38,091	0	reverse	fMet	CAU	74	6	4	9	5	7	5	5	7
S. corydalifolia _46275	GGAGAGATGGCCGAGTGGTTGAAGGCGTAGCATTGGAACTGCTATGTAGGCTTTTGTTTACCGAGGGTTCGAATCCCTCTCTTTCCG	46,275	46,361	0	forward	Ser	GGA	87	7	3	11	5	7	18	5	7
S. corydalifolia _47633	GCCTGCTTAGCTCAGAGGTTAGAGCATCGCATTTGTAATGCGATGGTCATCGGTTCGATTCCGATAGCCGGCT	47,633	47,705	0	reverse	Thr	UGU	73	7	4	8	5	7	5	5	7
S. corydalifolia _48473	GGGGATATGGCGAAATTGGTAGACGCTACGGACTTAAAAAATCCGTCGACTTTATAAATCGTGAGGGTTCAAGTCCCTCTATCCCCA	48,473	49,062	1	forward	Leu	UAA	87	7	3	11	5	9	16	5	7
S. corydalifolia _49559	GTCGGGATAGCTCAGCTGGTAGAGCAGAGGACTGAAAATCCTCGTGTCACCAGTTCAAATCTGGTTCCTGGCA	49,559	49,631	0	forward	Phe	GAA	73	7	4	8	5	7	5	5	7
S. corydalifolia _53253	AGGGCTATAGCTCAGTTGGTAGAGCACCTCGTTTACACACCGAGAATGTCTACGGTTCGAGTCCGTATAGCCCTA	53,253	53,902	1	reverse	Val	UAC	75	7	4	8	4	9	6	5	7
S. corydalifolia _54095	CCTACTTAACTCAGTGGTTAGAGTATTGCTTTCATACGGCGGGAGTCATTGGTTCAAATCCAATAGTAGGTA	54,095	54,166	0	forward	Met	CAU	72	6	4	8	5	7	5	5	7
S. corydalifolia _68258	GCGCTCTTAGTTCAGTTCGGTAGAACGTGGGTCTCCAAAACCCGATGTCGTAGGTTCAAATCCTACAGAGCGTG	68,258	68,331	0	reverse	Trp	CCA	74	7	4	9	5	7	5	5	7
S. corydalifolia _68493	AGGGATGTGGCGCAGCTTGGTAGCGCGTTTGTTTTGGGTACAAAATGTCACAGGTTCAAATCCTGTCATCCCTA	68,493	68,566	0	reverse	Pro	UGG	74	7	4	9	5	7	5	5	7
S. corydalifolia _88447	GCATCCATGGCTGAATGGTTAAAGCGCCCAACTCATAATTGGCGAATTCGTAGGTTCAATTCCTACTGGATGCA	88,447	88,520	0	reverse	Ile2	CAU	74	7	3	10	5	7	5	5	7
S. corydalifolia _96228	GCCTTGGTGGTGAAACGGTAGACACGCGAGACTCAAAATCTCGTGCTCAAGAGCGTGGAGGTTCGAGTCCTCTTCAAGGCA	96,228	96,308	0	reverse	Leu	CAA	81	7	3	10	5	7	13	5	7
S. corydalifolia _102483	AGGGATATAACTCAGCGGTAGAGTGTCACCTTGACGTGGTGGAAGTCATCAGTTCGAGCCTGATTATCCCTA	102,483	102,554	0	forward	Val	GAC	72	7	4	7	5	7	5	5	7
S. corydalifolia _104558	GGGCTATTAGCTCAGTGGTAGAGCGCGCCCCTGATAATTGCGGGGCGAGGTCTCTGGTTCAAGTCCAGGATGGCCCA	104,558	105,574	1	forward	Ile	GAU	77	7	4	7	5	12	5	5	7
S. corydalifolia _105633	GGGGATATAGCTCAGTTGGTAGAGCTCCGCTCTTGCAAGGCGGATGTCAGCGGTTCGAGTCCGCTTATCTCCA	105,633	106,520	1	forward	Ala	UGC	73	7	4	8	5	7	5	5	7
S. corydalifolia _110289	GGGCCTGTAGCTCAGAGGATTAGAGCACGTGGCTACGAACCACGGTGTCGGGGGTTCGAATCCCTCCTCGCCCA	110,289	110,362	0	forward	Arg	ACG	74	7	4	9	5	7	5	5	7
S. corydalifolia _110967	TCCTCAGTAGCTCAGTGGTAGAGCGGTCGGCTGTTAACCGATTGGTCGTAGGTTCGAATCCTACTTGGGGAG	110,967	111,038	0	reverse	Asn	GUU	72	7	4	7	5	7	5	5	7
S. corydalifolia _116738	GCCGCTATGGTGAAATCGGTAGACACGCTGCTCTTAGGAAGCAGTGCTAGAGCATCTCGGTTCGAGTCCGAGTGGCGGCA	116,738	116,817	0	forward	Leu	UAG	80	7	3	11	5	7	11	5	7
S. corydalifolia _132467	TCCTCAGTAGCTCAGTGGTAGAGCGGTCGGCTGTTAACCGATTGGTCGTAGGTTCGAATCCTACTTGGGGAG	132,467	132,538	0	forward	Asn	GUU	72	7	4	7	5	7	5	5	7
S. corydalifolia _133143	GGGCCTGTAGCTCAGAGGATTAGAGCACGTGGCTACGAACCACGGTGTCGGGGGTTCGAATCCCTCCTCGCCCA	133,143	133,216	0	reverse	Arg	ACG	74	7	4	9	5	7	5	5	7
S. corydalifolia _136985	GGGGATATAGCTCAGTTGGTAGAGCTCCGCTCTTGCAAGGCGGATGTCAGCGGTTCGAGTCCGCTTATCTCCA	136,985	137,872	1	reverse	Ala	UGC	73	7	4	8	5	7	5	5	7
S. corydalifolia _137931	GGGCTATTAGCTCAGTGGTAGAGCGCGCCCCTGATAATTGCGGGGCGAGGTCTCTGGTTCAAGTCCAGGATGGCCCA	137,931	138,947	1	reverse	Ile	GAU	77	7	4	7	5	12	5	5	7
S. corydalifolia _140951	AGGGATATAACTCAGCGGTAGAGTGTCACCTTGACGTGGTGGAAGTCATCAGTTCGAGCCTGATTATCCCTA	140,951	141,022	0	reverse	Val	GAC	72	7	4	7	5	7	5	5	7
S. corydalifolia _147197	GCCTTGGTGGTGAAACGGTAGACACGCGAGACTCAAAATCTCGTGCTCAAGAGCGTGGAGGTTCGAGTCCTCTTCAAGGCA	147,197	147,277	0	forward	Leu	CAA	81	7	3	10	5	7	13	5	7
S. corydalifolia _154985	GCATCCATGGCTGAATGGTTAAAGCGCCCAACTCATAATTGGCGAATTCGTAGGTTCAATTCCTACTGGATGCA	154,985	155,058	0	forward	Ile2	CAU	74	7	3	10	5	7	5	5	7
A. moschatellina _262	GCGGATGTAGCCAAGTGGATTAAGGCAGTGGATTGTGAATCCACCATTCGCGGGTTCAATTCCCGTCGTTCGCC	262	335	0	reverse	His	GUG	74	7	3	11	5	7	5	5	7
A. moschatellina _1841	GGGTTGCTAACTCAACGGTAGAGTACTCGGCTTTTAACCGACTAGTTCCGGGTTCGAATCCCGGGCAACCCA	1,841	4,433	1	reverse	Lys	UUU	72	7	4	7	4	7	6	5	7
A. moschatellina _7675	TGGGGCGTGGCCAAGTGGTAAGGCAACGGGTTTTGATCCCGCTATTCGGAGGTTCGAATCCTTCCGTCCCAG	7,675	7,746	0	reverse	Gln	UUG	72	7	3	9	4	7	6	5	7
A. moschatellina _8841	GGAGAGATGGCTGAGTGGACTAAAGCGGCGGATTGCTAATCCGTTGTACGAGTTATTCGTACCGAGGGTTCGAATCCCTCTCTTTCCG	8,841	8,928	0	reverse	Ser	GCU	88	7	3	11	5	7	18	5	7
A. moschatellina _9688	GCGGGTATAGTTTAGTGGTAAAACCCTAGCCTTCCAAGCTAACGATGCGGGTTCGATTCCCGCTACCCGC	9,688	10,461	1	forward	Gly	UCC	70	7	4	7	4	7	5	5	7
A. moschatellina _10639	GCGTCCATTGTCTAATGGATAGGACAGAGGTCTTCTAAACCTTTGGTATAGGTTCAAATCCTATTGGACGCA	10,639	10,710	0	forward	Arg	UCU	72	7	4	8	5	7	4	5	7
A. moschatellina _28782	GGCGGCATGGCCGAGTGGTAAGGCGGGGGACTGCAAATCCTTTTTCCCCAGTTCAAATCCGGGTGTCGCCT	28,782	28,852	0	forward	Cys	GCA	71	7	3	9	5	7	4	5	7
A. moschatellina _31464	GGGATTGTAGTTCAATTGGTCAGAGCACCGCCCTGTCAAGGCGGAAGCTGCGGGTTCGAGCCCCGTCAGTCCCG	31,464	31,537	0	reverse	Asp	GUC	74	7	4	9	5	7	5	5	7
A. moschatellina _31642	GGGTCGATGCCCGAGCGGTTAATGGGGACGGACTGTAAATTCGTTGGCAATATGTCTACGCTGGTTCAAATCCAGCTCGGCCCA	31,642	31,725	0	reverse	Tyr	GUA	84	7	4	9	5	7	4	5	7
A. moschatellina _31790	GCCCCCATCGTCTAGTGGTTCAGGACATCTCTCTTTCAAGGAGGCGGCGGGGATTCGACTTCCCCTGGGGGTA	31,790	31,862	0	reverse	Glu	UUC	73	7	4	9	5	7	4	5	7
A. moschatellina _32589	GCCCCTTTAACTCAGCGGTAGAGTAACGCCATGGTAAGGCGTAAGTCATCGGTTCAAATCCGATAAGGGGCT	32,589	32,660	0	forward	Thr	GGU	72	7	4	7	5	7	5	5	7
A. moschatellina _36732	GGAGAGATGGCTGAGTGGTTGATAGCTCCGGTCTTGAAAACCGGTATAGTTTTGAACAAAGAACTATCGAGGGTTCGAATCCCTCTCTCTCCT	36,732	36,824	0	reverse	Ser	UGA	93	7	4	9	5	7	24	5	7
A. moschatellina _37691	GCGGATATGGTCGAATGGTAAAATTTCTCTTTGCCAAGGAGAAGACGCGGGTTCGATTCCCGCTATCCGCC	37,691	37,761	0	forward	Gly	GCC	71	7	2	11	5	7	4	5	7
A. moschatellina _37933	CGCGGGGTAGAGCAGTTTGGTAGCTCGCAAGGCTCATAACCTTGAGGTCACGGGTTCAAATCCTGTCTCCGCAA	37,933	38,006	0	reverse	fMet	CAU	74	6	4	9	5	7	5	5	7
A. moschatellina _46198	GGAGAGATGGCCGAGTGGTTGAAGGCGTAGCATTGGAACTGCTATGTAGGCTTTTGTTTACCGAGGGTTCGAATCCCTCTCTTTCCG	46,198	46,284	0	forward	Ser	GGA	87	7	3	11	5	7	18	5	7
A. moschatellina _47541	GCCTGCTTAGCTCAGAGGTTAGAGCATCGCATTTGTAATGCGATGGTCATCGGTTCGATTCCGATAGCCGGCT	47,541	47,613	0	reverse	Thr	UGU	73	7	4	8	5	7	5	5	7
A. moschatellina _48385	GGGGATATGGCGAAATTGGTAGACGCTACGGACTTAAAAAATCCGTCGACTTTATAAATCGTGAGGGTTCAAGTCCCTCTATCCCCA	48,385	48,980	1	forward	Leu	UAA	87	7	3	11	5	9	16	5	7
A. moschatellina _49526	GTCGGGATAGCTCAGCTGGTAGAGCAGAGGACTGAAAATCCTCGTGTCACCAGTTCAAATCTGGTTCCTGGCA	49,526	49,598	0	forward	Phe	GAA	73	7	4	8	5	7	5	5	7
A. moschatellina _53131	AGGGCTATAGCTCAGTTGGTAGAGCACCTCGTTTACACACCGAGAATGTCTACGGTTCGAGTCCGTATAGCCCTA	53,131	53,781	1	reverse	Val	UAC	75	7	4	8	4	9	6	5	7
A. moschatellina _53974	CCTACTTAACTCAGTGGTTAGAGTATTGCTTTCATACGGCGGGAGTCATTGGTTCAAATCCAATAGTAGGTA	53,974	54,045	0	forward	Met	CAU	72	6	4	8	5	7	5	5	7
A. moschatellina _68143	GCGCTCTTAGTTCAGTTCGGTAGAACGTGGGTCTCCAAAACCCGATGTCGTAGGTTCAAATCCTACAGAGCGTG	68,143	68,216	0	reverse	Trp	CCA	74	7	4	9	5	7	5	5	7
A. moschatellina _68378	AGGGATGTGGCGCAGCTTGGTAGCGCGTTTGTTTTGGGTACAAAATGTCACAGGTTCAAATCCTGTCATCCCTA	68,378	68,451	0	reverse	Pro	UGG	74	7	4	9	5	7	5	5	7
A. moschatellina _88365	GCATCCATGGCTGAATGGTTAAAGCGCCCAACTCATAATTGGCGAATTCGTAGGTTCAATTCCTACTGGATGCA	88,365	88,438	0	reverse	Ile2	CAU	74	7	3	10	5	7	5	5	7
A. moschatellina _96140	GCCTTGGTGGTGAAACGGTAGACACGCGAGACTCAAAATCTCGTGCTCAAGAGCGTGGAGGTTCGAGTCCTCTTCAAGGCA	96,140	96,220	0	reverse	Leu	CAA	81	7	3	10	5	7	13	5	7
A. moschatellina _102545	AGGGATATAACTCAGCGGTAGAGTGTCACCTTGACGTGGTGGAAGTCATCAGTTCGAGCCTGATTATCCCTA	102,545	102,616	0	forward	Val	GAC	72	7	4	7	5	7	5	5	7
A. moschatellina _104620	GGGCTATTAGCTCAGTGGTAGAGCGCGCCCCTGATAATTGCGGGGCGAGGTCTCTGGTTCAAGTCCAGGATGGCCCA	104,620	105,637	1	forward	Ile	GAU	77	7	4	7	5	12	5	5	7
A. moschatellina _105701	GGGGATATAGCTCAGTTGGTAGAGCTCCGCTCTTGCAAGGCGGATGTCAGCGGTTCGAGTCCGCTTATCTCCA	105,701	106,588	1	forward	Ala	UGC	73	7	4	8	5	7	5	5	7
A. moschatellina _110341	GGGCCTGTAGCTCAGAGGATTAGAGCACGTGGCTACGAACCACGGTGTCGGGGGTTCGAATCCCTCCTCGCCCA	110,341	110,414	0	forward	Arg	ACG	74	7	4	9	5	7	5	5	7
A. moschatellina _111019	TCCTCAGTAGCTCAGTGGTAGAGCGGTCGGCTGTTAACCGATTGGTCGTAGGTTCGAATCCTACTTGGGGAG	111,019	111,090	0	reverse	Asn	GUU	72	7	4	7	5	7	5	5	7
A. moschatellina _116790	GCCGCTATGGTGAAATCGGTAGACACGCTGCTCTTAGGAAGCAGTGCTAGAGCATCTCGGTTCGAGTCCGAGTGGCGGCA	116,790	116,869	0	forward	Leu	UAG	80	7	3	11	5	7	11	5	7
A. moschatellina _132489	TCCTCAGTAGCTCAGTGGTAGAGCGGTCGGCTGTTAACCGATTGGTCGTAGGTTCGAATCCTACTTGGGGAG	132,489	132,560	0	forward	Asn	GUU	72	7	4	7	5	7	5	5	7
A. moschatellina _133165	GGGCCTGTAGCTCAGAGGATTAGAGCACGTGGCTACGAACCACGGTGTCGGGGGTTCGAATCCCTCCTCGCCCA	133,165	133,238	0	reverse	Arg	ACG	74	7	4	9	5	7	5	5	7
A. moschatellina _136991	GGGGATATAGCTCAGTTGGTAGAGCTCCGCTCTTGCAAGGCGGATGTCAGCGGTTCGAGTCCGCTTATCTCCA	136,991	137,878	1	reverse	Ala	UGC	73	7	4	8	5	7	5	5	7
A. moschatellina _137942	GGGCTATTAGCTCAGTGGTAGAGCGCGCCCCTGATAATTGCGGGGCGAGGTCTCTGGTTCAAGTCCAGGATGGCCCA	137,942	138,959	1	reverse	Ile	GAU	77	7	4	7	5	12	5	5	7
A. moschatellina _140963	AGGGATATAACTCAGCGGTAGAGTGTCACCTTGACGTGGTGGAAGTCATCAGTTCGAGCCTGATTATCCCTA	140,963	141,034	0	reverse	Val	GAC	72	7	4	7	5	7	5	5	7
A. moschatellina _147359	GCCTTGGTGGTGAAACGGTAGACACGCGAGACTCAAAATCTCGTGCTCAAGAGCGTGGAGGTTCGAGTCCTCTTCAAGGCA	147,359	147,439	0	forward	Leu	CAA	81	7	3	10	5	7	13	5	7
A. moschatellina _155141	GCATCCATGGCTGAATGGTTAAAGCGCCCAACTCATAATTGGCGAATTCGTAGGTTCAATTCCTACTGGATGCA	155,141	155,214	0	forward	Ile2	CAU	74	7	3	10	5	7	5	5	7
T. omeiensis _331	GCGGATGTAGCCAAGTGGATTAAGGCAGTGGATTGTGAATCCACCATTCGCGGGTTCAATTCCCGTCGTTCGCC	331	404	0	reverse	His	GUG	74	7	3	11	5	7	5	5	7
T. omeiensis _1911	GGGTTGCTAACTCAACGGTAGAGTACTCGGCTTTTAACCGACTAGTTCCGGGTTCGAATCCCGGGCAACCCA	1,911	4,503	1	reverse	Lys	UUU	72	7	4	7	4	7	6	5	7
T. omeiensis _7756	TGGGGCGTGGCCAAGTGGTAAGGCAACGGGTTTTGGTCCCGCTATTCGGAGGTTCGAATCCTTCCGTCCCAG	7,756	7,827	0	reverse	Gln	UUG	72	7	3	9	4	7	6	5	7
T. omeiensis _8919	GGAGAGATGGCTGAGTGGACTAAAGCGGCGGATTGCTAATCCGTTGTACGAGTTATTCGTACCGAGGGTTCGAATCCCTCTCTTTCCG	8,919	9,006	0	reverse	Ser	GCU	88	7	3	11	5	7	19	5	7
T. omeiensis _9740	GCGGGTATAGTTTAGTGGTAAAACCCTAGCCTTCCAAGCTAACGATGCGGGTTCGATTCCCGCTACCCGC	9,740	10,513	1	forward	Gly	UCC	70	7	4	7	4	7	5	5	7
T. omeiensis _10700	GCGTCCATTGTCTAATGGATAGGACAGAGGTCTTCTAAACCTTTGGTATAGGTTCAAATCCTATTGGACGCA	10,700	10,771	0	forward	Arg	UCU	72	7	4	8	5	7	4	5	7
T. omeiensis _28829	GGCGGCATGGCCGAGTGGTAAGGCGGGGGACTGCAAATCCTTTTTCCCCAGTTCAAATCCGGGTGTCGCCT	28,829	28,899	0	forward	Cys	GCA	71	7	3	9	5	7	4	5	7
T. omeiensis _31515	GGGATTGTAGTTCAATTGGTCAGAGCACCGCCCTGTCAAGGCGGAAGCTGCGGGTTCGAGCCCCGTCAGTCCCG	31,515	31,588	0	reverse	Asp	GUC	74	7	4	9	5	7	5	5	7
T. omeiensis _31693	GGGTCGATGCCCGAGCGGTTAATGGGGACGGACTGTAAATTCGTTGGCAATATGTCTACGCTGGTTCAAATCCAGCTCGGCCCA	31,693	31,776	0	reverse	Tyr	GUA	84	7	4	9	5	7	4	5	7
T. omeiensis _31841	GCCCCCATCGTCTAGTGGTTCAGGACATCTCTCTTTCAAGGAGGCGGCGGGGATTCGACTTCCCCTGGGGGTA	31,841	31,913	0	reverse	Glu	UUC	73	7	4	9	5	7	4	5	7
T. omeiensis _32640	CCCTTTAACTCAGCGGTAGAGTAACGCCATGGTAAGGCGTAAGTCATCGGTTCAAATCCGATAAGGGGCT	32,640	32,709	0	forward	Thr	GGU	70	5	4	7	5	7	5	5	7
T. omeiensis _36777	GGAGAGATGGCTGAGTGGTTGATAGCTCCGGTCTTGAAAACCGGTATAGTTTTGAACAAAGAACTATCGAGGGTTCGAATCCCTCTCTCTCCT	36,777	36,869	0	reverse	Ser	UGA	93	7	4	9	5	7	24	5	7
T. omeiensis _37737	GCGGATATGGTCGAATGGTAAAATTTCTCTTTGCCAAGGAGAAGACGCGGGTTCGATTCCCGCTATCCGCC	37,737	37,807	0	forward	Gly	GCC	71	7	2	11	5	7	4	5	7
T. omeiensis _37979	CGCGGGGTAGAGCAGTTTGGTAGCTCGCAAGGCTCATAACCTTGAGGTCACGGGTTCAAATCCTGTCTCCGCAA	37,979	38,052	0	reverse	fMet	CAU	74	6	4	9	5	7	5	5	7
T. omeiensis _46240	GGAGAGATGGCCGAGTGGTTGAAGGCGTAGCATTGGAACTGCTATGTAGGCTTTTGTTTACCGAGGGTTCGAATCCCTCTCTTTCCG	46,240	46,326	0	forward	Ser	GGA	87	7	3	11	5	7	18	5	7
T. omeiensis _47591	GCCTGCTTAGCTCAGAGGTTAGAGCATCGCATTTGTAATGCGATGGTCATCGGTTCGATTCCGATAGCCGGCT	47,591	47,663	0	reverse	Thr	UGU	73	7	4	8	5	7	5	5	7
T. omeiensis _48411	GGGGATATGGCGAAATTGGTAGACGCTACGGACTTAAAAAATCCGTCGACTTTATAAATCGTGAGGGTTCAAGTCCCTCTATCCCCA	48,411	49,006	1	forward	Leu	UAA	87	7	3	11	5	9	16	5	7
T. omeiensis _49590	GTCGGGATAGCTCAGCTGGTAGAGCAGAGGACTGAAAATCCTCGTGTCACCAGTTCAAATCTGGTTCCTGGCA	49,590	49,662	0	forward	Phe	GAA	73	7	4	8	5	7	5	5	7
T. omeiensis _53193	AGGGCTATAGCTCAGTTGGTAGAGCACCTCGTTTACACACCGAGAATGTCTACGGTTCGAGTCCGTATAGCCCTA	53,193	53,839	1	reverse	Val	UAC	75	7	4	8	4	9	6	5	7
T. omeiensis _54032	CCTACTTAACTCAGTGGTTAGAGTATTGCTTTCATACGGCGGGAGTCATTGGTTCAAATCCAATAGTAGGTA	54,032	54,103	0	forward	Met	CAU	72	6	4	8	5	7	5	5	7
T. omeiensis _68433	GCGCTCTTAGTTCAGTTCGGTAGAACGTGGGTCTCCAAAACCCGATGTCGTAGGTTCAAATCCTACAGAGCGTG	68,433	68,506	0	reverse	Trp	CCA	74	7	4	9	5	7	5	5	7
T. omeiensis _68668	AGGGATGTGGCGCAGCTTGGTAGCGCGTTTGTTTTGGGTACAAAATGTCACAGGTTCAAATCCTGTCATCCCTA	68,668	68,741	0	reverse	Pro	UGG	74	7	4	9	5	7	5	5	7
T. omeiensis _88597	GCATCCATGGCTGAATGGTTAAAGCGCCCAACTCATAATTGGCGAATTCGTAGGTTCAATTCCTACTGGATGCA	88,597	88,670	0	reverse	Ile2	CAU	74	7	3	10	5	7	5	5	7
T. omeiensis _96354	GCCTTGGTGGTGAAACGGTAGACACGCGAGACTCAAAATCTCGTGCTCAAGAGCGTGGAGGTTCGAGTCCTCTTCAAGGCA	96,354	96,434	0	reverse	Leu	CAA	81	7	3	10	5	7	13	5	7
T. omeiensis _102753	AGGGATATAACTCAGCGGTAGAGTGTCACCTTGACGTGGTGGAAGTCATCAGTTCGAGCCTGATTATCCCTA	102,753	102,824	0	forward	Val	GAC	72	7	4	7	5	7	5	5	7
T. omeiensis _104828	GGGCTATTAGCTCAGTGGTAGAGCGCGCCCCTGATAATTGCGGGGCGAGGTCTCTGGTTCAAGTCCAGGATGGCCCA	104,828	105,845	1	forward	Ile	GAU	77	7	4	7	5	12	5	5	7
T. omeiensis _105909	GGGGATATAGCTCAGTTGGTAGAGCTCCGCTCTTGCAAGGCGGATGTCAGCGGTTCGAGTCCGCTTATCTCCA	105,909	106,796	1	forward	Ala	UGC	73	7	4	8	5	7	5	5	7
T. omeiensis _110558	GGGCCTGTAGCTCAGAGGATTAGAGCACGTGGCTACGAACCACGGTGTCGGGGGTTCGAATCCCTCCTCGCCCA	110,558	110,631	0	forward	Arg	ACG	74	7	4	9	5	7	5	5	7
T. omeiensis _111236	TCCTCAGTAGCTCAGTGGTAGAGCGGTCGGCTGTTAACCGATTGGTCGTAGGTTCGAATCCTACTTGGGGAG	111,236	111,307	0	reverse	Asn	GUU	72	7	4	7	5	7	5	5	7
T. omeiensis _116957	GCCGCTATGGTGAAATCGGTAGACACGCTGCTCTTAGGAAGCAGTGCTAGAGCATCTCGGTTCGAGTCCGAGTGGCGGCA	116,957	117,036	0	forward	Leu	UAG	80	7	3	11	5	7	11	5	7
T. omeiensis _132722	TCCTCAGTAGCTCAGTGGTAGAGCGGTCGGCTGTTAACCGATTGGTCGTAGGTTCGAATCCTACTTGGGGAG	132,722	132,793	0	forward	Asn	GUU	72	7	4	7	5	7	5	5	7
T. omeiensis _133398	GGGCCTGTAGCTCAGAGGATTAGAGCACGTGGCTACGAACCACGGTGTCGGGGGTTCGAATCCCTCCTCGCCCA	133,398	133,471	0	reverse	Arg	ACG	74	7	4	9	5	7	5	5	7
T. omeiensis _137233	GGGGATATAGCTCAGTTGGTAGAGCTCCGCTCTTGCAAGGCGGATGTCAGCGGTTCGAGTCCGCTTATCTCCA	137,233	138,120	1	reverse	Ala	UGC	73	7	4	8	5	7	5	5	7
T. omeiensis _138184	GGGCTATTAGCTCAGTGGTAGAGCGCGCCCCTGATAATTGCGGGGCGAGGTCTCTGGTTCAAGTCCAGGATGGCCCA	138,184	139,201	1	reverse	Ile	GAU	77	7	4	7	5	12	5	5	7
T. omeiensis _141205	AGGGATATAACTCAGCGGTAGAGTGTCACCTTGACGTGGTGGAAGTCATCAGTTCGAGCCTGATTATCCCTA	141,205	141,276	0	reverse	Val	GAC	72	7	4	7	5	7	5	5	7
T. omeiensis _147595	GCCTTGGTGGTGAAACGGTAGACACGCGAGACTCAAAATCTCGTGCTCAAGAGCGTGGAGGTTCGAGTCCTCTTCAAGGCA	147,595	147,675	0	forward	Leu	CAA	81	7	3	10	5	7	13	5	7
T. omeiensis _155359	GCATCCATGGCTGAATGGTTAAAGCGCCCAACTCATAATTGGCGAATTCGTAGGTTCAATTCCTACTGGATGCA	155,359	155,432	0	forward	Ile2	CAU	74	7	3	10	5	7	5	5	7
S. williamsii _5	GCGGATGTAGCCAAGTGGATTAAGGCAGTGGATTGTGAATCCACTATTCGCGGGTTCAATTCCCGTCGTTCGCC	5	78	0	reverse	His	GUG	74	7	3	11	5	7	5	5	7
S. williamsii _1819	GGGTTGCTAACTCAACGGTAGAGTACTCGGCTTTTAACCGACTAGTTCCGGGTTCGAATCCCGGGCAACCCA	1,819	4,412	1	reverse	Lys	UUU	72	7	4	7	4	7	6	5	7
S. williamsii _7905	TGGGGCGTGGCCAAGTGGTAAGGCAACGGGTTTTGGTCCCGCTATTCGGAGGTTCGAATCCTTCCGTCCCAG	7,905	7,976	0	reverse	Gln	UUG	72	7	3	9	4	7	6	5	7
S. williamsii _9088	GGAGAGATGGCTGAGTGGACTAAAGCGGCGGATTGCTAATCCGTTGTACGAGTTATTCGTACCGAGGGTTCGAATCCCTCTCTTTCCG	9,088	9,175	0	reverse	Ser	GCU	88	7	3	11	5	7	19	5	7
S. williamsii _9921	GCGGGTATAGTTTAGTGGTAAAACCCTAGCCTTCCAAGCTAACGATGCGGGTTCGATTCCCGCTACCCGC	9,921	10,695	1	forward	Gly	UCC	70	7	4	7	4	7	5	5	7
S. williamsii _10898	GCGTCCATTGTCTAATGGATAGGACAGAGGTCTTCTAAACCTTTGGTATAGGTTCAAATCCTATTGGACGCA	10,898	10,969	0	forward	Arg	UCU	72	7	4	8	5	7	4	5	7
S. williamsii _29055	GGCGGCATGGCCGAGTGGTAAGGCGGGGGACTGCAAATCCTTTTTCCCCAGTTCAAATCCGGGTGTCGCCT	29,055	29,125	0	forward	Cys	GCA	71	7	3	9	5	7	4	5	7
S. williamsii _31763	GGGATTGTAGTTCAATTGGTCAGAGCACCGCCCTGTCAAGGCGGAAGCTGCGGGTTCGAGCCCCGTCAGTCCCG	31,763	31,836	0	reverse	Asp	GUC	74	7	4	9	5	7	5	5	7
S. williamsii _31941	GGGTCGATGCCCGAGCGGTTAATGGGGACGGACTGTAAATTCGTTGGCAATATGTCTACGCTGGTTCAAATCCAGCTCGGCCCA	31,941	32,024	0	reverse	Tyr	GUA	84	7	4	9	5	7	4	5	7
S. williamsii _32089	GCCCCCATCGTCTAGTGGTTCAGGACATCTCTCTTTCAAGGAGGCGGCGGGGATTCGACTTCCCCTGGGGGTA	32,089	32,161	0	reverse	Glu	UUC	73	7	4	9	5	7	4	5	7
S. williamsii _32967	GCCCCTTTAACTCAGCGGTAGAGTAACGCCATGGTAAGGCGTAAGTCATCGGTTCAAATCCGATAAGGGGCT	32,967	33,038	0	forward	Thr	GGU	72	7	4	7	5	7	5	5	7
S. williamsii _37050	GGAGAGATGGCTGAGTGGTTGATAGCTCCGGTCTTGAAAACCGGTATAGTTTTGAACAAAGAACTATCGAGGGTTCGAATCCCTCTCTCTCCT	37,050	37,142	0	reverse	Ser	UGA	93	7	4	9	5	7	24	5	7
S. williamsii _37984	GCGGATATGGTCGAATGGTAAAATTTCTCTTTGCCAAGGAGAAGACGCGGGTTCGATTCCCGCTATCCGCC	37,984	38,054	0	forward	Gly	GCC	71	7	2	11	5	7	4	5	7
S. williamsii _38226	CGCGGGGTAGAGCAGTTTGGTAGCTCGCAAGGCTCATAACCTTGAGGTCACGGGTTCAAATCCTGTCTCCGCAA	38,226	38,299	0	reverse	fMet	CAU	74	6	4	9	5	7	5	5	7
S. williamsii _46764	GGAGAGATGGCCGAGTGGTTCAAGGCGTAGCATTGGAACTGCTATGTAGGCTTTTGTTTACCGAGGGTTCGAATCCCTCTCTTTCCG	46,764	46,850	0	forward	Ser	GGA	87	7	3	11	5	7	18	5	7
S. williamsii _48092	GCCTGCTTAGCTCAGAGGTTAGAGCATCGCATTTGTAATGCGATGGTCATCGGTTCGATTCCGATAGCCGGCT	48,092	48,164	0	reverse	Thr	UGU	73	7	4	8	5	7	5	5	7
S. williamsii _48956	GGGGATATGGCGAAATTGGTAGACGCTACGGACTTAAAAAATCCGTCGACTTTAGAAATCGTGAGGGTTCAAGTCCCTCTATCCCCA	48,956	49,559	1	forward	Leu	UAA	87	7	3	11	5	9	16	5	7
S. williamsii _49928	GTCGGGATAGCTCAGCTGGTAGAGCAGAGGACTGAAAATCCTCGTGTCACCAGTTCAAATCTGGTTCCTGGCA	49,928	50,000	0	forward	Phe	GAA	73	7	4	8	5	7	5	5	7
S. williamsii _53583	AGGGCTATAGCTCAGTTGGTAGAGCACCTCGTTTACACACCGAGAATGTCTACGGTTCGAGTCCGTATAGCCCTA	53,583	54,232	1	reverse	Val	UAC	75	7	4	8	4	9	6	5	7
S. williamsii _54418	CCTACTTAACTCAGTGGTTAGAGTATTGCTTTCATACGGCGGGAGTCATTGGTTCAAATCCAATAGTAGGTA	54,418	54,489	0	forward	Met	CAU	72	6	4	8	5	7	5	5	7
S. williamsii _68929	GCGCTCTTAGTTCAGTTCGGTAGAACGTGGGTCTCCAAAACCCGATGTCGTAGGTTCAAATCCTACAGAGCGTG	68,929	69,002	0	reverse	Trp	CCA	74	7	4	9	5	7	5	5	7
S. williamsii _69164	AGGGATGTGGCGCAGCTTGGTAGCGCGTTTGTTTTGGGTACAAAATGTCACAGGTTCAAATCCTGTCATCCCTA	69,164	69,237	0	reverse	Pro	UGG	74	7	4	9	5	7	5	5	7
S. williamsii _88952	GCATCCATGGCTGAATGGTTAAAGCGCCCAACTCATAATTGGCGAATTCGTAGGTTCAATTCCTACTGGATGCA	88,952	89,025	0	reverse	Ile2	CAU	74	7	3	10	5	7	5	5	7
S. williamsii _96741	GCCTTGGTGGTGAAATGGTAGACACGCGAGACTCAAAATCTCGTGCTAAAGAGCGTGGAGGTTCGAGTCCTCTTCAAGGCA	96,741	96,821	0	reverse	Leu	CAA	81	7	3	10	5	7	13	5	7
S. williamsii _103115	AGGGATATAACTCAGCGGTAGAGTGTCACCTTGACGTGGTGGAAGTCATCAGTTCGAGCCTGATTATCCCTA	103,115	103,186	0	forward	Val	GAC	72	7	4	7	5	7	5	5	7
S. williamsii _105190	GGGCTATTAGCTCAGTGGTAGAGCGCGCCCCTGATAATTGCGGGGCGAGGTCTCTGGTTCAAGTCCAGGATGGCCCA	105,190	106,208	1	forward	Ile	GAU	77	7	4	7	5	12	5	5	7
S. williamsii _106272	GGGGATATAGCTCAGTTGGTAGAGCTCCGCTCTTGCAAGGCGGATGTCAGCGGTTCGAGTCCGCTTATCTCCA	106,272	107,159	1	forward	Ala	UGC	73	7	4	8	5	7	5	5	7
S. williamsii _110920	GGGCCTGTAGCTCAGAGGATTAGAGCACGTGGCTACGAACCACGGTGTCGGGGGTTCGAATCCCTCCTCGCCCA	110,920	110,993	0	forward	Arg	ACG	74	7	4	9	5	7	5	5	7
S. williamsii _111597	TCCTCAGTAGCTCAGTGGTAGAGCGGTCGGCTGTTAACCGATTGGTCGTAGGTTCGAATCCTACTTGGGGAG	111,597	111,668	0	reverse	Asn	GUU	72	7	4	7	5	7	5	5	7
S. williamsii _117749	GCCGCTATGGTGAAATCGGTAGACACGCTGCTCTTAGGAAGCAGTGCTAGAGCATCTCGGTTCGAGTCCGAGTGGCGGCA	117,749	117,828	0	forward	Leu	UAG	80	7	3	11	5	7	11	5	7
S. williamsii _133448	TCCTCAGTAGCTCAGTGGTAGAGCGGTCGGCTGTTAACCGATTGGTCGTAGGTTCGAATCCTACTTGGGGAG	133,448	133,519	0	forward	Asn	GUU	72	7	4	7	5	7	5	5	7
S. williamsii _134123	GGGCCTGTAGCTCAGAGGATTAGAGCACGTGGCTACGAACCACGGTGTCGGGGGTTCGAATCCCTCCTCGCCCA	134,123	134,196	0	reverse	Arg	ACG	74	7	4	9	5	7	5	5	7
S. williamsii _137957	GGGGATATAGCTCAGTTGGTAGAGCTCCGCTCTTGCAAGGCGGATGTCAGCGGTTCGAGTCCGCTTATCTCCA	137,957	138,844	1	reverse	Ala	UGC	73	7	4	8	5	7	5	5	7
S. williamsii _138908	GGGCTATTAGCTCAGTGGTAGAGCGCGCCCCTGATAATTGCGGGGCGAGGTCTCTGGTTCAAGTCCAGGATGGCCCA	138,908	139,926	1	reverse	Ile	GAU	77	7	4	7	5	12	5	5	7
S. williamsii _141930	AGGGATATAACTCAGCGGTAGAGTGTCACCTTGACGTGGTGGAAGTCATCAGTTCGAGCCTGATTATCCCTA	141,930	142,001	0	reverse	Val	GAC	72	7	4	7	5	7	5	5	7
S. williamsii _148295	GCCTTGGTGGTGAAATGGTAGACACGCGAGACTCAAAATCTCGTGCTAAAGAGCGTGGAGGTTCGAGTCCTCTTCAAGGCA	148,295	148,375	0	forward	Leu	CAA	81	7	3	10	5	7	13	5	7
S. williamsii _156091	GCATCCATGGCTGAATGGTTAAAGCGCCCAACTCATAATTGGCGAATTCGTAGGTTCAATTCCTACTGGATGCA	156,091	156,164	0	forward	Ile2	CAU	74	7	3	10	5	7	5	5	7
V. utile _4	GCGGATGTAGCCAAGTGGATCAAGGCAGTGGATTGTGAATCCACCATGCGCGGGTTCAATTCCCGTCGTTCGCC	4	77	0	reverse	His	GUG	74	7	3	11	5	7	5	5	7
V. utile _1763	GGGTTGCTAACTCAACGGTAGAGTACTCGGCTTTTAACCGACTAGTTCCGGGTTCGAATCCCGGGCAACCCA	1,763	4,358	1	reverse	Lys	UUU	72	7	4	7	4	7	6	5	7
V. utile _7865	TGGGGCGTGGCCAAGTGGTAAGGCAACGGGTTTTGGTCCCGCTATTCGGAGGTTCGAATCCTTCCGTCCCAG	7,865	7,936	0	reverse	Gln	UUG	72	7	3	9	4	7	6	5	7
V. utile _9059	GGAGAGATGGCTGAGTGGACTAAAGCGGCGGATTGCTAATCCGTTGTACGAGTTATTCGTACCGAGGGTTCGAATCCCTCTCTTTCCG	9,059	9,146	0	reverse	Ser	GCU	88	7	3	11	5	7	19	5	7
V. utile _9853	GCGGGTATAGTTTAGTGGTAAAACCTTAGCCTTCCAAGCTAACGATGCGGGTTCGATTCCCGCTACCCGC	9,853	10,621	1	forward	Gly	UCC	70	7	4	7	5	7	4	5	7
V. utile _10799	GCGTCCATTGTCTAATGGATAGGACATAGGTCTTCTAAACCTTTGGTATAGGTTCAAATCCTATTGGACGCA	10,799	10,870	0	forward	Arg	UCU	72	7	4	8	4	7	5	5	7
V. utile _28913	GGCGGCATGGCCGAGTGGTAAGGCGGGGGACTGCAAATCCTTTTTCCCCAGTTCAAATCCGGGTGTCGCCT	28,913	28,983	0	forward	Cys	GCA	71	7	3	9	5	7	4	5	7
V. utile _31685	GGGATTGTAGTTCAATTGGTCAGAGCACCGCCCTGTCAAGGCGGAAGCTGCGGGTTCGAGCCCCGTCAGTCCCG	31,685	31,758	0	reverse	Asp	GUC	74	7	4	9	5	7	5	5	7
V. utile _31868	GGGTCGATGCCCGAGCGGTTAATGGGGACGGACTGTAAATTCGTTGGCAATATGTCTACGCTGGTTCAAATCCAGCTCGGCCCA	31,868	31,951	0	reverse	Tyr	GUA	84	7	4	9	5	7	4	5	7
V. utile _32016	GCCCCCATCGTCTAGTGGTTCAGGACATCTCTCTTTCAAGGAGGCGGCGGGGATTCGACTTCCCCTGGGGGTA	32,016	32,088	0	reverse	Glu	UUC	73	7	4	9	5	7	4	5	7
V. utile _32906	GCCCCTTTAACTCAGCGGTAGAGTAACGCCATGGTAAGGCGTAAGTCATCGGTTCAAATCCGATAAGGGGCT	32,906	32,977	0	forward	Thr	GGU	72	7	4	7	5	7	5	5	7
V. utile _36807	GGAGAGATGGCTGAGTGGTTGATAGCTCCGGTCTTGAAAACCGGTATAGTTTTGAACAAAGAACTATCGAGGGTTCGAATCCCTCTCTCTCCT	36,807	36,899	0	reverse	Ser	UGA	93	7	4	9	5	7	24	5	7
V. utile _37736	GCGGATATGGTCGAATGGTAAAATTTCTCTTTGCCAAGGAGAAGACGCGGGTTCGATTCCCGCTATCCGCC	37,736	37,806	0	forward	Gly	GCC	71	7	2	11	5	7	4	5	7
V. utile _37980	CGCGGGGTAGAGCAGTTTGGTAGCTCGCAAGGCTCATAACCTTGAGGTCACGGGTTCAAATCCTGTCTCCGCAA	37,980	38,053	0	reverse	fMet	CAU	74	6	4	9	5	7	5	5	7
V. utile _46707	GGAGAGATGGCCGAGTGGTTCAAGGCGTAGCATTGGAACTGCTATGTAGGCTTTTGTTTACCGAGGGTTCGAATCCCTCTCTTTCCG	46,707	46,793	0	forward	Ser	GGA	87	7	3	11	5	7	18	5	7
V. utile _48048	GCCTGCTTAGCTCAGAGGTTAGAGCATCGCATTTGTAATGCGATGGTCATCGGTTCGATTCCGATAGCCGGCT	48,048	48,120	0	reverse	Thr	UGU	73	7	4	8	5	7	5	5	7
V. utile _48840	GGGGATATGGCGAAATTGGTAGACGCTACGGACTTAAAAAATCCGTCGACTTTAGAAATCGTGAGGGTTCAAGTCCCTCTATCCCCA	48,840	49,429	1	forward	Leu	UAA	87	7	3	11	5	9	16	5	7
V. utile _49808	GTCGGGATAGCTCAGCCGGTAGAGCAGAGGACTGAAAATCCTCGTGTCACCAGTTCAAATCTGGTTCCTGGCA	49,808	49,880	0	forward	Phe	GAA	73	7	4	8	5	7	5	5	7
V. utile _53289	AGGGCTATAGCTCAGTTAGGTAGAGCACCTCGTTTACACACCGAGAAGGTCTACGGTTCGAGTCCGTATAGCCCTA	53,289	53,938	1	reverse	Val	UAC	76	7	4	9	4	9	6	5	7
V. utile _54115	CCTACTTAACTCAGTGGTTAGAGTATTGCTTTCATACGGCAGGAGTCATTGGTTCAAATCCAATAGTAGGTA	54,115	54,186	0	forward	Met	CAU	72	6	4	8	5	7	5	5	7
V. utile _68623	GCGCTCTTAGTTCAGTTCGGTAGAACGTGGGTCTCCAAAACCCGATGTCGTAGGTTCAAATCCTACAGAGCGTG	68,623	68,696	0	reverse	Trp	CCA	74	7	4	9	5	7	5	5	7
V. utile _68872	AGGGATGTGGCGCAGCTTGGTAGCGCGTTTGTTTTGGGTACAAAATGTCACGGGTTCAAATCCTGTCATCCCTA	68,872	68,945	0	reverse	Pro	UGG	74	7	4	9	5	7	5	5	7
V. utile _88625	GCATCCATGGCTGAATGGTTAAAGCGCCCAACTCATAATTGGCGAATTCGTAGGTTCAATTCCTACTGGATGCA	88,625	88,698	0	reverse	Ile2	CAU	74	7	3	10	5	7	5	5	7
V. utile _96403	GCCTTGGTGGTGAAATGGTAGACACGCGAGACTCAAAATCTCGTGCTAAAGAGCGTGGAGGTTCGAGTCCTCTTCAAGGCA	96,403	96,483	0	reverse	Leu	CAA	81	7	3	10	5	7	13	5	7
V. utile _102789	AGGGATATAACTCAGCGGTAGAGTGTCACCTTGACGTGGTGGAAGTCATCAGTTCGAGCCTGATTATCCCTA	102,789	102,860	0	forward	Val	GAC	72	7	4	7	5	7	5	5	7
V. utile _104863	GGGCTATTAGCTCAGTGGTAGAGCGCGCCCCTGATAATTGCGGGGCGAGGTCTCTGGTTCAAGTCCAGGATGGCCCA	104,863	105,881	1	forward	Ile	GAU	77	7	4	7	5	12	5	5	7
V. utile _105946	GGGGATATAGCTCAGTTGGTAGAGCTCCGCTCTTGCAAGGCGGATGTCAGCGGTTCGAGTCCGCTTATCTCCA	105,946	106,833	1	forward	Ala	UGC	73	7	4	8	5	7	5	5	7
V. utile _110593	GGGCCTGTAGCTCAGAGGATTAGAGCACGTGGCTACGAACCACGGTGTCGGGGGTTCGAATCCCTCCTCGCCCA	110,593	110,666	0	forward	Arg	ACG	74	7	4	9	5	7	5	5	7
V. utile _111279	TCCTCAGTAGCTCAGTGGTAGAGCGGTCGGCTGTTAACCGATTGGTCGTAGGTTCGAATCCTACTTGGGGAG	111,279	111,350	0	reverse	Asn	GUU	72	7	4	7	5	7	5	5	7
V. utile _117229	GCCGCTATGGTGAAATCGGTAGACACGCTGCTCTTAGGAAGCAGTGCTAGAGCATCTCGGTTCGAGTCCGAGTGGCGGCA	117,229	117,308	0	forward	Leu	UAG	80	7	3	11	5	7	11	5	7
V. utile _132850	TCCTCAGTAGCTCAGTGGTAGAGCGGTCGGCTGTTAACCGATTGGTCGTAGGTTCGAATCCTACTTGGGGAG	132,850	132,921	0	forward	Asn	GUU	72	7	4	7	5	7	5	5	7
V. utile _133534	GGGCCTGTAGCTCAGAGGATTAGAGCACGTGGCTACGAACCACGGTGTCGGGGGTTCGAATCCCTCCTCGCCCA	133,534	133,607	0	reverse	Arg	ACG	74	7	4	9	5	7	5	5	7
V. utile _137367	GGGGATATAGCTCAGTTGGTAGAGCTCCGCTCTTGCAAGGCGGATGTCAGCGGTTCGAGTCCGCTTATCTCCA	137,367	138,254	1	reverse	Ala	UGC	73	7	4	8	5	7	5	5	7
V. utile _138319	GGGCTATTAGCTCAGTGGTAGAGCGCGCCCCTGATAATTGCGGGGCGAGGTCTCTGGTTCAAGTCCAGGATGGCCCA	138,319	139,337	1	reverse	Ile	GAU	77	7	4	7	5	12	5	5	7
V. utile _141340	AGGGATATAACTCAGCGGTAGAGTGTCACCTTGACGTGGTGGAAGTCATCAGTTCGAGCCTGATTATCCCTA	141,340	141,411	0	reverse	Val	GAC	72	7	4	7	5	7	5	5	7
V. utile _147717	GCCTTGGTGGTGAAATGGTAGACACGCGAGACTCAAAATCTCGTGCTAAAGAGCGTGGAGGTTCGAGTCCTCTTCAAGGCA	147,717	147,797	0	forward	Leu	CAA	81	7	3	10	5	7	13	5	7
V. utile _155502	GCATCCATGGCTGAATGGTTAAAGCGCCCAACTCATAATTGGCGAATTCGTAGGTTCAATTCCTACTGGATGCA	155,502	155,575	0	forward	Ile2	CAU	74	7	3	10	5	7	5	5	7
V. betulifolium _4	GCGGATGTAGCCAAGTGGATCAAGGCAGTGGATTGTGAATCCACCATGCGCGGGTTCAATTCCCGTCGTTCGCC	4	77	0	reverse	His	GUG	74	7	3	11	5	7	5	5	7
V. betulifolium _1761	GGGTTGCTAACTCAACGGTAGAGTACTCGGCTTTTAACCGACTAGTTCCGGGTTCGAATCCCGGGCAACCCA	1761	4357	1	reverse	Lys	UUU	72	7	4	7	4	7	6	5	7
V. betulifolium _7872	TGGGGCGTAGCCAAGTGGTAAGGCAACGGGTTTTGGTCCCGCTATTCGGAGGTTCGAATCCTTCCGTCCCAG	7872	7943	0	reverse	Gln	UUG	72	7	3	9	4	7	6	5	7
V. betulifolium _9056	GGAGAGATGGCTGAGTGGACTAAAGCGGCGGATTGCTAATCCGTTGTACGAGTTATTCGTACCGAGGGTTCGAATCCCTCTCTTTCCG	9056	9143	0	reverse	Ser	GCU	88	7	3	11	5	7	19	5	7
V. betulifolium _9849	GCGGGTATAGTTTAGTGGTAAAACCTTAGCCTTCCAAGCTAACGATGCGGGTTCGATTCCCGCTACCCGC	9849	10617	1	forward	Gly	UCC	70	7	4	7	5	7	4	5	7
V. betulifolium _10792	GCGTCCATTGTCTAATGGATAGGACATAGGTCTTCTAAACCTTTGGTATAGGTTCAAATCCTATTGGACGCA	10792	10863	0	forward	Arg	UCU	72	7	4	8	4	7	5	5	7
V. betulifolium _28919	GGCGGCATGGCCGAGTGGTAAGGCGGGGGACTGCAAATCCTTTTTCCCCAGTTCAAATCTTTGTGTCGCCC	28919	28989	0	forward	Cys	GCA	71	7	3	9	5	7	4	5	7
V. betulifolium _31727	GGGATTGTAGTTCAATTGGTCAGAGCACCGCCCTGTCAAGGCGGAAGCTGCGGGTTCGAGCCCCGTCAGTCCCG	31727	31800	0	reverse	Asp	GUC	74	7	4	9	5	7	5	5	7
V. betulifolium _31918	GGGTCGATGCCCGAGCGGTTAATGGGGACGGACTGTAAATTCGTTGGCAATATGTCTACGCTGGTTCAAATCCAGCTCGGCCCA	31918	32001	0	reverse	Tyr	GUA	84	7	4	9	5	7	4	5	7
V. betulifolium _32066	GCCCCCATCGTCTAGTGGTTCAGGACATCTCTCTTTCAAGGAGGCGGCGGGGATTCGACTTCCCCTGGGGGTA	32066	32138	0	reverse	Glu	UUC	73	7	4	9	5	7	4	5	7
V. betulifolium _32997	GCCCCTTTAACTCAGCGGTAGAGTAACGCCATGGTAAGGCGTAAGTCATCGGTTCAAATCCGATAAGGGGCT	32997	33068	0	forward	Thr	GGU	72	7	4	7	5	7	5	5	7
V. betulifolium _36932	GGAGAGATGGCTGAGTGGTTGATAGCTCCGGTCTTGAAAACCGGTATAGTTTTGAACAAAGAACTATCGAGGGTTCGAATCCCTCTCTCTCCT	36932	37024	0	reverse	Ser	UGA	93	7	4	9	5	7	24	5	7
V. betulifolium _37859	GCGGATATGGTCGAATGGTAAAATTTCTCTTTGCCAAGGAGAAGACGCGGGTTCGATTCCCGCTATCCGCC	37859	37929	0	forward	Gly	GCC	71	7	2	11	5	7	4	5	7
V. betulifolium _38103	CGCGGGGTAGAGCAGTTTGGTAGCTCGCAAGGCTCATAACCTTGAGGTCACGGGTTCAAATCCTGTCTCCGCAA	38103	38176	0	reverse	fMet	CAU	74	6	4	9	5	7	5	5	7
V. betulifolium _46852	GGAGAGATGGCCGAGTGGTTCAAGGCGTAGCATTGGAACTGCTATGTAGGCTTTTGTTTACCGAGGGTTCGAATCCCTCTCTTTCCG	46852	46938	0	forward	Ser	GGA	87	7	3	11	5	7	18	5	7
V. betulifolium _48200	GCCTGCTTAGCTCAGAGGTTAGAGCATCGCATTTGTAATGCGATGGTCATCGGTTCGATTCCGATAGCCGGCT	48200	48272	0	reverse	Thr	UGU	73	7	4	8	5	7	5	5	7
V. betulifolium _48992	GGGGATATGGCGAAATTGGTAGACGCTACGGACTTAAAAAATCCGTCGACTTTAGAAATCGTGAGGGTTCAAGTCCCTCTATCCCCA	48992	49577	1	forward	Leu	UAA	87	7	3	11	5	9	16	5	7
V. betulifolium _49955	GTCGGGATAGCTCAGCCGGTAGAGCAGAGGACTGAAAATCCTCGTGTCACCAGTTCAAATCTGGTTCCTGGCA	49955	50027	0	forward	Phe	GAA	73	7	4	8	5	7	5	5	7
V. betulifolium _53442	AGGGCTATAGCTCAGTTAGGTAGAGCACCTCGTTTACACACCGAGAAGGTCTACGGTTCGAGTCCGTATAGCCCTA	53442	54091	1	reverse	Val	UAC	76	7	4	9	4	9	6	5	7
V. betulifolium _54268	CCTACTTAACTCAGTGGTTAGAGTATTGCTTTCATACGGCAGGAGTCATTGGTTCAAATCCAATAGTAGGTA	54268	54339	0	forward	Met	CAU	72	6	4	8	5	7	5	5	7
V. betulifolium _68793	GCGCTCTTAGTTCAGTTCGGTAGAACGTGGGTCTCCAAAACCCGATGTCGTAGGTTCAAATCCTACAGAGCGTG	68793	68866	0	reverse	Trp	CCA	74	7	4	9	5	7	5	5	7
V. betulifolium _69060	AGGGATGTGGCGCAGCTTGGTAGCGCGTTTGTTTTGGGTACAAAATGTCACGGGTTCAAATCCTGTCATCCCTA	69060	69133	0	reverse	Pro	UGG	74	7	4	9	5	7	5	5	7
V. betulifolium _88807	GCATCCATGGCTGAATGGTTAAAGCGCCCAACTCATAATTGGCGAATTCGTAGGTTCAATTCCTACTGGATGCA	88807	88880	0	reverse	Ile2	CAU	74	7	3	10	5	7	5	5	7
V. betulifolium _96579	GCCTTGGTGGTGAAATGGTAGACACGCGAGACTCAAAATCTCGTGCTAAAGAGCGTGGAGGTTCGAGTCCTCTTCAAGGCA	96579	96659	0	reverse	Leu	CAA	81	7	3	10	5	7	13	5	7
V. betulifolium _102950	AGGGATATAACTCAGCGGTAGAGTGTCACCTTGACGTGGTGGAAGTCATCAGTTCGAGCCTGATTATCCCTA	102950	103021	0	forward	Val	GAC	72	7	4	7	5	7	5	5	7
V. betulifolium _105024	GGGCTATTAGCTCAGTGGTAGAGCGCGCCCCTGATAATTGCGGGGCGAGGTCTCTGGTTCAAGTCCAGGATGGCCCA	105024	106042	1	forward	Ile	GAU	77	7	4	7	5	12	5	5	7
V. betulifolium _106107	GGGGATATAGCTCAGTTGGTAGAGCTCCGCTCTTGCAAGGCGGATGTCAGCGGTTCGAGTCCGCTTATCTCCA	106107	106994	1	forward	Ala	UGC	73	7	4	8	5	7	5	5	7
V. betulifolium _110759	GGGCCTGTAGCTCAGAGGATTAGAGCACGTGGCTACGAACCACGGTGTCGGGGGTTCGAATCCCTCCTCGCCCA	110759	110832	0	forward	Arg	ACG	74	7	4	9	5	7	5	5	7
V. betulifolium _111443	TCCTCAGTAGCTCAGTGGTAGAGCGGTCGGCTGTTAACCGATTGGTCGTAGGTTCGAATCCTACTTGGGGAG	111443	111514	0	reverse	Asn	GUU	72	7	4	7	5	7	5	5	7
V. betulifolium _117658	GCCGCTATGGTGAAATCGGTAGACACGCTGCTCTTAGGAAGCAGTGCTAGAGCATCTCGGTTCGAGTCCGAGTGGCGGCA	117658	117737	0	forward	Leu	UAG	80	7	3	11	5	7	11	5	7
V. betulifolium _133271	TCCTCAGTAGCTCAGTGGTAGAGCGGTCGGCTGTTAACCGATTGGTCGTAGGTTCGAATCCTACTTGGGGAG	133271	133342	0	forward	Asn	GUU	72	7	4	7	5	7	5	5	7
V. betulifolium _133953	GGGCCTGTAGCTCAGAGGATTAGAGCACGTGGCTACGAACCACGGTGTCGGGGGTTCGAATCCCTCCTCGCCCA	133953	134026	0	reverse	Arg	ACG	74	7	4	9	5	7	5	5	7
V. betulifolium _137791	GGGGATATAGCTCAGTTGGTAGAGCTCCGCTCTTGCAAGGCGGATGTCAGCGGTTCGAGTCCGCTTATCTCCA	137791	138678	1	reverse	Ala	UGC	73	7	4	8	5	7	5	5	7
V. betulifolium _138743	GGGCTATTAGCTCAGTGGTAGAGCGCGCCCCTGATAATTGCGGGGCGAGGTCTCTGGTTCAAGTCCAGGATGGCCCA	138743	139761	1	reverse	Ile	GAU	77	7	4	7	5	12	5	5	7
V. betulifolium _141764	AGGGATATAACTCAGCGGTAGAGTGTCACCTTGACGTGGTGGAAGTCATCAGTTCGAGCCTGATTATCCCTA	141764	141835	0	reverse	Val	GAC	72	7	4	7	5	7	5	5	7
V. betulifolium _148126	GCCTTGGTGGTGAAATGGTAGACACGCGAGACTCAAAATCTCGTGCTAAAGAGCGTGGAGGTTCGAGTCCTCTTCAAGGCA	148126	148206	0	forward	Leu	CAA	81	7	3	10	5	7	13	5	7
V. betulifolium _155905	GCATCCATGGCTGAATGGTTAAAGCGCCCAACTCATAATTGGCGAATTCGTAGGTTCAATTCCTACTGGATGCA	155905	155978	0	forward	Ile2	CAU	74	7	3	10	5	7	5	5	7
V. japonicum _1	GCGGATGTAGCCAAGTGGATCAAGGCAGTGGATTGTGAATCCACCATGCGCGGGTTCAATTCCCGTCGTTCGCC	1	74	0	reverse	His	GUG	74	7	3	11	5	7	5	5	7
V. japonicum _1754	GGGTTGCTAACTCAACGGTAGAGTACTCGGCTTTTAACCGACTAGTTCCGGGTTCGAATCCCGGGCAACCCA	1754	4350	1	reverse	Lys	UUU	72	7	4	7	4	7	6	5	7
V. japonicum _7860	TGGGGCGTAGCCAAGTGGTAAGGCAACGGGTTTTGGTCCCGCTATTCGGAGGTTCGAATCCTTCCGTCCCAG	7860	7931	0	reverse	Gln	UUG	72	7	3	9	4	7	6	5	7
V. japonicum _9054	GGAGAGATGGCTGAGTGGACTAAAGCGGCGGATTGCTAATCCGTTGTACGAGTTATTCGTACCGAGGGTTCGAATCCCTCTCTTTCCG	9054	9141	0	reverse	Ser	GCU	88	7	3	11	5	7	19	5	7
V. japonicum _9850	GCGGGTATAGTTTAGTGGTAAAACCTTAGCCTTCCAAGCTAACGATGCGGGTTCGATTCCCGCTACCCGC	9850	10618	1	forward	Gly	UCC	70	7	4	7	5	7	4	5	7
V. japonicum _10796	GCGTCCATTGTCTAATGGATAGGACATAGGTCTTCTAAACCTTTGGTATAGGTTCAAATCCTATTGGACGCA	10796	10867	0	forward	Arg	UCU	72	7	4	8	4	7	5	5	7
V. japonicum _29149	GGCGGCATGGCCGAGTGGTAAGGCGGGGGACTGCAAATCCTTTTTCCCCAGTTCAAATCCGGGTGTCGCCT	29149	29219	0	forward	Cys	GCA	71	7	3	9	5	7	4	5	7
V. japonicum _32257	GGGATTGTAGTTCAATTGGTCAGAGCACCGCCCTGTCAAGGCGGAAGCTGCGGGTTCGAGCCCCGTCAGTCCCG	32257	32330	0	reverse	Asp	GUC	74	7	4	9	5	7	5	5	7
V. japonicum _32443	GGGTCGATGCCCGAGCGGTTAATGGGGACGGACTGTAAATTCGTTGGCAATATGTCTACGCTGGTTCAAATCCAGCTCGGCCCA	32443	32526	0	reverse	Tyr	GUA	84	7	4	9	5	7	4	5	7
V. japonicum _32591	GCCCCCATCGTCTAGTGGTTCAGGACATCTCTCTTTCAAGGAGGCGGCGGGGATTCGACTTCCCCTGGGGGTA	32591	32663	0	reverse	Glu	UUC	73	7	4	9	5	7	4	5	7
V. japonicum _33476	GCCCCTTTAACTCAGCGGTAGAGTAACGCCATGGTAAGGCGTAAGTCATCGGTTCAAATCCGATAAGGGGCT	33476	33547	0	forward	Thr	GGU	72	7	4	7	5	7	5	5	7
V. japonicum _37349	GGAGAGATGGCTGAGTGGTTGATAGCTCCGGTCTTGAAAACCGGTATAGTTTTGAACAAAGAACTATCGAGGGTTCGAATCCCTCTCTCTCCT	37349	37441	0	reverse	Ser	UGA	93	7	4	9	5	7	24	5	7
V. japonicum _38268	GCGGATATGGTCGAATGGTAAAATTTCTCTTTGCCAAGGAGAAGACGCGGGTTCGATTCCCGCTATCCGCC	38268	38338	0	forward	Gly	GCC	71	7	2	11	5	7	4	5	7
V. japonicum _38512	CGCGGGGTAGAGCAGTTTGGTAGCTCGCAAGGCTCATAACCTTGAGGTCACGGGTTCAAATCCTGTCTCCGCAA	38512	38585	0	reverse	fMet	CAU	74	6	4	9	5	7	5	5	7
V. japonicum _47250	GGAGAGATGGCCGAGTGGTTCAAGGCGTAGCATTGGAACTGCTATGTAGGCTTTTGTTTACCGAGGGTTCGAATCCCTCTCTTTCCG	47250	47336	0	forward	Ser	GGA	87	7	3	11	5	7	18	5	7
V. japonicum _48617	GCCTGCTTAGCTCAGAGGTTAGAGCATCGCATTTGTAATGCGATGGTCATCGGTTCGATTCCGATAGCCGGCT	48617	48689	0	reverse	Thr	UGU	73	7	4	8	5	7	5	5	7
V. japonicum _49409	GGGGATATGGCGAAATTGGTAGACGCTACGGACTTAAAAAATCCGTCGACTTTAGAAATCGTGAGGGTTCAAGTCCCTCTATCCCCA	49409	49995	1	forward	Leu	UAA	87	7	3	11	5	9	16	5	7
V. japonicum _50373	GTCGGGATAGCTCAGCCGGTAGAGCAGAGGACTGAAAATCCTCGTGTCACCAGTTCAAATCTGGTTCCTGGCA	50373	50445	0	forward	Phe	GAA	73	7	4	8	5	7	5	5	7
V. japonicum _53868	AGGGCTATAGCTCAGTTAGGTAGAGCACCTCGTTTACACACCGAGAAGGTCTACGGTTCGAGTCCGTATAGCCCTA	53868	54517	1	reverse	Val	UAC	76	7	4	9	4	9	6	5	7
V. japonicum _54694	CCTACTTAACTCAGTGGTTAGAGTATTGCTTTCATACGGCAGGAGTCATTGGTTCAAATCCAATAGTAGGTA	54694	54765	0	forward	Met	CAU	72	6	4	8	5	7	5	5	7
V. japonicum _69128	GCGCTCTTAGTTCAGTTCGGTAGAACGTGGGTCTCCAAAACCCGATGTCGTAGGTTCAAATCCTACAGAGCGTG	69128	69201	0	reverse	Trp	CCA	74	7	4	9	5	7	5	5	7
V. japonicum _69395	AGGGATGTGGCGCAGCTTGGTAGCGCGTTTGTTTTGGGTACAAAATGTCACGGGTTCAAATCCTGTCATCCCTA	69395	69468	0	reverse	Pro	UGG	74	7	4	9	5	7	5	5	7
V. japonicum _89107	GCATCCATGGCTGAATGGTTAAAGCGCCCAACTCATAATTGGCGAATTCGTAGGTTCAATTCCTACTGGATGCA	89107	89180	0	reverse	Ile2	CAU	74	7	3	10	5	7	5	5	7
V. japonicum _96885	GCCTTGGTGGTGAAATGGTAGACACGCGAGACTCAAAATCTCGTGCTAAAGAGCGTGGAGGTTCGAGTCCTCTTCAAGGCA	96885	96965	0	reverse	Leu	CAA	81	7	3	10	5	7	13	5	7
V. japonicum _103262	AGGGATATAACTCAGCGGTAGAGTGTCACCTTGACGTGGTGGAAGTCATCAGTTCGAGCCTGATTATCCCTA	103262	103333	0	forward	Val	GAC	72	7	4	7	5	7	5	5	7
V. japonicum _105336	GGGCTATTAGCTCAGTGGTAGAGCGCGCCCCTGATAATTGCGGGGCGAGGTCTCTGGTTCAAGTCCAGGATGGCCCA	105336	106354	1	forward	Ile	GAU	77	7	4	7	5	12	5	5	7
V. japonicum _106419	GGGGATATAGCTCAGTTGGTAGAGCTCCGCTCTTGCAAGGCGGATGTCAGCGGTTCGAGTCCGCTTATCTCCA	106419	107306	1	forward	Ala	UGC	73	7	4	8	5	7	5	5	7
V. japonicum _111098	GGGCCTGTAGCTCAGAGGATTAGAGCACGTGGCTACGAACCACGGTGTCGGGGGTTCGAATCCCTCCTCGCCCA	111098	111171	0	forward	Arg	ACG	74	7	4	9	5	7	5	5	7
V. japonicum _111781	TCCTCAGTAGCTCAGTGGTAGAGCGGTCGGCTGTTAACCGATTGGTCGTAGGTTCGAATCCTACTTGGGGAG	111781	111852	0	reverse	Asn	GUU	72	7	4	7	5	7	5	5	7
V. japonicum _118136	GCCGCTATGGTGAAATCGGTAGACACGCTGCTCTTAGGAAGCAGTGCTAGAGCATCTCGGTTCGAGTCCGAGTGGCGGCA	118136	118215	0	forward	Leu	UAG	80	7	3	11	5	7	11	5	7
V. japonicum _133822	TCCTCAGTAGCTCAGTGGTAGAGCGGTCGGCTGTTAACCGATTGGTCGTAGGTTCGAATCCTACTTGGGGAG	133822	133893	0	forward	Asn	GUU	72	7	4	7	5	7	5	5	7
V. japonicum _134503	GGGCCTGTAGCTCAGAGGATTAGAGCACGTGGCTACGAACCACGGTGTCGGGGGTTCGAATCCCTCCTCGCCCA	134503	134576	0	reverse	Arg	ACG	74	7	4	9	5	7	5	5	7
V. japonicum _138368	GGGGATATAGCTCAGTTGGTAGAGCTCCGCTCTTGCAAGGCGGATGTCAGCGGTTCGAGTCCGCTTATCTCCA	138368	139255	1	reverse	Ala	UGC	73	7	4	8	5	7	5	5	7
V. japonicum _139320	GGGCTATTAGCTCAGTGGTAGAGCGCGCCCCTGATAATTGCGGGGCGAGGTCTCTGGTTCAAGTCCAGGATGGCCCA	139320	140338	1	reverse	Ile	GAU	77	7	4	7	5	12	5	5	7
V. japonicum _142341	AGGGATATAACTCAGCGGTAGAGTGTCACCTTGACGTGGTGGAAGTCATCAGTTCGAGCCTGATTATCCCTA	142341	142412	0	reverse	Val	GAC	72	7	4	7	5	7	5	5	7
V. japonicum _148709	GCCTTGGTGGTGAAATGGTAGACACGCGAGACTCAAAATCTCGTGCTAAAGAGCGTGGAGGTTCGAGTCCTCTTCAAGGCA	148709	148789	0	forward	Leu	CAA	81	7	3	10	5	7	13	5	7
V. japonicum _156494	GCATCCATGGCTGAATGGTTAAAGCGCCCAACTCATAATTGGCGAATTCGTAGGTTCAATTCCTACTGGATGCA	156494	156567	0	forward	Ile2	CAU	74	7	3	10	5	7	5	5	7
V. erosum _3	GCGGATGTAGCCAAGTGGATCAAGGCAGTGGATTGTGAATCCACCATGCGCGGGTTCAATTCCCGTCGTTCGCC	3	76	0	reverse	His	GUG	74	7	3	11	5	7	5	5	7
V. erosum _1756	GGGTTGCTAACTCAACGGTAGAGTACTCGGCTTTTAACCGACTAGTTCCGGGTTCGAATCCCGGGCAACCCA	1756	4352	1	reverse	Lys	UUU	72	7	4	7	4	7	6	5	7
V. erosum _7862	TGGGGCGTAGCCAAGTGGTAAGGCAACGGGTTTTGGTCCCGCTATTCGGAGGTTCGAATCCTTCCGTCCCAG	7862	7933	0	reverse	Gln	UUG	72	7	3	9	4	7	6	5	7
V. erosum _9056	GGAGAGATGGCTGAGTGGACTAAAGCGGCGGATTGCTAATCCGTTGTACGAGTTATTCGTACCGAGGGTTCGAATCCCTCTCTTTCCG	9056	9143	0	reverse	Ser	GCU	88	7	3	11	5	7	19	5	7
V. erosum _9852	GCGGGTATAGTTTAGTGGTAAAACCTTAGCCTTCCAAGCTAACGATGCGGGTTCGATTCCCGCTACCCGC	9852	10620	1	forward	Gly	UCC	70	7	4	7	5	7	4	5	7
V. erosum _10798	GCGTCCATTGTCTAATGGATAGGACATAGGTCTTCTAAACCTTTGGTATAGGTTCAAATCCTATTGGACGCA	10798	10869	0	forward	Arg	UCU	72	7	4	8	4	7	5	5	7
V. erosum _29151	GGCGGCATGGCCGAGTGGTAAGGCGGGGGACTGCAAATCCTTTTTCCCCAGTTCAAATCCGGGTGTCGCCT	29151	29221	0	forward	Cys	GCA	71	7	3	9	5	7	4	5	7
V. erosum _32259	GGGATTGTAGTTCAATTGGTCAGAGCACCGCCCTGTCAAGGCGGAAGCTGCGGGTTCGAGCCCCGTCAGTCCCG	32259	32332	0	reverse	Asp	GUC	74	7	4	9	5	7	5	5	7
V. erosum _32445	GGGTCGATGCCCGAGCGGTTAATGGGGACGGACTGTAAATTCGTTGGCAATATGTCTACGCTGGTTCAAATCCAGCTCGGCCCA	32445	32528	0	reverse	Tyr	GUA	84	7	4	9	5	7	4	5	7
V. erosum _32593	GCCCCCATCGTCTAGTGGTTCAGGACATCTCTCTTTCAAGGAGGCGGCGGGGATTCGACTTCCCCTGGGGGTA	32593	32665	0	reverse	Glu	UUC	73	7	4	9	5	7	4	5	7
V. erosum _33478	GCCCCTTTAACTCAGCGGTAGAGTAACGCCATGGTAAGGCGTAAGTCATCGGTTCAAATCCGATAAGGGGCT	33478	33549	0	forward	Thr	GGU	72	7	4	7	5	7	5	5	7
V. erosum _37352	GGAGAGATGGCTGAGTGGTTGATAGCTCCGGTCTTGAAAACCGGTATAGTTTTGAACAAAGAACTATCGAGGGTTCGAATCCCTCTCTCTCCT	37352	37444	0	reverse	Ser	UGA	93	7	4	9	5	7	24	5	7
V. erosum _38270	GCGGATATGGTCGAATGGTAAAATTTCTCTTTGCCAAGGAGAAGACGCGGGTTCGATTCCCGCTATCCGCC	38270	38340	0	forward	Gly	GCC	71	7	2	11	5	7	4	5	7
V. erosum _38514	CGCGGGGTAGAGCAGTTTGGTAGCTCGCAAGGCTCATAACCTTGAGGTCACGGGTTCAAATCCTGTCTCCGCAA	38514	38587	0	reverse	fMet	CAU	74	6	4	9	5	7	5	5	7
V. erosum _47252	GGAGAGATGGCCGAGTGGTTCAAGGCGTAGCATTGGAACTGCTATGTAGGCTTTTGTTTACCGAGGGTTCGAATCCCTCTCTTTCCG	47252	47338	0	forward	Ser	GGA	87	7	3	11	5	7	18	5	7
V. erosum _48619	GCCTGCTTAGCTCAGAGGTTAGAGCATCGCATTTGTAATGCGATGGTCATCGGTTCGATTCCGATAGCCGGCT	48619	48691	0	reverse	Thr	UGU	73	7	4	8	5	7	5	5	7
V. erosum _49411	GGGGATATGGCGAAATTGGTAGACGCTACGGACTTAAAAAATCCGTCGACTTTAGAAATCGTGAGGGTTCAAGTCCCTCTATCCCCA	49411	49997	1	forward	Leu	UAA	87	7	3	11	5	9	16	5	7
V. erosum _50375	GTCGGGATAGCTCAGCCGGTAGAGCAGAGGACTGAAAATCCTCGTGTCACCAGTTCAAATCTGGTTCCTGGCA	50375	50447	0	forward	Phe	GAA	73	7	4	8	5	7	5	5	7
V. erosum _53870	AGGGCTATAGCTCAGTTAGGTAGAGCACCTCGTTTACACACCGAGAAGGTCTACGGTTCGAGTCCGTATAGCCCTA	53870	54519	1	reverse	Val	UAC	76	7	4	9	4	9	6	5	7
V. erosum _54696	CCTACTTAACTCAGTGGTTAGAGTATTGCTTTCATACGGCAGGAGTCATTGGTTCAAATCCAATAGTAGGTA	54696	54767	0	forward	Met	CAU	72	6	4	8	5	7	5	5	7
V. erosum _69130	GCGCTCTTAGTTCAGTTCGGTAGAACGTGGGTCTCCAAAACCCGATGTCGTAGGTTCAAATCCTACAGAGCGTG	69130	69203	0	reverse	Trp	CCA	74	7	4	9	5	7	5	5	7
V. erosum _69397	AGGGATGTGGCGCAGCTTGGTAGCGCGTTTGTTTTGGGTACAAAATGTCACGGGTTCAAATCCTGTCATCCCTA	69397	69470	0	reverse	Pro	UGG	74	7	4	9	5	7	5	5	7
V. erosum _89100	GCATCCATGGCTGAATGGTTAAAGCGCCCAACTCATAATTGGCGAATTCGTAGGTTCAATTCCTACTGGATGCA	89100	89173	0	reverse	Ile2	CAU	74	7	3	10	5	7	5	5	7
V. erosum _96878	GCCTTGGTGGTGAAATGGTAGACACGCGAGACTCAAAATCTCGTGCTAAAGAGCGTGGAGGTTCGAGTCCTCTTCAAGGCA	96878	96958	0	reverse	Leu	CAA	81	7	3	10	5	7	13	5	7
V. erosum _103255	AGGGATATAACTCAGCGGTAGAGTGTCACCTTGACGTGGTGGAAGTCATCAGTTCGAGCCTGATTATCCCTA	103255	103326	0	forward	Val	GAC	72	7	4	7	5	7	5	5	7
V. erosum _105329	GGGCTATTAGCTCAGTGGTAGAGCGCGCCCCTGATAATTGCGGGGCGAGGTCTCTGGTTCAAGTCCAGGATGGCCCA	105329	106347	1	forward	Ile	GAU	77	7	4	7	5	12	5	5	7
V. erosum _106412	GGGGATATAGCTCAGTTGGTAGAGCTCCGCTCTTGCAAGGCGGATGTCAGCGGTTCGAGTCCGCTTATCTCCA	106412	107299	1	forward	Ala	UGC	73	7	4	8	5	7	5	5	7
V. erosum _111091	GGGCCTGTAGCTCAGAGGATTAGAGCACGTGGCTACGAACCACGGTGTCGGGGGTTCGAATCCCTCCTCGCCCA	111091	111164	0	forward	Arg	ACG	74	7	4	9	5	7	5	5	7
V. erosum _111775	TCCTCAGTAGCTCAGTGGTAGAGCGGTCGGCTGTTAACCGATTGGTCGTAGGTTCGAATCCTACTTGGGGAG	111775	111846	0	reverse	Asn	GUU	72	7	4	7	5	7	5	5	7
V. erosum _118109	GCCGCTATGGTGAAATCGGTAGACACGCTGCTCTTAGGAAGCAGTGCTAGAGCATCTCGGTTCGAGTCCGAGTGGCGGCA	118109	118188	0	forward	Leu	UAG	80	7	3	11	5	7	11	5	7
V. erosum _133796	TCCTCAGTAGCTCAGTGGTAGAGCGGTCGGCTGTTAACCGATTGGTCGTAGGTTCGAATCCTACTTGGGGAG	133796	133867	0	forward	Asn	GUU	72	7	4	7	5	7	5	5	7
V. erosum _134478	GGGCCTGTAGCTCAGAGGATTAGAGCACGTGGCTACGAACCACGGTGTCGGGGGTTCGAATCCCTCCTCGCCCA	134478	134551	0	reverse	Arg	ACG	74	7	4	9	5	7	5	5	7
V. erosum _138343	GGGGATATAGCTCAGTTGGTAGAGCTCCGCTCTTGCAAGGCGGATGTCAGCGGTTCGAGTCCGCTTATCTCCA	138343	139230	1	reverse	Ala	UGC	73	7	4	8	5	7	5	5	7
V. erosum _139295	GGGCTATTAGCTCAGTGGTAGAGCGCGCCCCTGATAATTGCGGGGCGAGGTCTCTGGTTCAAGTCCAGGATGGCCCA	139295	140313	1	reverse	Ile	GAU	77	7	4	7	5	12	5	5	7
V. erosum _142316	AGGGATATAACTCAGCGGTAGAGTGTCACCTTGACGTGGTGGAAGTCATCAGTTCGAGCCTGATTATCCCTA	142316	142387	0	reverse	Val	GAC	72	7	4	7	5	7	5	5	7
V. erosum _148684	GCCTTGGTGGTGAAATGGTAGACACGCGAGACTCAAAATCTCGTGCTAAAGAGCGTGGAGGTTCGAGTCCTCTTCAAGGCA	148684	148764	0	forward	Leu	CAA	81	7	3	10	5	7	13	5	7
V. erosum _156469	GCATCCATGGCTGAATGGTTAAAGCGCCCAACTCATAATTGGCGAATTCGTAGGTTCAATTCCTACTGGATGCA	156469	156542	0	forward	Ile2	CAU	74	7	3	10	5	7	5	5	7
V. carlesii × *V. macrocephalum* _1	GCGGATGTAGCCAAGTGGATCAAGGCAGTGGATTGTGAATCCACCATGCGCGGGTTCAATTCCCGTCGTTCGCC	1	75	0	reverse	His	GUG	75	7	3	11	5	7	5	5	7
V. carlesii × *V. macrocephalum* _1763	GGGTTGCTAACTCAACGGTAGAGTACTCGGCTTTTAACCGACTAGTTCCGGGTTCGAATCCCGGGCAACCCA	1763	4359	1	reverse	Lys	UUU	72	7	4	7	4	7	6	5	7
V. carlesii × *V. macrocephalum* _7865	TGGGGCGTAGCCAAGTGGTAAGGCAACGGGTTTTGGTCCCGCTATTCGGAGGTTCGAATCCTTCCGTCCCAG	7865	7936	0	reverse	Gln	UUG	72	7	3	9	4	7	6	5	7
V. carlesii × *V. macrocephalum* _9058	GGAGAGATGGCTGAGTGGACTAAAGCGGCGGATTGCTAATCCGTTGTACGAGTTATTCGTACCGAGGGTTCGAATCCCTCTCTTTCCG	9058	9145	0	reverse	Ser	GCU	88	7	3	11	5	7	19	5	7
V. carlesii × *V. macrocephalum* _9852	GCGGGTATAGTTTAGTGGTAAAACCTTAGCCTTCCAAGCTAACGATGCGGGTTCGATTCCCGCTACCCGC	9852	10619	1	forward	Gly	UCC	70	7	4	7	5	7	4	5	7
V. carlesii × *V. macrocephalum* _10797	GCGTCCATTGTCTAATGGATAGGACATAGGTCTTCTAAACCTTTGGTATAGGTTCAAATCCTATTGGACGCA	10797	10868	0	forward	Arg	UCU	72	7	4	8	4	7	5	5	7
V. carlesii × *V. macrocephalum* _29127	GGCGGCATGGCCGAGTGGTAAGGCGGGGGACTGCAAATCCTTTTTCCCCAGTTCAAATCCGGGTGTCGCCT	29127	29197	0	forward	Cys	GCA	71	7	3	9	5	7	4	5	7
V. carlesii × *V. macrocephalum* _32550	GGGATTGTAGTTCAATTGGTCAGAGCACCGCCCTGTCAAGGCGGAAGCTGCGGGTTCGAGCCCCGTCAGTCCCG	32550	32623	0	reverse	Asp	GUC	74	7	4	9	5	7	5	5	7
V. carlesii × *V. macrocephalum* _32733	GGGTCGATGCCCGAGCGGTTAATGGGGACGGACTGTAAATTCGTTGGCAATATGTCTACGCTGGTTCAAATCCAGCTCGGCCCA	32733	32816	0	reverse	Tyr	GUA	84	7	4	9	5	7	4	5	7
V. carlesii × *V. macrocephalum* _32881	GCCCCCATCGTCTAGTGGTTCAGGACATCTCTCTTTCAAGGAGGCGGCGGGGATTCGACTTCCCCTGGGGGTA	32881	32953	0	reverse	Glu	UUC	73	7	4	9	5	7	4	5	7
V. carlesii × *V. macrocephalum* _33775	GCCCCTTTAACTCAGCGGTAGAGTAACGCCATGGTAAGGCGTAAGTCATCGGTTCAAATCCGATAAGGGGCT	33775	33846	0	forward	Thr	GGU	72	7	4	7	5	7	5	5	7
V. carlesii × *V. macrocephalum* _37676	GGAGAGATGGCTGAGTGGTTGATAGCTCCGGTCTTGAAAACCGGTATAGTTTTGAACAAAGAACTATCGAGGGTTCGAATCCCTCTCTCTCCT	37676	37768	0	reverse	Ser	UGA	93	7	4	9	5	7	24	5	7
V. carlesii × *V. macrocephalum* _38605	GCGGATATGGTCGAATGGTAAAATTTCTCTTTGCCAAGGAGAAGACGCGGGTTCGATTCCCGCTATCCGCC	38605	38675	0	forward	Gly	GCC	71	7	2	11	5	7	4	5	7
V. carlesii × *V. macrocephalum* _38849	CGCGGGGTAGAGCAGTTTGGTAGCTCGCAAGGCTCATAACCTTGAGGTCACGGGTTCAAATCCTGTCTCCGCAA	38849	38922	0	reverse	fMet	CAU	74	6	4	9	5	7	5	5	7
V. carlesii × *V. macrocephalum* _47576	GGAGAGATGGCCGAGTGGTTCAAGGCGTAGCATTGGAACTGCTATGTAGGCTTTTGTTTACCGAGGGTTCGAATCCCTCTCTTTCCG	47576	47662	0	forward	Ser	GGA	87	7	3	11	5	7	18	5	7
V. carlesii × *V. macrocephalum* _48915	GCCTGCTTAGCTCAGAGGTTAGAGCATCGCATTTGTAATGCGATGGTCATCGGTTCGATTCCGATAGCCGGCT	48915	48987	0	reverse	Thr	UGU	73	7	4	8	5	7	5	5	7
V. carlesii × *V. macrocephalum* _49708	GGGGATATGGCGAAATTGGTAGACGCTACGGACTTAAAAAATCCGTCGACTTTAGAAATCGTGAGGGTTCAAGTCCCTCTATCCCCA	49708	50296	1	forward	Leu	UAA	87	7	3	11	5	9	16	5	7
V. carlesii × *V. macrocephalum* _50674	GTCGGGATAGCTCAGCCGGTAGAGCAGAGGACTGAAAATCCTCGTGTCACCAGTTCAAATCTGGTTCCTGGCA	50674	50746	0	forward	Phe	GAA	73	7	4	8	5	7	5	5	7
V. carlesii × *V. macrocephalum* _54156	AGGGCTATAGCTCAGTTAGGTAGAGCACCTCGTTTACACACCGAGAAGGTCTACGGTTCGAGTCCGTATAGCCCTA	54156	54805	1	reverse	Val	UAC	76	7	4	9	4	9	6	5	7
V. carlesii × *V. macrocephalum* _54982	CCTACTTAACTCAGTGGTTAGAGTATTGCTTTCATACGGCAGGAGTCATTGGTTCAAATCCAATAGTAGGTA	54982	55053	0	forward	Met	CAU	72	6	4	8	5	7	5	5	7
V. carlesii × *V. macrocephalum* _69121	GCGCTCTTAGTTCAGTTCGGTAGAACGTGGGTCTCCAAAACCCGATGTCGTAGGTTCAAATCCTACAGAGCGTG	69121	69194	0	reverse	Trp	CCA	74	7	4	9	5	7	5	5	7
V. carlesii × *V. macrocephalum* _69370	AGGGATGTGGCGCAGCTTGGTAGCGCGTTTGTTTTGGGTACAAAATGTCACGGGTTCAAATCCTGTCATCCCTA	69370	69443	0	reverse	Pro	UGG	74	7	4	9	5	7	5	5	7
V. carlesii × *V. macrocephalum* _89123	GCATCCATGGCTGAATGGTTAAAGCGCCCAACTCATAATTGGCGAATTCGTAGGTTCAATTCCTACTGGATGCA	89123	89196	0	reverse	Ile2	CAU	74	7	3	10	5	7	5	5	7
V. carlesii × *V. macrocephalum* _96895	GCCTTGGTGGTGAAATGGTAGACACGCGAGACTCAAAATCTCGTGCTAAAGAGCGTGGAGGTTCGAGTCCTCTTCAAGGCA	96895	96975	0	reverse	Leu	CAA	81	7	3	10	5	7	13	5	7
V. carlesii × *V. macrocephalum* _103281	AGGGATATAACTCAGCGGTAGAGTGTCACCTTGACGTGGTGGAAGTCATCAGTTCGAGCCTGATTATCCCTA	103281	103352	0	forward	Val	GAC	72	7	4	7	5	7	5	5	7
V. carlesii × *V. macrocephalum* _105355	GGGCTATTAGCTCAGTGGTAGAGCGCGCCCCTGATAATTGCGGGGCGAGGTCTCTGGTTCAAGTCCAGGATGGCCCA	105355	106372	1	forward	Ile	GAU	77	7	4	7	5	12	5	5	7
V. carlesii × *V. macrocephalum* _106437	GGGGATATAGCTCAGTTGGTAGAGCTCCGCTCTTGCAAGGCGGATGTCAGCGGTTCGAGTCCGCTTATCTCCA	106437	107323	1	forward	Ala	UGC	73	7	4	8	5	7	5	5	7
V. carlesii × *V. macrocephalum* _111083	GGGCCTGTAGCTCAGAGGATTAGAGCACGTGGCTACGAACCACGGTGTCGGGGGTTCGAATCCCTCCTCGCCCA	111083	111156	0	forward	Arg	ACG	74	7	4	9	5	7	5	5	7
V. carlesii × *V. macrocephalum* _111769	TCCTCAGTAGCTCAGTGGTAGAGCGGTCGGCTGTTAACCGATTGGTCGTAGGTTCGAATCCTACTTGGGGAG	111769	111840	0	reverse	Asn	GUU	72	7	4	7	5	7	5	5	7
V. carlesii × *V. macrocephalum* _118001	GCCGCTATGGTGAAATCGGTAGACACGCTGCTCTTAGGAAGCAGTGCTAGAGCATCTCGGTTCGAGTCCGAGTGGCGGCA	118001	118080	0	forward	Leu	UAG	80	7	3	11	5	7	11	5	7
V. carlesii × *V. macrocephalum* _133614	TCCTCAGTAGCTCAGTGGTAGAGCGGTCGGCTGTTAACCGATTGGTCGTAGGTTCGAATCCTACTTGGGGAG	133614	133685	0	forward	Asn	GUU	72	7	4	7	5	7	5	5	7
V. carlesii × *V. macrocephalum* _134298	GGGCCTGTAGCTCAGAGGATTAGAGCACGTGGCTACGAACCACGGTGTCGGGGGTTCGAATCCCTCCTCGCCCA	134298	134371	0	reverse	Arg	ACG	74	7	4	9	5	7	5	5	7
V. carlesii × *V. macrocephalum* _138131	GGGGATATAGCTCAGTTGGTAGAGCTCCGCTCTTGCAAGGCGGATGTCAGCGGTTCGAGTCCGCTTATCTCCA	138131	139017	1	reverse	Ala	UGC	73	7	4	8	5	7	5	5	7
V. carlesii × *V. macrocephalum* _139082	GGGCTATTAGCTCAGTGGTAGAGCGCGCCCCTGATAATTGCGGGGCGAGGTCTCTGGTTCAAGTCCAGGATGGCCCA	139082	140100	1	reverse	Ile	GAU	77	7	4	7	5	12	5	5	7
V. carlesii × *V. macrocephalum* _142102	AGGGATATAACTCAGCGGTAGAGTGTCACCTTGACGTGGTGGAAGTCATCAGTTCGAGCCTGATTATCCCTA	142102	142173	0	reverse	Val	GAC	72	7	4	7	5	7	5	5	7
V. carlesii × *V. macrocephalum* _148479	GCCTTGGTGGTGAAATGGTAGACACGCGAGACTCAAAATCTCGTGCTAAAGAGCGTGGAGGTTCGAGTCCTCTTCAAGGCA	148479	148559	0	forward	Leu	CAA	81	7	3	10	5	7	13	5	7
V. carlesii × *V. macrocephalum* _156258	GCATCCATGGCTGAATGGTTAAAGCGCCCAACTCATAATTGGCGAATTCGTAGGTTCAATTCCTACTGGATGCA	156258	156331	0	forward	Ile2	CAU	74	7	3	10	5	7	5	5	7
V. rhytidophyllum _1	GCGGATGTAGCCAAGTGGATCAAGGCAGTGGATTGTGAATCCACCATGCGCGGGTTCAATTCCCGTCGTTCGCC	1	74	0	reverse	His	GUG	74	7	3	11	5	7	5	5	7
V. rhytidophyllum _1760	GGGTTGCTAACTCAACGGTAGAGTACTCGGCTTTTAACCGACTAGTTCCGGGTTCGAATCCCGGGCAACCCA	1760	4355	1	reverse	Lys	UUU	72	7	4	7	4	7	6	5	7
V. rhytidophyllum _7863	TGGGGCGTAGCCAAGTGGTAAGGCAACGGGTTTTGGTCCCGCTATTCGGAGGTTCGAATCCTTCCGTCCCAG	7863	7934	0	reverse	Gln	UUG	72	7	3	9	4	7	6	5	7
V. rhytidophyllum _9057	GGAGAGATGGCTGAGTGGACTAAAGCGGCGGATTGCTAATCCGTTGTACGAGTTATTCGTACCGAGGGTTCGAATCCCTCTCTTTCCG	9057	9144	0	reverse	Ser	GCU	88	7	3	11	5	7	19	5	7
V. rhytidophyllum _9851	GCGGGTATAGTTTAGTGGTAAAACCTTAGCCTTCCAAGCTAACGATGCGGGTTCGATTCCCGCTACCCGC	9851	10619	1	forward	Gly	UCC	70	7	4	7	5	7	4	5	7
V. rhytidophyllum _10797	GCGTCCATTGTCTAATGGATAGGACATAGGTCTTCTAAACCTTTGGTATAGGTTCAAATCCTATTGGACGCA	10797	10868	0	forward	Arg	UCU	72	7	4	8	4	7	5	5	7
V. rhytidophyllum _29126	GGCGGCATGGCCGAGTGGTAAGGCGGGGGACTGCAAATCCTTTTTCCCCAGTTCAAATCCGGGTGTCGCCT	29126	29196	0	forward	Cys	GCA	71	7	3	9	5	7	4	5	7
V. rhytidophyllum _32548	GGGATTGTAGTTCAATTGGTCAGAGCACCGCCCTGTCAAGGCGGAAGCTGCGGGTTCGAGCCCCGTCAGTCCCG	32548	32621	0	reverse	Asp	GUC	74	7	4	9	5	7	5	5	7
V. rhytidophyllum _32731	GGGTCGATGCCCGAGCGGTTAATGGGGACGGACTGTAAATTCGTTGGCAATATGTCTACGCTGGTTCAAATCCAGCTCGGCCCA	32731	32814	0	reverse	Tyr	GUA	84	7	4	9	5	7	4	5	7
V. rhytidophyllum _32879	GCCCCCATCGTCTAGTGGTTCAGGACATCTCTCTTTCAAGGAGGCGGCGGGGATTCGACTTCCCCTGGGGGTA	32879	32951	0	reverse	Glu	UUC	73	7	4	9	5	7	4	5	7
V. rhytidophyllum _33768	GCCCCTTTAACTCAGCGGTAGAGTAACGCCATGGTAAGGCGTAAGTCATCGGTTCAAATCCGATAAGGGGCT	33768	33839	0	forward	Thr	GGU	72	7	4	7	5	7	5	5	7
V. rhytidophyllum _37669	GGAGAGATGGCTGAGTGGTTGATAGCTCCGGTCTTGAAAACCGGTATAGTTTTGAACAAAGAACTATCGAGGGTTCGAATCCCTCTCTCTCCT	37669	37761	0	reverse	Ser	UGA	93	7	4	9	5	7	24	5	7
V. rhytidophyllum _38598	GCGGATATGGTCGAATGGTAAAATTTCTCTTTGCCAAGGAGAAGACGCGGGTTCGATTCCCGCTATCCGCC	38598	38668	0	forward	Gly	GCC	71	7	2	11	5	7	4	5	7
V. rhytidophyllum _38842	CGCGGGGTAGAGCAGTTTGGTAGCTCGCAAGGCTCATAACCTTGAGGTCACGGGTTCAAATCCTGTCTCCGCAA	38842	38915	0	reverse	fMet	CAU	74	6	4	9	5	7	5	5	7
V. rhytidophyllum _47569	GGAGAGATGGCCGAGTGGTTCAAGGCGTAGCATTGGAACTGCTATGTAGGCTTTTGTTTACCGAGGGTTCGAATCCCTCTCTTTCCG	47569	47655	0	forward	Ser	GGA	87	7	3	11	5	7	18	5	7
V. rhytidophyllum _48909	GCCTGCTTAGCTCAGAGGTTAGAGCATCGCATTTGTAATGCGATGGTCATCGGTTCGATTCCGATAGCCGGCT	48909	48981	0	reverse	Thr	UGU	73	7	4	8	5	7	5	5	7
V. rhytidophyllum _49701	GGGGATATGGCGAAATTGGTAGACGCTACGGACTTAAAAAATCCGTCGACTTTAGAAATCGTGAGGGTTCAAGTCCCTCTATCCCCA	49701	50291	1	forward	Leu	UAA	85	7	3	11	5	9	16	5	7
V. rhytidophyllum _50670	GTCGGGATAGCTCAGCCGGTAGAGCAGAGGACTGAAAATCCTCGTGTCACCAGTTCAAATCTGGTTCCTGGCA	50670	50742	0	forward	Phe	GAA	73	7	4	8	5	7	5	5	7
V. rhytidophyllum _54151	AGGGCTATAGCTCAGTTAGGTAGAGCACCTCGTTTACACACCGAGAAGGTCTACGGTTCGAGTCCGTATAGCCCTA	54151	54800	1	reverse	Val	UAC	76	7	4	9	4	9	6	5	7
V. rhytidophyllum _54977	CCTACTTAACTCAGTGGTTAGAGTATTGCTTTCATACGGCAGGAGTCATTGGTTCAAATCCAATAGTAGGTA	54977	55048	0	forward	Met	CAU	72	6	4	8	5	7	5	5	7
V. rhytidophyllum _69118	GCGCTCTTAGTTCAGTTCGGTAGAACGTGGGTCTCCAAAACCCGATGTCGTAGGTTCAAATCCTACAGAGCGTG	69118	69191	0	reverse	Trp	CCA	74	7	4	9	5	7	5	5	7
V. rhytidophyllum _69367	AGGGATGTGGCGCAGCTTGGTAGCGCGTTTGTTTTGGGTACAAAATGTCACGGGTTCAAATCCTGTCATCCCTA	69367	69440	0	reverse	Pro	UGG	74	7	4	9	5	7	5	5	7
V. rhytidophyllum _89117	GCATCCATGGCTGAATGGTTAAAGCGCCCAACTCATAATTGGCGAATTCGTAGGTTCAATTCCTACTGGATGCA	89117	89190	0	reverse	Ile2	CAU	74	7	3	10	5	7	5	5	7
V. rhytidophyllum _96895	GCCTTGGTGGTGAAATGGTAGACACGCGAGACTCAAAATCTCGTGCTAAAGAGCGTGGAGGTTCGAGTCCTCTTCAAGGCA	96895	96975	0	reverse	Leu	CAA	81	7	3	10	5	7	13	5	7
V. rhytidophyllum _103281	AGGGATATAACTCAGCGGTAGAGTGTCACCTTGACGTGGTGGAAGTCATCAGTTCGAGCCTGATTATCCCTA	103281	103352	0	forward	Val	GAC	72	7	4	7	5	7	5	5	7
V. rhytidophyllum _105355	GGGCTATTAGCTCAGTGGTAGAGCGCGCCCCTGATAATTGCGGGGCGAGGTCTCTGGTTCAAGTCCAGGATGGCCCA	105355	106373	1	forward	Ile	GAU	77	7	4	7	5	12	5	5	7
V. rhytidophyllum _106438	GGGGATATAGCTCAGTTGGTAGAGCTCCGCTCTTGCAAGGCGGATGTCAGCGGTTCGAGTCCGCTTATCTCCA	106438	107325	1	forward	Ala	UGC	73	7	4	8	5	7	5	5	7
V. rhytidophyllum _111085	GGGCCTGTAGCTCAGAGGATTAGAGCACGTGGCTACGAACCACGGTGTCGGGGGTTCGAATCCCTCCTCGCCCA	111085	111158	0	forward	Arg	ACG	74	7	4	9	5	7	5	5	7
V. rhytidophyllum _111771	TCCTCAGTAGCTCAGTGGTAGAGCGGTCGGCTGTTAACCGATTGGTCGTAGGTTCGAATCCTACTTGGGGAG	111771	111842	0	reverse	Asn	GUU	72	7	4	7	5	7	5	5	7
V. rhytidophyllum _117997	GCCGCTATGGTGAAATCGGTAGACACGCTGCTCTTAGGAAGCAGTGCTAGAGCATCTCGGTTCGAGTCCGAGTGGCGGCA	117997	118076	0	forward	Leu	UAG	80	7	3	11	5	7	11	5	7
V. rhytidophyllum _133618	TCCTCAGTAGCTCAGTGGTAGAGCGGTCGGCTGTTAACCGATTGGTCGTAGGTTCGAATCCTACTTGGGGAG	133618	133689	0	forward	Asn	GUU	72	7	4	7	5	7	5	5	7
V. rhytidophyllum _134302	GGGCCTGTAGCTCAGAGGATTAGAGCACGTGGCTACGAACCACGGTGTCGGGGGTTCGAATCCCTCCTCGCCCA	134302	134375	0	reverse	Arg	ACG	74	7	4	9	5	7	5	5	7
V. rhytidophyllum _138135	GGGGATATAGCTCAGTTGGTAGAGCTCCGCTCTTGCAAGGCGGATGTCAGCGGTTCGAGTCCGCTTATCTCCA	138135	139022	1	reverse	Ala	UGC	73	7	4	8	5	7	5	5	7
V. rhytidophyllum _139087	GGGCTATTAGCTCAGTGGTAGAGCGCGCCCCTGATAATTGCGGGGCGAGGTCTCTGGTTCAAGTCCAGGATGGCCCA	139087	140105	1	reverse	Ile	GAU	77	7	4	7	5	12	5	5	7
V. rhytidophyllum _142108	AGGGATATAACTCAGCGGTAGAGTGTCACCTTGACGTGGTGGAAGTCATCAGTTCGAGCCTGATTATCCCTA	142108	142179	0	reverse	Val	GAC	72	7	4	7	5	7	5	5	7
V. rhytidophyllum _148485	GCCTTGGTGGTGAAATGGTAGACACGCGAGACTCAAAATCTCGTGCTAAAGAGCGTGGAGGTTCGAGTCCTCTTCAAGGCA	148485	148565	0	forward	Leu	CAA	81	7	3	10	5	7	13	5	7
V. rhytidophyllum _156270	GCATCCATGGCTGAATGGTTAAAGCGCCCAACTCATAATTGGCGAATTCGTAGGTTCAATTCCTACTGGATGCA	156270	156343	0	forward	Ile2	CAU	74	7	3	10	5	7	5	5	7
							Average:	76	7	4	9	5	7	7	5	7
							Minimum:	70	5	2	7	4	7	4	5	7
							Maximum:	93	7	4	11	5	12	24	5	7
