# Supplementary material for: Phylogeny and evolution of chloroplast tRNAs in Adoxaceae
Source: Ecol Evol. 2021 Jan 6;11(3):1294–309. doi: 10.1002/ece3.7133 (PMC7863635; doi:10.1002/ece3.7133)
Supplement: Supplementary file 3 — Table S2 [file ECE3-11-1294-s003.pdf]

Supplementary Table 2

Supplementary Table 2-a

The number of duplication, loss, co-divergence and transfer events, and their related species. Analysis results by NOTUNG 2.9.

Species	Duplications	Co-Divergences	Transfers_from	Transfers_to	Losses
>1 sp. Adoxaceae	20	5	0	0	7
V. utile	6	0	4	21	4
T. omeiensis	7	0	24	3	7
n41	14	0	0	0	0
A. moschatellina	0	0	0	3	0
S. corydalifolia	1	0	1	3	1
S. williamsii	6	0	26	25	4
n39	6	0	0	0	6
Total	60	5	55	55	29

Supplementary Table 2-b

The number of transfer events, and the related recipient species and donor species.

Analysis results by NOTUNG 2.9.

To\From(sp.)	S. corydalifolia	A. moschatellina	T. omeiensis	S. williamsii	V. utile
S. corydalifolia	-	0	0	2	1
A. moschatellina	1	-	0	1	1
T. omeiensis	0	0	-	2	1
S. williamsii	0	0	24	-	1
V. utile	0	0	0	21	-

Supplementary Table 2-c

Loss events of chloroplast tRNA genes and species in which they lost.

Analysis results by NOTUNG 2.9.

Lost in Species	tRNA Gene	Species	isotype	Anti-codon
T. omeiensis	A. moschatellina_102545	*A. moschatellina*	Val	GAC
	A. moschatellina_140963	*A. moschatellina*	Val	GAC
	T. omeiensis_102753	*T. omeiensis*	Val	GAC
	T. omeiensis_141205	*T. omeiensis*	Val	GAC
	S. williamsii_103115	*S. williamsii*	Val	GAC
	S. williamsii_141930	*S. williamsii*	Val	GAC
	V. utile_102789	*V. utile*	Val	GAC
	V. utile_141340	*V. utile*	Val	GAC
n39	S. corydalifolia_102483	*S. corydalifolia*	Val	GAC
V. utile,	S. corydalifolia_102483	*S. corydalifolia*	Val	GAC

S. williamsii	S. corydalifolia_140951	*S. corydalifolia*	Val	GAC
	A. moschatellina_102545	*A. moschatellina*	Val	GAC
	A. moschatellina_140963	*A. moschatellina*	Val	GAC
	T. omeiensis_102753	*T. omeiensis*	Val	GAC
	T. omeiensis_141205	*T. omeiensis*	Val	GAC
	S. williamsii_103115	*S. williamsii*	Val	GAC
	S. williamsii_141930	*S. williamsii*	Val	GAC
	V. utile_102789	*V. utile*	Val	GAC
	V. utile_141340	*V. utile*	Val	GAC
	S. corydalifolia_53253	*S. corydalifolia*	Val	UAC
	A. moschatellina_53131	*A. moschatellina*	Val	UAC
	T. omeiensis_53193	*T. omeiensis*	Val	UAC
	S. williamsii_53583	*S. williamsii*	Val	UAC
	V. utile_53289	*V. utile*	Val	UAC
	S. corydalifolia_105633	*S. corydalifolia*	Ala	UGC
	S. corydalifolia_136985	*S. corydalifolia*	Ala	UGC
	A. moschatellina_105701	*A. moschatellina*	Ala	UGC
	A. moschatellina_136991	*A. moschatellina*	Ala	UGC
	T. omeiensis_105909	*T. omeiensis*	Ala	UGC
	T. omeiensis_137233	*T. omeiensis*	Ala	UGC
	S. williamsii_106272	*S. williamsii*	Ala	UGC
	S. williamsii_137957	*S. williamsii*	Ala	UGC
	V. utile_105946	*V. utile*	Ala	UGC
	V. utile_137367	*V. utile*	Ala	UGC
	S. corydalifolia_31544	*S. corydalifolia*	Asp	GUC
	A. moschatellina_31464	*A. moschatellina*	Asp	GUC
	T. omeiensis_31515	*T. omeiensis*	Asp	GUC
	S. williamsii_31763	*S. williamsii*	Asp	GUC
	V. utile_31685	*V. utile*	Asp	GUC
	S. corydalifolia_54095	*S. corydalifolia*	Met	CAU
	A. moschatellina_53974	*A. moschatellina*	Met	CAU
	T. omeiensis_54032	*T. omeiensis*	Met	CAU
	S. williamsii_54418	*S. williamsii*	Met	CAU
	V. utile_54115	*V. utile*	Met	CAU
	S. corydalifolia_47633	*S. corydalifolia*	Thr	UGU
	A. moschatellina_47541	*A. moschatellina*	Thr	UGU
S. williamsii	T. omeiensis_47591	*T. omeiensis*	Thr	UGU
	S. williamsii_48092	*S. williamsii*	Thr	UGU
	V. utile_48048	*V. utile*	Thr	UGU
	S. corydalifolia_32668	*S. corydalifolia*	Thr	GGU
	A. moschatellina_32589	*A. moschatellina*	Thr	GGU
	T. omeiensis_32640	*T. omeiensis*	Thr	GGU
	S. williamsii_32967	*S. williamsii*	Thr	GGU
	V. utile_32906	*V. utile*	Thr	GGU

V. utile, S. williamsii	S. corydalifolia_48473	*S. corydalifolia*	Leu	UAA
	A. moschatellina_48385	*A. moschatellina*	Leu	UAA
	T. omeiensis_48411	*T. omeiensis*	Leu	UAA
	S. williamsii_48956	*S. williamsii*	Leu	UAA
	V. utile_48840	*V. utile*	Leu	UAA
	S. corydalifolia_8887	*S. corydalifolia*	Ser	GCU
	S. corydalifolia_36817	*S. corydalifolia*	Ser	UGA
	A. moschatellina_8841	*A. moschatellina*	Ser	GCU
	A. moschatellina_36732	*A. moschatellina*	Ser	UGA
	T. omeiensis_8919	*T. omeiensis*	Ser	GCU
	T. omeiensis_36777	*T. omeiensis*	Ser	UGA
	S. williamsii_9088	*S. williamsii*	Ser	GCU
	S. williamsii_37050	*S. williamsii*	Ser	UGA
	V. utile_9059	*V. utile*	Ser	GCU
	V. utile_36807	*V. utile*	Ser	UGA
V. utile	S. corydalifolia_38018	*S. corydalifolia*	fMet	CAU
	A. moschatellina_37933	*A. moschatellina*	fMet	CAU
	T. omeiensis_37979	*T. omeiensis*	fMet	CAU
	S. williamsii_38226	*S. williamsii*	fMet	CAU
	V. utile_37980	*V. utile*	fMet	CAU
S. williamsii	S. corydalifolia_68493	*S. corydalifolia*	Pro	UGG
	A. moschatellina_68378	*A. moschatellina*	Pro	UGG
	T. omeiensis_68668	*T. omeiensis*	Pro	UGG
	S. williamsii_69164	*S. williamsii*	Pro	UGG
	V. utile_68872	*V. utile*	Pro	UGG
T. omeiensis	A. moschatellina_104620	*A. moschatellina*	Ile	GAU
	A. moschatellina_110341	*A. moschatellina*	Arg	ACG
n39	S. corydalifolia_104558	*S. corydalifolia*	Ile	GAU
	S. corydalifolia_110289	*S. corydalifolia*	Arg	ACG
V. utile, S. williamsii	S. corydalifolia_104558	*S. corydalifolia*	Ile	GAU
	S. corydalifolia_137931	*S. corydalifolia*	Ile	GAU
	A. moschatellina_104620	*A. moschatellina*	Ile	GAU
	A. moschatellina_137942	*A. moschatellina*	Ile	GAU
	T. omeiensis_104828	*T. omeiensis*	Ile	GAU
	T. omeiensis_138184	*T. omeiensis*	Ile	GAU
	S. williamsii_105190	*S. williamsii*	Ile	GAU
	S. williamsii_138908	*S. williamsii*	Ile	GAU
	V. utile_104863	*V. utile*	Ile	GAU
	V. utile_138319	*V. utile*	Ile	GAU
	S. corydalifolia_1839	*S. corydalifolia*	Lys	UUU
	A. moschatellina_1841	*A. moschatellina*	Lys	UUU
	T. omeiensis_1911	*T. omeiensis*	Lys	UUU
	S. williamsii_1819	*S. williamsii*	Lys	UUU
	V. utile_1763	*V. utile*	Lys	UUU

	S. corydalifolia_110289	*S. corydalifolia*	Arg	ACG
	S. corydalifolia_133143	*S. corydalifolia*	Arg	ACG
	A. moschatellina_110341	*A. moschatellina*	Arg	ACG
	A. moschatellina_133165	*A. moschatellina*	Arg	ACG
	T. omeiensis_110558	*T. omeiensis*	Arg	ACG
	T. omeiensis_133398	*T. omeiensis*	Arg	ACG
	S. williamsii_110920	*S. williamsii*	Arg	ACG
	S. williamsii_134123	*S. williamsii*	Arg	ACG
	V. utile_110593	*V. utile*	Arg	ACG
	V. utile_133534	*V. utile*	Arg	ACG
T. omeiensis	A. moschatellina_7675	*A. moschatellina*	Gln	UUG
S. corydalifolia	A. moschatellina_7676	*A. moschatellina*	Gln	UUG
S. williamsii	S. corydalifolia_49559	*S. corydalifolia*	Phe	GAA
	A. moschatellina_49526	*A. moschatellina*	Phe	GAA
	T. omeiensis_49590	*T. omeiensis*	Phe	GAA
	S. williamsii_49928	*S. williamsii*	Phe	GAA
	V. utile_49808	*V. utile*	Phe	GAA
	S. corydalifolia_28834	*S. corydalifolia*	Cys	GCA
	A. moschatellina_28782	*A. moschatellina*	Cys	GCA
	T. omeiensis_28829	*T. omeiensis*	Cys	GCA
	S. williamsii_29055	*S. williamsii*	Cys	GCA
	V. utile_28913	*V. utile*	Cys	GCA
	S. corydalifolia_31723	*S. corydalifolia*	Tyr	GUA
	A. moschatellina_31642	*A. moschatellina*	Tyr	GUA
	T. omeiensis_31693	*T. omeiensis*	Tyr	GUA
	S. williamsii_31941	*S. williamsii*	Tyr	GUA
	V. utile_31868	*V. utile*	Tyr	GUA
V. utile	S. corydalifolia_28834	*S. corydalifolia*	Cys	GCA
	A. moschatellina_28782	*A. moschatellina*	Cys	GCA
	T. omeiensis_28829	*T. omeiensis*	Cys	GCA
	S. williamsii_29055	*S. williamsii*	Cys	GCA
	V. utile_28913	*V. utile*	Cys	GCA
	S. corydalifolia_31723	*S. corydalifolia*	Tyr	GUA
	A. moschatellina_31642	*A. moschatellina*	Tyr	GUA
	T. omeiensis_31693	*T. omeiensis*	Tyr	GUA
	S. williamsii_31941	*S. williamsii*	Tyr	GUA
	V. utile_31868	*V. utile*	Tyr	GUA
T. omeiensis	A. moschatellina_132489	*A. moschatellina*	Asn	GUU
	A. moschatellina_155141	*A. moschatellina*	Ile2	CAU
n39	S. corydalifolia_132467	*S. corydalifolia*	Asn	GUU
	S. corydalifolia_154985	*S. corydalifolia*	Ile2	CAU
V. utile, S. williamsii	S. corydalifolia_88447	*S. corydalifolia*	Ile2	CAU
	S. corydalifolia_154985	*S. corydalifolia*	Ile2	CAU
	A. moschatellina_88365	*A. moschatellina*	Ile2	CAU

	A. moschatellina_155141	*A. moschatellina*	Ile2	CAU
	T. omeiensis_88597	*T. omeiensis*	Ile2	CAU
	T. omeiensis_155359	*T. omeiensis*	Ile2	CAU
	S. williamsii_88952	*S. williamsii*	Ile2	CAU
	S. williamsii_156091	*S. williamsii*	Ile2	CAU
	V. utile_88625	*V. utile*	Ile2	CAU
	V. utile_155502	*V. utile*	Ile2	CAU
	S. corydalifolia_110967	*S. corydalifolia*	Asn	GUU
	S. corydalifolia_132467	*S. corydalifolia*	Asn	GUU
	A. moschatellina_111019	*A. moschatellina*	Asn	GUU
	A. moschatellina_132489	*A. moschatellina*	Asn	GUU
	T. omeiensis_111236	*T. omeiensis*	Asn	GUU
	T. omeiensis_132722	*T. omeiensis*	Asn	GUU
	S. williamsii_111597	*S. williamsii*	Asn	GUU
	S. williamsii_133448	*S. williamsii*	Asn	GUU
	V. utile_111279	*V. utile*	Asn	GUU
	V. utile_132850	*V. utile*	Asn	GUU
	S. corydalifolia_68258	*S. corydalifolia*	Trp	CCA
	A. moschatellina_68143	*A. moschatellina*	Trp	CCA
	T. omeiensis_68433	*T. omeiensis*	Trp	CCA
	S. williamsii_68929	*S. williamsii*	Trp	CCA
	V. utile_68623	*V. utile*	Trp	CCA
S. williamsii	S. corydalifolia_10668	*S. corydalifolia*	Arg	UCU
	A. moschatellina_10639	*A. moschatellina*	Arg	UCU
	T. omeiensis_10700	*T. omeiensis*	Arg	UCU
	S. williamsii_10898	*S. williamsii*	Arg	UCU
	V. utile_10799	*V. utile*	Arg	UCU
	S. corydalifolia_31871	*S. corydalifolia*	Glu	UUC
	A. moschatellina_31790	*A. moschatellina*	Glu	UUC
	T. omeiensis_31841	*T. omeiensis*	Glu	UUC
	S. williamsii_32089	*S. williamsii*	Glu	UUC
	V. utile_32016	*V. utile*	Glu	UUC
V. utile	S. corydalifolia_31871	*S. corydalifolia*	Glu	UUC
	A. moschatellina_31790	*A. moschatellina*	Glu	UUC
	T. omeiensis_31841	*T. omeiensis*	Glu	UUC
	S. williamsii_32089	*S. williamsii*	Glu	UUC
	V. utile_32016	*V. utile*	Glu	UUC
V. utile, S. williamsii	S. corydalifolia_116738	*S. corydalifolia*	Leu	UAG
	A. moschatellina_116790	*A. moschatellina*	Leu	UAG
	T. omeiensis_116957	*T. omeiensis*	Leu	UAG
	S. williamsii_117749	*S. williamsii*	Leu	UAG
	V. utile_117229	*V. utile*	Leu	UAG
V. utile	S. williamsii_96741	*S. williamsii*	Leu	CAA
T. omeiensis	A. moschatellina_96140	*A. moschatellina*	Leu	CAA

n39	S. corydalifolia_96228	*S. corydalifolia*	Leu	CAA

Supplementary Table 2-d

The number of speciation, duplication, loss and transfer events, and their related species. Analysis results by GeneRax v1.2.2.

Species	Speciation	Duplications	Transfers_from	Transfers_to	Losses
V. utile	37	0	0	0	0
T. omeiensis	37	1	0	0	0
A. moschatellina	37	1	0	0	0
S. corydalifolia	37	1	0	0	0
S. williamsii	37	0	0	0	0
n0	37	0	0	0	2
n1	37	1	0	0	1
n2	37	0	0	0	1
n3	37	36	0	0	0
Total	60	5	55	55	29
